# Supplementary material for: Design, Synthesis and Antifungal/Nematicidal Activity of Novel 1,2,4-Oxadiazole Derivatives Containing Amide Fragments
Source: Int J Mol Sci. 2022 Jan 29;23(3):1596. doi: 10.3390/ijms23031596 (PMC8836147; doi:10.3390/ijms23031596)
Supplement: Supplementary file 1 [file ijms-23-01596-s001.zip › ijms-1538108-SM.pdf]

International Journal of *Molecular Sciences*

MDPI

Supplementary Materials

Design, Synthesis and Antifungal/Nematicidal Activity of Novel 1,2,4-Oxadiazole Derivatives Containing Amide Fragments

Dan Liu [1], Ling Luo [1], Zhengxing Wang [1], Xiaoyun Ma [2] and Xiuhai Gan [1,*]

1 State Key Laboratory Breeding Base of Green Pesticide and Agricultural Bioengineering, Key Laboratory of Green Pesticide and Agricultural Bioengineering, Ministry of Education, Research and Development Center for Fine Chemicals, Guizhou University, Huaxi District, Guiyang 550025, China; liudan19960516@163.com (D.L.); ll17792015@163.com (L.L.); wzx2669452150@163.com (Z.W.)

2 School of Chemistry and Materials Science, Guizhou Education University, Wudang District, Guiyang 550018, China; maxiaoyun821215@163.com

* Correspondence: gxh200719@163.com

1. The characterization data of the not known intermediated

The characterization data of the not known intermediated including ^{1}H NMR and ^{13}C NMR were shown as below.

Data for 3-Chloro-*N*-hydroxy-5-(trifluoromethyl)picolinimidamide (**a6**), m.p. 120–121 ℃, yield 86.7%, ^{1}H NMR (400 MHz, DMSO-*d6*) δ 10.05 (s, 1H), 8.98 (d, J = 1.2 Hz, 1H), 8.53 (d, J = 1.2 Hz, 1H), 5.97 (s, 2H). ^{13}C NMR (101 MHz, DMSO-*d6*) δ 153.08, 149.43, 144.16 (d, J = 4.0 Hz), 136.51 (d, J = 3.5 Hz), 130.53, 126.27 (d, J = 33.1 Hz), 123.13 (d, J = 271.4 Hz).

Data for Ethyl 3-(3-chloro-5-(trifluoromethyl)pyridin-2-yl)-1,2,4-oxadiazole-5-carboxylate (**b6**), white solid, m.p. 43–44 ℃, yield 75.8%, ^{1}H NMR (400 MHz, $CDCl_3$) δ 8.97 (d, J = 1.2 Hz, 1H), 8.19 (d, J = 1.6 Hz, 1H), 4.59 (q, J = 7.2 Hz, 2H), 1.50 (t, J = 7.2 Hz, 3H). ^{13}C NMR (101 MHz, $CDCl_3$) δ 166.99, 166.90, 153.65, 146.37, 144.71 (d, J = 4.0 Hz), 136.21 (d, J = 3.5 Hz), 132.64, 129.66–128.46 (m), 122.10 (q, J = 274.8 Hz), 64.21, 13.99.

Data for 3-(4-Thlorophenyl)-1,2,4-oxadiazole-5-carboxylic acid (**c3**), white solid, m.p. 85–86 ℃, yield 56.7%, ^{1}H NMR (400 MHz, DMSO-*d6*) δ 9.77 (s, 1H), 8.06 (d, J = 8.4 Hz, 2H), 7.67 (d, J = 8.4 Hz, 2H). ^{13}C NMR (101 MHz, DMSO-*d6*) δ 168.16, 166.50, 136.87, 134.61, 129.97, 129.97, 129.40, 129.40, 125.22.

Data for 3-(Thiophen-2-yl)-1,2,4-oxadiazole-5-carboxylic acid (**c4**), m.p. 76–77 ℃, yield 60.3%, ^{1}H NMR (400 MHz, DMSO-*d6*) δ 11.17 (s, 1H), 8.09 (d, J = 6.2 Hz, 1H), 7.99 (d, J = 3.8 Hz, 1H), 7.30 (dd, J = 5.0, 3.8 Hz, 1H). ^{13}C NMR (101 MHz, DMSO-*d6*) δ 150.43, 139.43, 135.49, 128.81, 124.67, 114.99, 108.73.

Data for 3-(6-Bromopyridin-3-yl)-1,2,4-oxadiazole-5-carboxylic acid (**c5**) m.p. 111–112 ℃, yield 57.8%, ^{1}H NMR (400 MHz, DMSO-*d6*) δ 11.16 (s, 1H), 8.92–8.87 (m, 1H), 8.27 (dd, J = 8.3, 2.4 Hz, 1H), 7.98–7.90 (m, 1H). ^{13}C NMR (101 MHz, DMSO-*d6*) δ 153.94, 150.45, 146.29, 142.99, 129.19, 124.67, 116.78, 109.30.

Data for 3-(3-Chloro-5-(trifluoromethyl)pyridin-2-yl)-1,2,4-oxadiazole-5-carboxylic acid (**c6**) m.p. 60–61 ℃, yield 50.9%, ^{1}H NMR (400 MHz, DMSO-*d6*) δ 10.57 (s, 1H), 9.16 (d, J = 0.8 Hz, 1H), 8.90 (d, J = 0.8 Hz, 1H). ^{13}C NMR (101 MHz, DMSO-*d6*) δ 150.42, 146.42 (d, J = 4.0 Hz), 136.74 (d, J = 3.7 Hz), 136.09, 129.45 (d, J = 19.8 Hz), 124.67, 123.88, 121.15, 114.95.

2. The characterization data of the target compound

The characterization data of the target compound including ^{1}H NMR, ^{13}C NMR and HRMS were shown as below.

Data for 3-Phenyl-*N*-(2-(pyridin-2-yl)ethyl)-1,2,4-oxadiazole-5-carboxamide (**F1**), white solid, m.p. 110–112 ℃, yield 67.3%, purity 96.1%; ^{1}H NMR (400 MHz, $CDCl_3$) δ 8.71 (s, 2H), 8.28 (s, 1H), 8.08 (d, J = 6.0 Hz, 2H), 7.78 (d, J = 36.0 Hz, 2H), 7.49 (d, J = 6.8 Hz, 3H), 4.10 (s, 2H), 3.67 (s, 2H). ^{13}C NMR (101 MHz, $CDCl_3$) δ 168.88, 168.73, 157.49, 153.41, 146.56, 140.00, 131.84, 129.08, 129.08, 127.68, 127.68, 125.87, 125.14, 123.10, 39.08, 34.95. HRMS (ESI): calcd for $C_{16}H_{15}N_4O_2$ ([M+H]$^+$), 295.11895; found, 295.11966.

Data for *N*-(2-(3-chloro-5-(trifluoromethyl)pyridin-2-yl)ethyl)-3-phenyl-1,2,4-oxadiazole-5-carboxamide (**F2**), white solid, m.p. 81–82 ℃, yield 52.6%, purity 99.0%; ^{1}H NMR (400 MHz, $CDCl_3$) δ 8.79 (d, *J* = 0.8 Hz, 1H), 8.09 (dd, *J* = 7.6, 1.2 Hz, 3H), 7.94 (d, *J* = 1.2 Hz, 1H), 7.56–7.47 (m, 3H), 4.07 (dd, *J* = 12.0, 6.0 Hz, 2H), 3.35 (t, *J* = 5.6 Hz, 2H). ^{13}C NMR (101 MHz, $CDCl_3$) δ 168.76, 168.66, 160.20,

153.05, 143.78 (d, *J* = 4.1 Hz), 134.01 (d, *J* = 3.5 Hz), 131.95, 131.81, 129.01, 129.01, 127.55, 127.55, 126.73, 125.74, 123.85 (dd, *J* = 239.37, 33.84 Hz), 36.78, 33.93. HRMS (ESI): calcd for $C_{17}H_{13}ClF_3N_4O_2$ ([M+H]$^+$), 396.06736; found, 396.06738.

Data for *N*-(2,4-difluorobenzyl)-3-phenyl-1,2,4-oxadiazole-5-carboxamide (**F3**), white solid, m.p. 92–93 ℃, yield 52.9%, purity 98.7%; ^{1}H NMR (400 MHz, $CDCl_3$) δ 8.07 (d, *J* = 6.8 Hz, 2H), 7.60 (s, 1H), 7.53–7.42 (m, 4H), 6.87 (dd, *J* = 18.0, 8.8 Hz, 2H), 4.69 (d, *J* = 6.0 Hz, 2H). ^{13}C NMR (101 MHz, $CDCl_3$) δ 168.77, 168.38, 163.26 (dd, *J* = 174.9, 12.1 Hz), 160.78 (dd, *J* = 174.5, 12.1 Hz), 153.07, 131.87, 131.62 (dd, *J* = 9.9, 5.7 Hz), 129.03, 129.03, 127.55, 127.55, 125.65, 119.65 (dd, *J* = 15.2, 3.8 Hz), 111.73 (dd, *J* = 21.3, 3.8 Hz), 104.20, 37.50. HRMS (ESI): calcd for $C_{16}H_{13}F_2N_3O_2$ ([M+H]$^+$), 316.08921; found, 316.08899.

Data for *N*-(2,4-dichlorophenyl)-3-phenyl-1,2,4-oxadiazole-5-carboxamide (**F4**), white solid, m.p. 163–164 ℃, yield 50.0%, purity 98.4%; ^{1}H NMR (400 MHz, $CDCl_3$) δ 9.45 (s, 1H), 8.48 (d, *J* = 8.8 Hz, 1H), 8.14 (d, *J* = 6.8 Hz, 2H), 7.60–7.50 (m, 3H), 7.48 (d, *J* = 2.0 Hz, 1H), 7.35 (dd, *J* = 8.8, 2.0 Hz, 1H). ^{13}C NMR (101 MHz, $CDCl_3$) δ 168.88, 168.27, 150.43, 132.06, 131.88, 131.03, 129.21, 129.11, 129.11, 128.32, 127.65, 127.65, 125.44, 124.10, 122.25. HRMS (ESI): calcd for $C_{15}H_9Cl_2N_3O_2Na$ ([M+Na]$^+$), 355.99640; found, 355.99606.

Data for *N*-(2-(pyridin-2-yl)ethyl)-3-(p-tolyl)-1,2,4-oxadiazole-5-carboxamide (**F5**), yellow solid, m.p. 126–128 ℃, yield 43.3%, purity 97.3%; ^{1}H NMR (400 MHz, $CDCl_3$) δ 8.61 (d, *J* = 4.4 Hz, 1H), 8.56 (s, 1H), 7.99 (d, *J* = 8.0 Hz, 2H), 7.65 (td, *J* = 7.6, 1.6 Hz, 1H), 7.30 (d, *J* = 8.0 Hz, 2H), 7.21 (dd, *J* = 7.6, 0.8 Hz, 2H), 3.94 (dd, *J* = 12.4, 6.0 Hz, 2H), 3.14 (t, *J* = 6.0 Hz, 2H), 2.42 (s, 3H). ^{13}C NMR (101 MHz, $CDCl_3$) δ 167.40, 157.57, 151.69, 147.89, 140.76, 135.49, 128.29, 128.29, 126.12, 126.12, 122.13, 122.13, 121.72, 120.52, 37.51, 34.59, 20.23. HRMS (ESI): calcd for $C_{17}H_{17}N_4O_2$ ([M+H]$^+$), 309.13460; found, 309.13394.

Data for *N*-(2-(3-chloro-5-(trifluoromethyl)pyridin-2-yl)ethyl)-3-(p-tolyl)-1,2,4-oxadiazole-5-carboxamid-
e (**F6**), white solid, m.p. 96–98 ℃, yield 58.1%, purity 98.9%; ^{1}H NMR (400 MHz, $CDCl_3$) δ 8.79 (s, 1H), 8.05 (s, 1H), 7.97 (d, *J* = 8.2 Hz, 2H), 7.93 (t, *J* = 5.6 Hz, 1H), 7.30 (d, *J* = 8.0 Hz, 2H), 4.06 (dd, *J* = 12.1, 6.1 Hz, 2H), 3.35 (t, *J* = 5.9 Hz, 2H), 2.43 (s, 3H). ^{13}C NMR (101 MHz, $CDCl_3$) δ 168.78, 168.53, 160.22, 153.13, 143.79 (d, J = 4.1 Hz), 142.27, 134.01 (d, J = 3.5 Hz), 131.95, 129.71, 127.49, 126.42, 126.08, 124.04, 122.96, 121.32, 36.80, 33.96, 21.62. HRMS (ESI): calcd for $C_{18}H_{15}ClF_3N_4O_2$ ([M+H]$^+$), 411.08301; found, 411.08237.

Data for *N*-(2,4-difluorobenzyl)-3-(p-tolyl)-1,2,4-oxadiazole-5-carboxamide (**F7**), white solid, m.p. 119–120 ℃, yield 45.9%, purity 99.2%; ^{1}H NMR (400 MHz, $CDCl_3$) δ 7.96 (d, *J* = 8.4 Hz, 2H), 7.55 (s, 1H), 7.48–7.40 (m, 1H), 7.29 (d, *J* = 8.0 Hz, 2H), 6.93–6.83 (m, 2H), 4.69 (d, *J* = 6.2 Hz, 2H), 2.42 (s, 3H). ^{13}C NMR (101 MHz, $CDCl_3$) δ 168.78, 168.20, 164.17, 162.39 (d, *J* = 12.2 Hz), 161.69, 159.91 (d, *J* = 12.1 Hz), 153.13, 142.39, 131.64 (dd, *J* = 9.7, 5.5 Hz), 129.75, 127.49, 122.80, 119.66 (d, *J* = 19.0 Hz), 111.74 (dd, *J* = 21.6, 3.8 Hz), 104.21, 37.44, 21.65. HRMS (ESI): calcd for $C_{17}H_{14}F_2N_3O_2$ ([M+H]$^+$), 330.10486; found, 330.10410.

Data for *N*-(2,4-dichlorophenyl)-3-(p-tolyl)-1,2,4-oxadiazole-5-carboxamide (**F8**), white solid, m.p. 163–165 ℃, yield 62.8%, purity 99.1%; ^{1}H NMR (400 MHz, $CDCl_3$) δ 9.43 (s, 1H), 8.48 (d, *J* = 8.8 Hz, 1H), 8.02 (d, *J* = 8.4 Hz, 2H), 7.48 (d, *J* = 2.4 Hz, 1H), 7.34 (dd, *J* = 10.0, 2.4 Hz, 3H), 2.44 (s, 3H). ^{13}C NMR (101 MHz, $CDCl_3$) δ 168.89, 168.11, 150.50, 142.59, 131.91, 130.98, 129.80, 129.80, 129.20, 128.30, 127.58, 127.58, 124.09, 122.60, 122.25, 21.67. HRMS (ESI): calcd for $C_{16}H_{11}C_{12}N_3O_2Na$ ([M+Na]$^+$), 370.01205; found, 370.01202.

Data for 3-(4-chlorophenyl)-*N*-(2-(pyridin-2-yl)ethyl)-1,2,4-oxadiazole-5-carboxamide (**F9**), white solid, m.p. 129–131 ℃, yield 55.5%, purity 98.4%; ^{1}H NMR (400 MHz, $CDCl_3$) δ 8.67 (s, 1H, Py-H), 8.63–8.58 (m, 1H), 8.10–8.01 (m, 2H), 7.67 (td, *J* = 7.7, 1.8 Hz, 1H), 7.52–7.44 (m, 2H), 7.22 (dd, *J* = 7.6, 4.4 Hz, 2H), 3.99–3.89 (m, 2H), 3.19–3.11 (m, 2H). ^{13}C NMR (101 MHz, $CDCl_3$) δ 169.06, 167.97, 158.92, 152.79, 149.18, 137.93, 136.96, 129.33, 129.33, 128.86, 128.86, 124.36, 123.55, 121.96, 38.88, 35.78. HRMS (ESI): calcd for $C_{16}H_{14}ClN_4O_2$ ([M+H]$^+$), 329.07998; found, 329.07922.

Data for *N*-(2-(3-chloro-5-(trifluoromethyl)pyridin-2-yl)ethyl)-3-(4-chlorophenyl)-1,2,4-oxadiazole-5-
carboxamide (**F10**), white solid, m.p. 109–110 ℃, yield 43.7%, purity 99.5%; ^{1}H NMR (500 MHz, $CDCl_3$) δ 8.77 (s, 1H), 8.0 7 (s, 1H), 8.00 (d, *J* = 8.6 Hz, 2H), 7.92 (d, *J* = 1.8 Hz, 1H), 7.48–7.43 (m, 2H), 4.04 (dd, *J* = 9.6 Hz, *J* = 8.8 Hz, 2H), 3.33 (t, *J* = 4.8 Hz, 2H). ^{13}C NMR (126 MHz, $CDCl_3$) δ 168.88, 168.06, 160.26, 152.95, 143.84 (d, *J* = 3.0 Hz), 138.13, 134.11 (d, *J* = 2.1 Hz), 132.04, 129.46, 129.46, 128.93, 128.93, 126.33 (q, *J* = 54.3 Hz), 124.29, 122.73 (d, *J* = 272.9 Hz), 36.88, 33.98. HR MS (ESI): calcd for $C_{17}H_{12}C_{12}F_3N_4O_2$ ([M+H]$^+$), 431.02839; found, 431.02731.

Data for 3-(4-chlorophenyl)-*N*-(2,4-difluorobenzyl)-1,2,4-oxadiazole-5-carboxamide (**F11**), white solid, m.p. 97–98 ℃, yield 54.2%, purity 98.5%; ^{1}H NMR (400 MHz, $CDCl_3$) δ 8.02 (d, *J* = 8.4 Hz, 2H), 7.55 (s, 1H), 7.47 (d, *J* = 8.4 Hz, 2H), 7.43 (dd, *J* = 8.8, 6.4 Hz, 1H), 6.92–6.83 (m, 2H), 4.69 (d, *J* = 6.0 Hz, 2H). ^{13}C NMR (101 MHz, $CDCl_3$) δ 168.51, 167.99, 163.22 (d, *J* = 176.2 Hz), 160.80 (d, *J* = 185.5 Hz), 152.89, 138.16, 131.65 (dd, *J* = 9.1, 5.2 Hz), 129.41, 129.41, 128.86, 128.86, 124.12, 119.59 (d, *J* = 15.2 Hz), 111.75 (dd, *J* = 21.4, 3.7 Hz), 104.23, 37.56. HRMS (ESI): calcd for $C_{16}H_{10}ClF_2N_3O_2Na$ ([M+Na]$^+$), 372.03218; found, 372.03220.

Data for 3-(4-chlorophenyl)-N-(2,4-dichlorophenyl)-1,2,4-oxadiazole-5-carboxamide (**F12**), white solid, m.p. 169–171 ℃, yield 57.3%, purity 98.5%; ^{1}H NMR (400 MHz, $CDCl_3$) δ 9.41 (s, 1H), 8.48 (d, *J* = 9.2 Hz, 1H), 8.09 (d, *J* = 8.4 Hz, 2H), 7.51 (d, *J* = 8.8 Hz, 2H), 7.49 (d, *J* = 2.4 Hz, 1H), 7.35 (dd, *J* = 8.8, 2.0 Hz, 1H). ^{13}C NMR (101 MHz, $CDCl_3$) δ 168.43, 168.12, 150.26, 138.38, 131.79, 131.14, 131.14, 129.50, 129.50, 129.23, 128.96, 128.96, 128.35, 124.00, 122.28. HRMS (ESI): calcd for $C_{15}H_7Cl_3N_3O_2$ ([M-H]$^-$), 365.96984; found, 365.96996.

Data for *N*-(2-(pyridin-2-yl)ethyl)-3-(thiophen-2-yl)-1,2,4-oxadiazole-5-carboxamide (**F13**), yellow solid, m.p. 133–135 ℃, yield 57.2%, purity 98.7%; ^{1}H NMR (500 MHz, $CDCl_3$) δ 8.62 (s, 1H), 8.58 (d, *J* = 4.5 Hz, 1H), 7.82 (d, *J* = 4.5 Hz, 1H), 7.64 (t, *J* = 17.5 Hz, 1H), 7.53 (d, *J* = 6.0 Hz, 1H), 7.19 (t, *J* = 2.5 Hz, 2H), 7.15 (dd, *J* = 5.0, 4.0 Hz, 1H), 3.91 (dd, *J* = 12.5, 11.0Hz, 2H), 3.14–3.09 (m, 2H). ^{13}C NMR (126

MHz, $CDCl_3$) δ 168.82, 164.91, 158.99, 152.82, 149.32, 137.01, 130.48, 130.16, 128.22, 127.33, 123.62, 122.03, 38.99, 35.90. HRMS (ESI): calcd for $C_{14}H_{13}N_4O_2S$ ([M+H]$^+$), 310.07537; found, 310.07428.

Data for *N*-(2-(3-chloro-5-(trifluoromethyl)pyridin-2-yl)ethyl)-3-(thiophen-2-yl)-1,2,4-oxadiazole-5-carboxamide (**F14**), yellow solid, m.p. 94–96 ℃, yield 56.0%, purity 99.4%; ^{1}H NMR (500 MHz, $CDCl_3$) δ 8.76 (s, 1H), 8.07 (s, 1H), 7.92 (s, 1H), 7.80 (d, *J* = 3.5 Hz, 1H), 7.53 (d, *J* = 6.0 Hz, 1H), 7.15 (dd, *J* = 5.0, 3.8 Hz, 1H), 4.03 (dd, *J* = 12.0, 6.2 Hz, 2H), 3.32 (t, *J* = 5.9 Hz, 2H). ^{13}C NMR (126 MHz, $CDCl_3$) δ 168.55, 164.88, 160.24, 152.89, 143.84 (d, *J* = 3.9 Hz), 134.09 (d, *J* = 3.3 Hz), 132.01, 130.52, 130.26, 128.26, 127.14, 126.30 (d, *J* = 33.9 Hz), 122.74 (d, *J* = 272.9 Hz), 36.91, 33.96. HRMS (ESI): calcd for $C_{15}H_{11}ClF_3N_4O_2S$ ([M+H]$^+$), 403.02379; found, 403.02222.

Data for *N*-(2,4-difluorobenzyl)-3-(thiophen-2-yl)-1,2,4-oxadiazole-5-carboxamide (**F15**), yellow solid, m.p. 102–103 ℃, yield 63.7%, purity 98.8%; ^{1}H NMR (400 MHz, $CDCl_3$) δ 7.82 (dd, *J* = 3.6, 0.8 Hz, 1H), 7.55 (dd, *J* = 4.8, 1.2 Hz, 2H), 7.47–7.39 (m, 1H), 7.17 (dd, *J* = 4.8, 3.6 Hz, 1H), 6.92–6.82 (m, 2H), 4.68 (d, *J* = 6.0 Hz, 2H). ^{13}C NMR (101 MHz, $CDCl_3$) δ 168.17, 164.79, 163.26 (d, *J* = 164.9 Hz), 160.72 (d, *J* = 176.4 Hz), 152.83, 131.64 (dd, *J* = 9.8, 5.6 Hz), 130.46, 130.26, 128.22, 126.96, 119.56 (d, J = 15.2 Hz), 111.74 (d, J = 25.3 Hz), 104.22, 37.48. HRMS (ESI): calcd for $C_{14}H_{10}F_2N_3O_2S$ ([M+H]$^+$), 322.04563; found, 322.04425.

Data for *N*-(2,4-dichlorophenyl)-3-(thiophen-2-yl)-1,2,4-oxadiazole-5-carboxamide (**F16**), white solid, m.p. 147–148 ℃, yield 53.7%, purity 99.3%; ^{1}H NMR (400 MHz, $CDCl_3$) δ 9.38 (s, 1H), 8.46 (d, *J* = 9.2 Hz, 1H), 7.90 (dd, *J* = 4.0, 1.2 Hz, 1H), 7.59 (dd, *J* = 5.2, 1.2 Hz, 1H), 7.49 (d, *J* = 2.4 Hz, 1H), 7.35 (dd, *J* = 8.8, 2.4Hz, 1H), 7.20 (dd, *J* = 4.8, 3.6 Hz, 1H). ^{13}C NMR (101 MHz, $CDCl_3$) δ 168.06, 164.97, 150.23, 131.80, 131.12, 130.85, 130.53, 129.23, 128.31, 128.24, 126.70, 124.19, 122.35. HRMS (ESI): calcd for $C_{13}H_7Cl_2N_3O_2SNa$ ([M+Na]$^+$), 361;95282; found, 361;95276.

Data for 3-(6-bromopyridin-3-yl)-*N*-(2-(pyridin-2-yl)ethyl)-1,2,4-oxadiazole-5-carboxamide (**F17**), white solid, m.p. 135–137 ℃, yield 53.0%, purity 93.8%; ^{1}H NMR (400 MHz, $CDCl_3$) δ 9.15–9.12 (m, 1H), 8.80 (s, 1H), 8.60 (d, *J* = 4.4 Hz, 1H), 8.36 (dd, *J* = 8.4, 2.4 Hz, 1H), 7.67 (td, *J* = 7.6, 1.6 Hz, 1H), 7.50 (dd, *J* = 8.4, 0.4 Hz, 1H), 7.25–7.19 (m, 2H), 3.99–3.91 (m, 2H), 3.19–3.11 (m, 2H). ^{13}C NMR (101 MHz, $CDCl_3$) δ 169.56, 166.09, 158.97, 154.58, 152.46, 149.20, 148.79, 137.29, 137.00, 124.75, 123.57, 122.01, 121.26, 38.94, 35.68. HRMS (ESI): calcd for $C_{15}H_{12}BrN_5O_2$ ([M-H]$^-$), 407.98574; found, 407.98596.

Data for 3-(6-bromopyridin-3-yl)-*N*-(2-(3-chloro-5-(trifluoromethyl)pyridin-2-yl)ethyl)-1,2,4-oxadiazole-5-carboxamide (**F18**), white solid, m.p. 118–120 ℃, yield 43.8%, purity 99.6%; ^{1}H NMR (400 MHz, $CDCl_3$) δ 9.10 (d, *J* = 2.4 Hz, 1H), 8.78 (s, 1H), 8.34 (dd, *J* = 8.2, 2.4 Hz, 1H), 8.13 (s, 1H), 7.95 (d, *J* = 1.6 Hz, 1H), 7.50 (d, *J* = 8.2 Hz, 1H), 4.07 (dd, *J* = 12.0, 6.0 Hz, 2H), 3.36 (t, *J* = 6.0 Hz, 2H). ^{13}C NMR (101 MHz, $CDCl_3$) δ 169.25, 166.11, 160.14, 154.69, 152.54, 148.75, 143.76 (d, *J* = 3.9 Hz), 137.27, 134.05, 131.97, 126.51, 126.18, 124.78, 121.11, 36.92, 33.84. HRMS (ESI): calcd for $C_{16}H_{10}BrClF_3N_5O_2$ ([M-H]$^-$), 509.93415; found, 509.93390.

Data for 3-(6-bromopyridin-3-yl)-*N*-(2,4-difluorobenzyl)-1,2,4-oxadiazole-5-carboxamide (**F19**), white solid, m.p. 128–129 ℃, yield 53.1%, purity 98.5%; ^{1}H NMR (400 MHz, $CDCl_3$) δ 9.08 (d, *J* = 2.0 Hz, 1H), 8.34 (dd, *J* = 8.2, 2.4 Hz, 1H), 7.60 (s, 1H), 7.49 (d, *J* = 8.0 Hz, 1H), 7.48–7.40 (m, 1H), 7.01–6.78 (m, 2H), 4.71 (d, *J* = 6.0 Hz, 2H). ^{113}C NMR (101 MHz, $CDCl_3$) δ 168.94, 166.11, 163.30 (dd, *J* = 178.1, 11.9 Hz), 160.81 (dd, *J* = 177.8, 12.2 Hz), 154.76, 152.59, 148.71, 137.28, 131.68 (dd, *J* = 9.8, 6.4 Hz), 124.82, 121.01, 119.47 (dd, *J* = 14.9, 3.7 Hz), 111.77 (dd, *J* = 21.5, 3.9 Hz), 104.26, 37.62. HRMS (ESI): calcd for $C_{15}H_9BrF_2N_4O_2$ ([M-H]$^-$), 428.95600; found, 428.95587.

Data for 3-(6-bromopyridin-3-yl)-*N*-(2,4-dichlorophenyl)-1,2,4-oxadiazole-5-carboxamide (**F20**), white solid, m.p. 201–202 ℃, yield 53.8%, purity 99.0%; ^{1}H NMR (400 MHz, DMSO-*d6*) δ 11.18 (s, 1H), 9.10 (d, *J* = 2.2 Hz, 1H), 8.50 (dd, *J* = 8.4, 2.4 Hz, 1H), 7.82 (d, *J* = 6.1 Hz, 1H), 7.81 (s, 1H), 7.70 (d, *J* = 8.6 Hz, 1H), 7.55 (dd, *J* = 8.6, 2.4 Hz, 1H). ^{13}C NMR (101 MHz, DMSO-*d6*) δ 169.65, 166.28, 153.88, 152.01, 148.84, 138.74, 132.73, 132.53, 130.78, 129.86, 129.51, 128.52, 125.80, 121.82. HRMS (ESI): calcd for $C_{14}H_6BrCl_2N_4O_2$ ([M-H]$^-$), 410.90457; found, 410.90454

Data for 3-(3-chloro-5-(trifluoromethyl)pyridin-2-yl)-*N*-(2-(pyridin-2-yl)ethyl)-1,2,4-oxadiazole-5-carboxamide (**F21**), yellow solid, m.p. 140–141 ℃, yield 56.5%, purity 92.4%; ^{1}H NMR (400 MHz, $CDCl_3$) δ 8.96 (d, *J* = 1.22 Hz, 1H), 8.56 (dd, *J* = 4.8, 0.8 Hz, 1H), 8.87 (s, 1H), 8.18 (d, *J* = 2.4 Hz, 1H), 7.46 (td, *J* = 8.0, 2.0 Hz, 1H), 7.21–7.17 (m, 1H), 3.96 (dd, *J* = 12.4, 7.0 Hz, 2H), 3.14 (t, *J* = 6.4 Hz, 2H). ^{13}C NMR (101 MHz, $CDCl_3$) δ 169.37, 166.17, 158.73, 152.42, 149.33, 146.60, 144.64 (d, *J* = 3.8 Hz), 136.81, 136.32 (d, *J* = 3.5 Hz), 132.70, 129.01 (d, *J* = 34.2 Hz), 123.47, 121.88, 120.75, 39.00, 35.99. HRMS (ESI): calcd for $C_{16}H_{12}ClF_3N_5O_2$ ([M+H]$^+$), 398.06261; found, 398.06070.

Data for 3-(3-chloro-5-(trifluoromethyl)pyridin-2-yl)-*N*-(2-(3-chloro-5-(trifluoromethyl)pyridin-2-yl) ethyl)-1,2,4-oxadiazole-5-carboxamide (**F22**), white solid, m.p. 158–159 ℃, yield 49.9%, purity 99.4%; ^{1}H NMR (400 MHz, $CDCl_3$) δ 8.96 (d, *J* = 0.8 Hz, 1H), 8.74 (s, 1H), 8.19 (d, *J* = 1.2 Hz, 1H), 8.08 (s, 1H), 7.93 (d, *J* = 1.6 Hz, 1H), 4.07 (m, 2H), 3.35 (m, 2H). ^{13}C NMR (101 MHz, $CDCl_3$) δ 169.13, 166.09, 159.98, 152.48, 146.44, 144.66 (d, *J* = 3.8 Hz), 143.82 (d, *J* = 4.0 Hz), 136.39 (d, *J* = 3.5 Hz), 133.97 (d, *J* = 3.6 Hz), 132.75, 131.91, 129.09 (d, *J* = 34.3 Hz), 126.23 (d, *J* = 33.7 Hz), 123.72 (d, *J* = 57.3 Hz), 121.00 (d, *J* = 57.8 Hz), 36.90, 33.89. HRMS (ESI): calcd for $C_{17}H_{10}Cl_2F_6N_5O_2$ ([M+H]$^+$), 500.01103; found, 500.00858.

Data for 3-(3-chloro-5-(trifluoromethyl)pyridin-2-yl)-*N*-(2,4-difluorobenzyl)-1,2,4-oxadiazole-5-carboxamide (**F23**), white solid, m.p. 112–114 ℃, yield 47.7%, purity 98.6%; ^{1}H NMR (400 MHz, $CDCl_3$) δ 8.95 (s, 1H), 8.19 (s, 1H), 7.64 (s, 1H), 7.46–7.37 (m, 1H), 6.92–6.81 (m, 2H), 4.68 (d, *J* = 6.0 Hz, 2H). ^{13}C NMR (101 MHz, $CDCl_3$) δ 168.82, 166.08, 164.13, 161.64, 152.41,

146.19, 144.67 (d, *J* = 3.8 Hz), 136.53 (d, *J* = 3.6 Hz), 132.82, 131.79 (dd, *J* = 9.9, 5.5 Hz), 129.12 (d, *J* = 34.3 Hz), 122.04 (d, *J* = 274.6 Hz), 119.27, 111.73 (dd, *J* = 21.4, 3.6 Hz), 104.22, 37.57. HRMS (ESI): calcd for $C_{16}H_9ClF_5N_4O_2$ ($[M+H]^+$), 419.03287; found, 419.03104.

Data for 3-(3-chloro-5-(trifluoromethyl)pyridin-2-yl)-*N*-(2,4-dichlorophenyl)-1,2,4-oxadiazole-5-carboxamide (**F24**), white solid, m.p. 137–138 ℃, yield 55.0%, purity 92.3%; ^1H NMR (400 MHz, $CDCl_3$) δ 9.41 (s, 1H), 8.99 (d, *J* = 0.8 Hz, 1H), 8.44 (d, *J* = 8.8 Hz, 1H), 8.21 (d, *J* = 1.2 Hz, 1H), 7.49 (d, *J* = 2.0 Hz, 1H), 7.37 (dd, *J* = 8.8, 2.0 Hz, 1H). ^{13}C NMR (101 MHz, $CDCl_3$) δ 168.72, 166.30, 149.94, 146.29, 144.76 (d, *J* = 3.7 Hz), 136.32 (d, *J* = 3.5 Hz), 132.84, 131.59, 131.41, 129.31, 128.31, 124.47, 123.45, 122.62, 120.73. HRMS (ESI): calcd for $C_{15}H_6Cl_3F_3N_4O_2Na$ ($[M+Na]^+$), 458.94006; found, 458.93991.

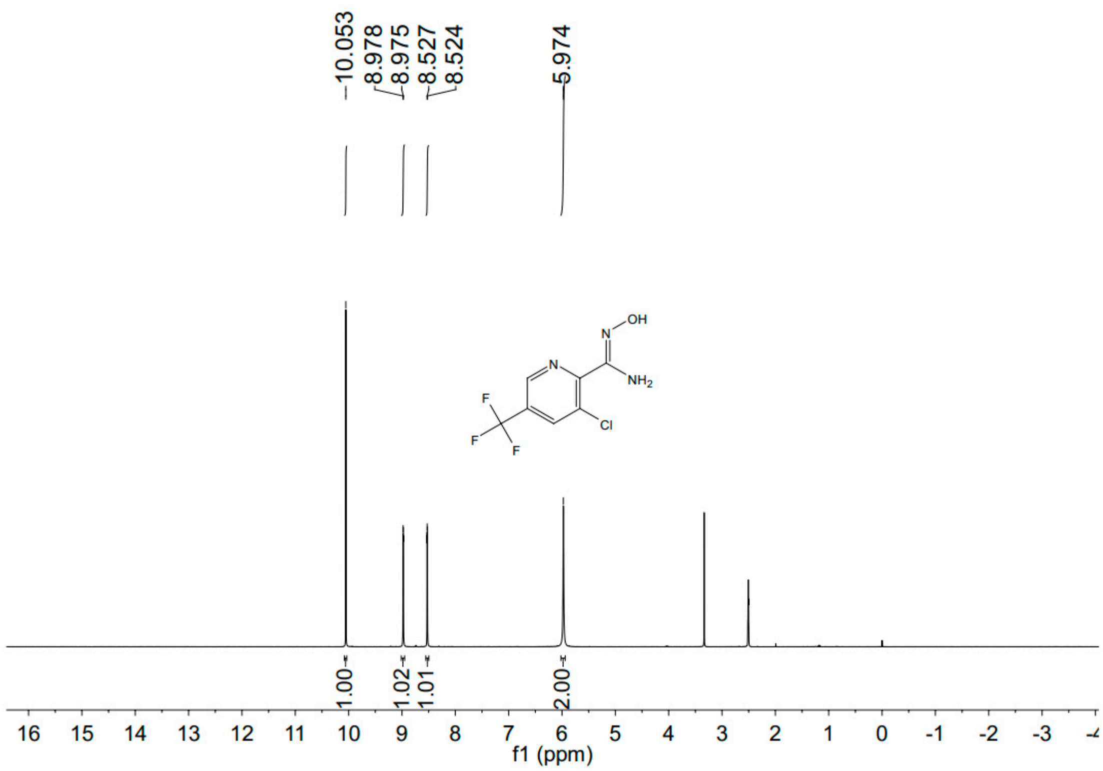

^{1}H NMR of intermediate **a6**

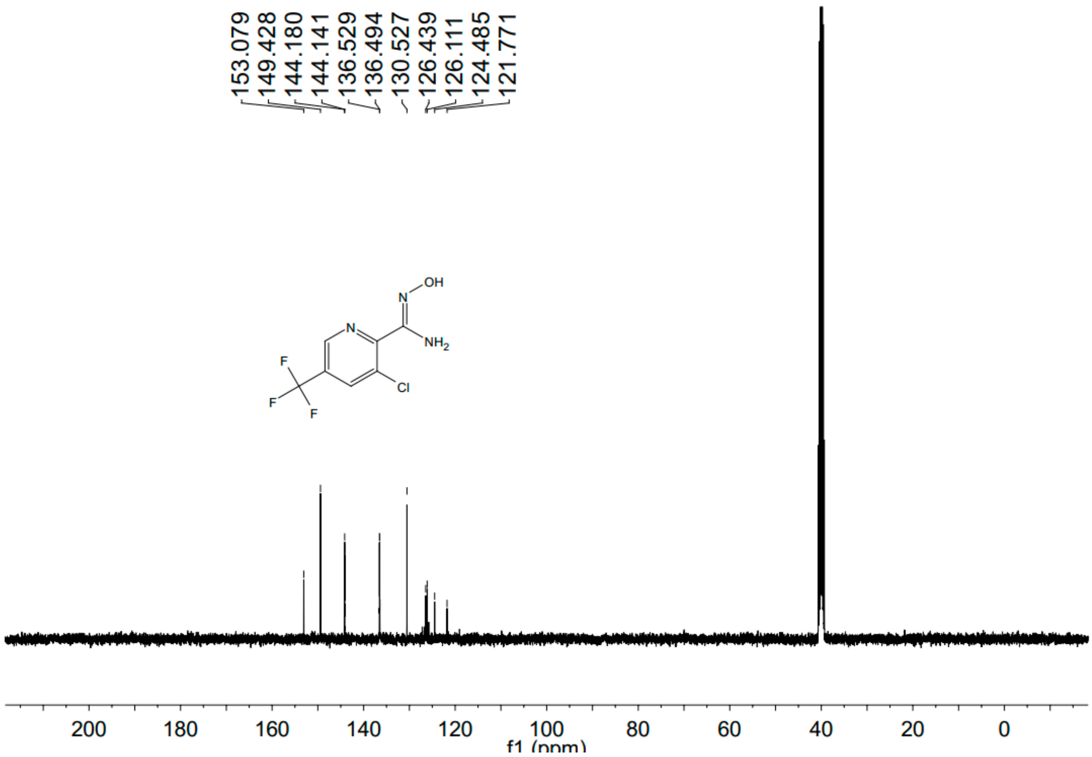

^{13}C NMR of intermediate **a6**

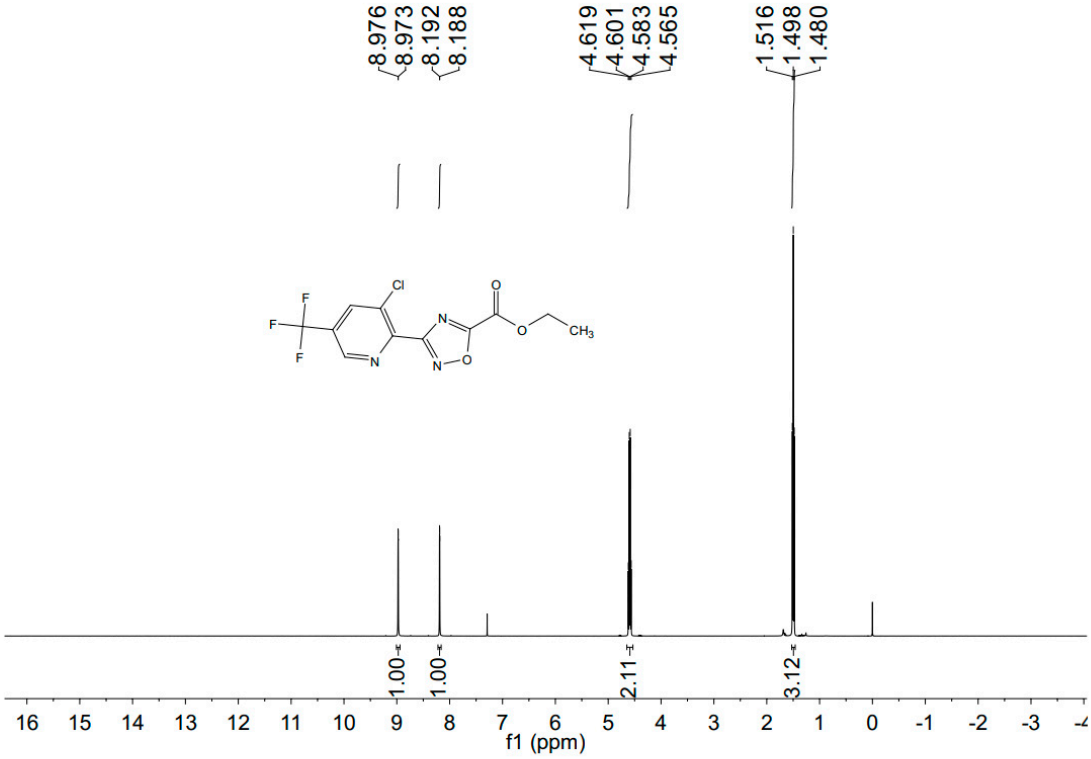

^{1}H NMR of intermediate **b6**

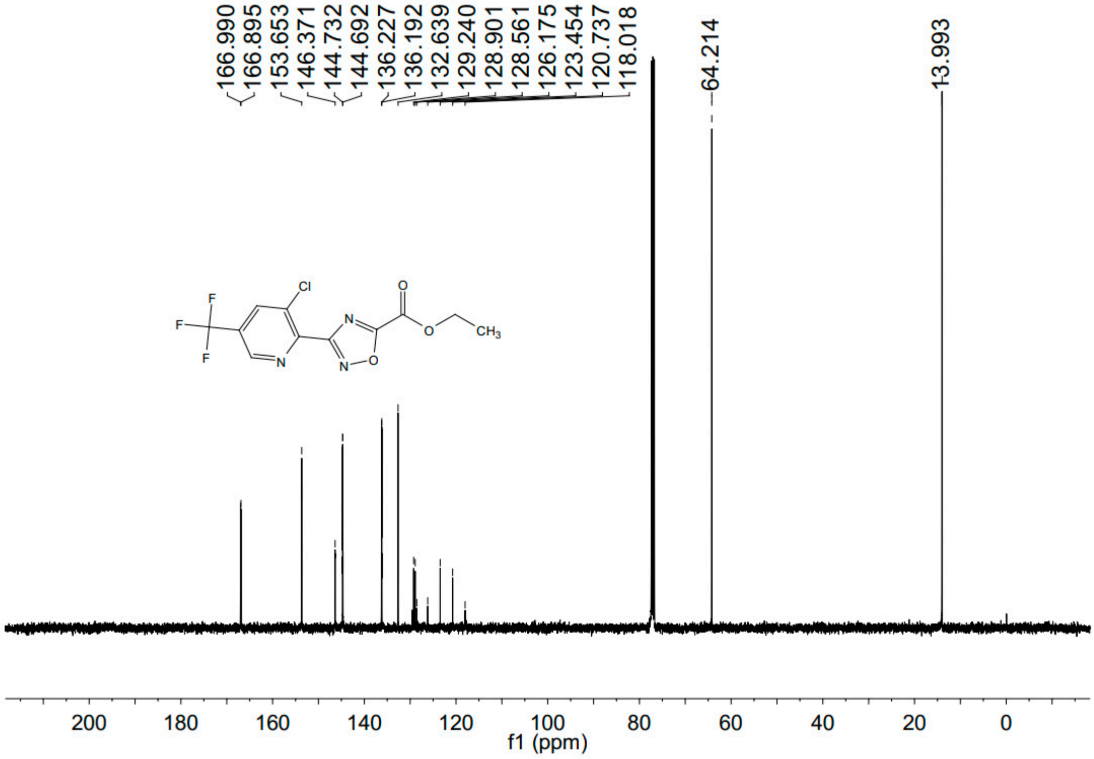

^{13}C NMR of intermediate **b6**

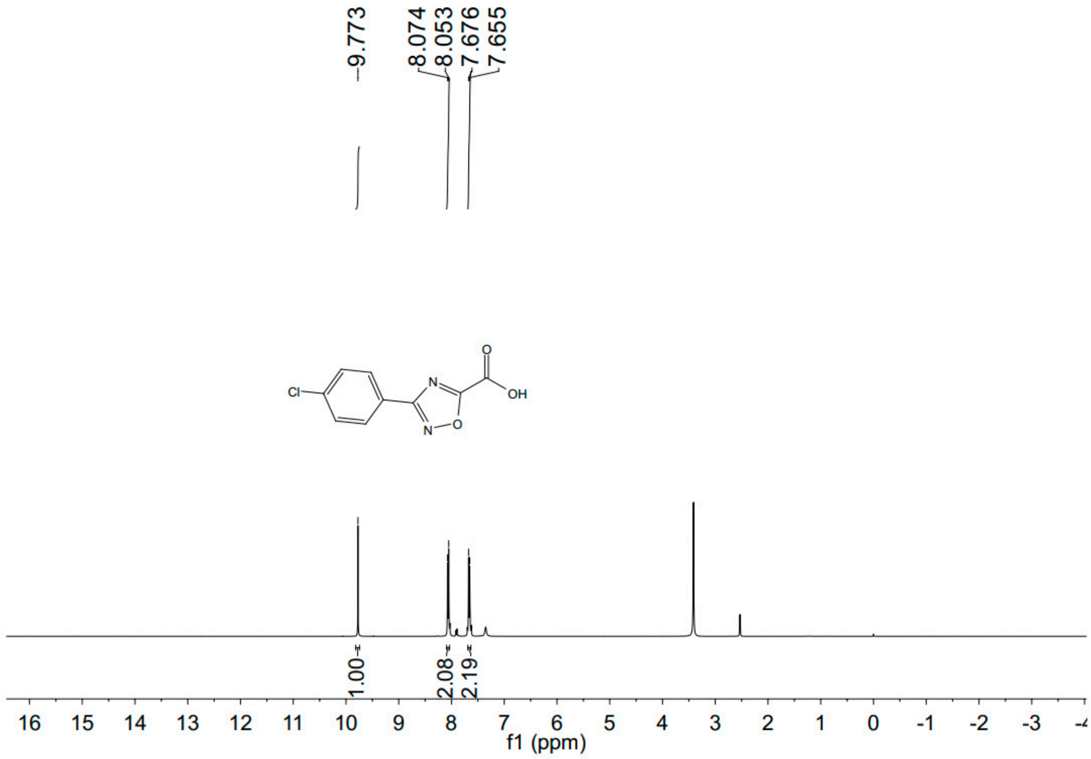

^{1}H NMR of intermediate **c3**

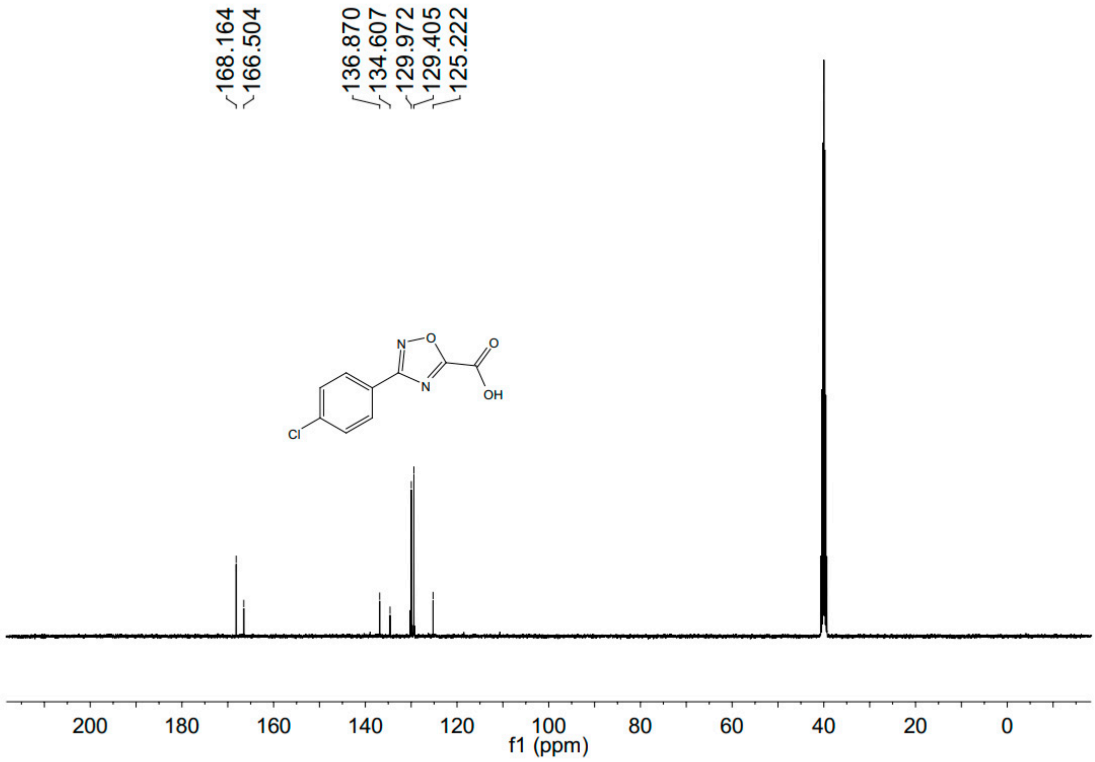

^{13}C NMR of intermediate **c3**

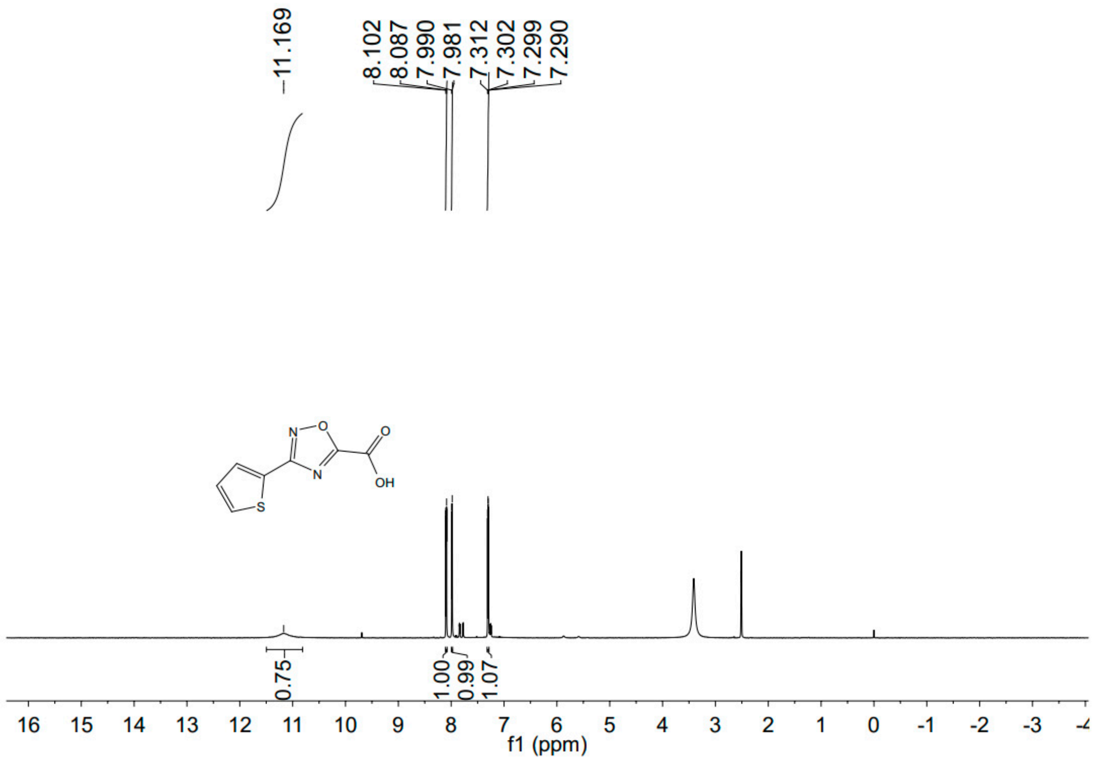

^{1}H NMR of intermediate **c4**

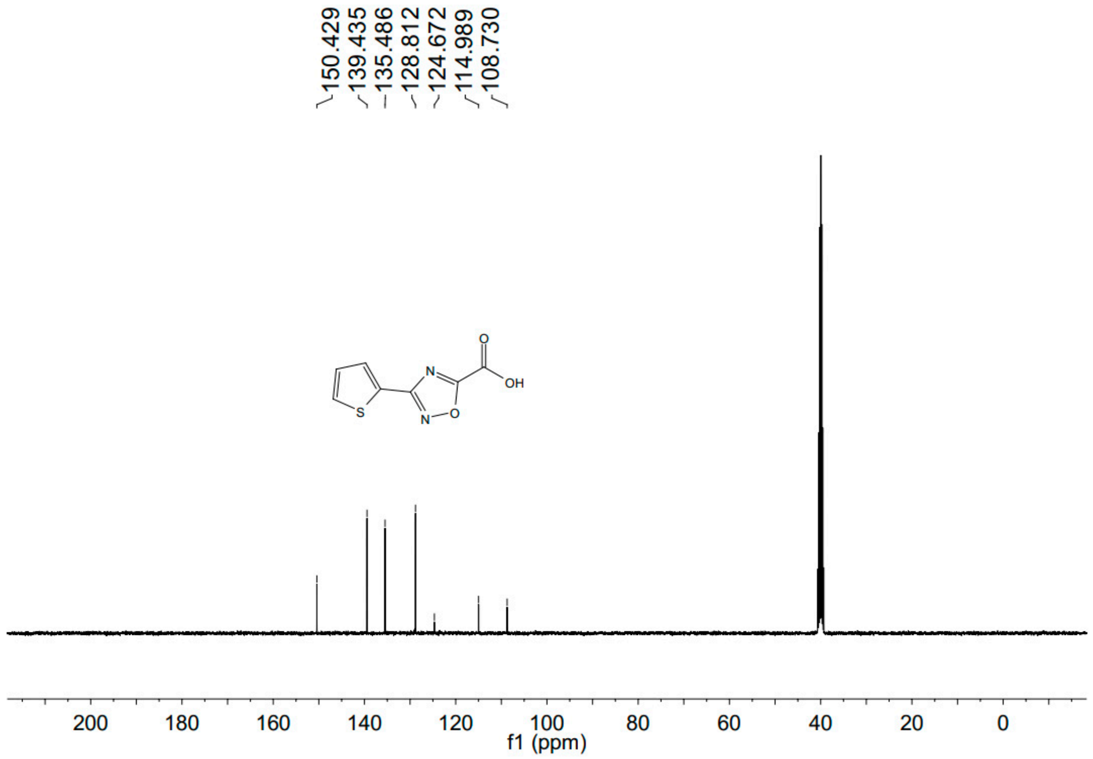

^{13}C NMR of intermediate **c4**

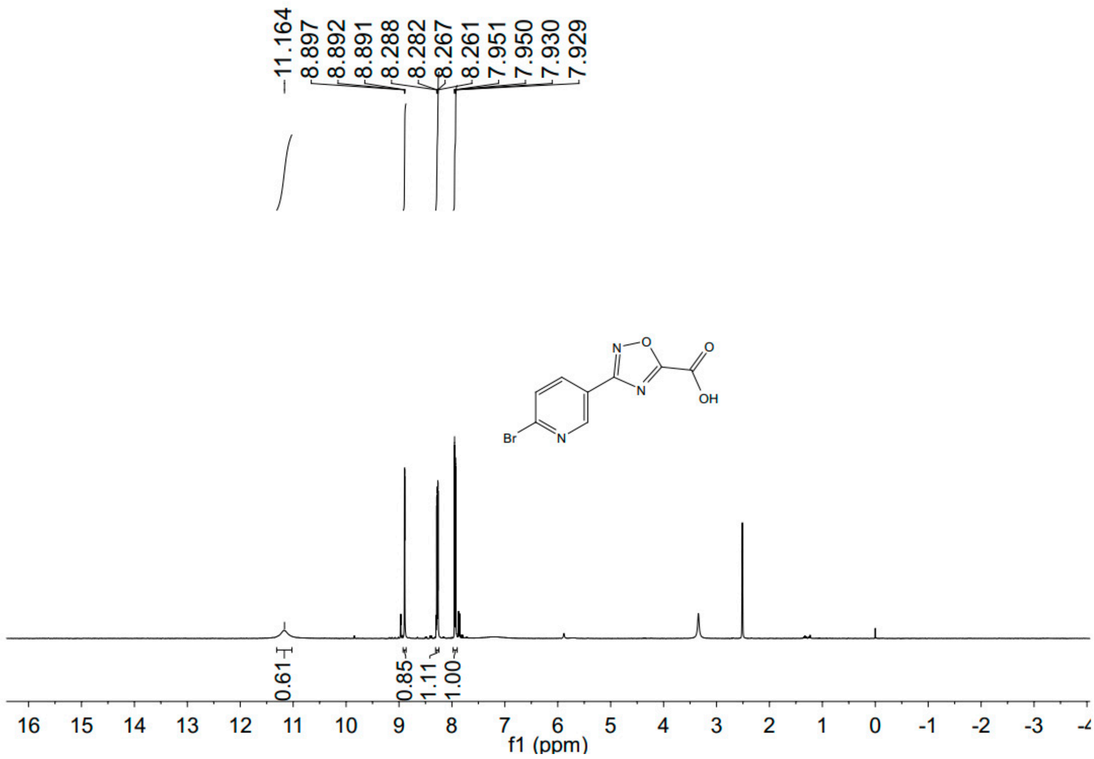

^{1}H NMR of intermediate **c5**

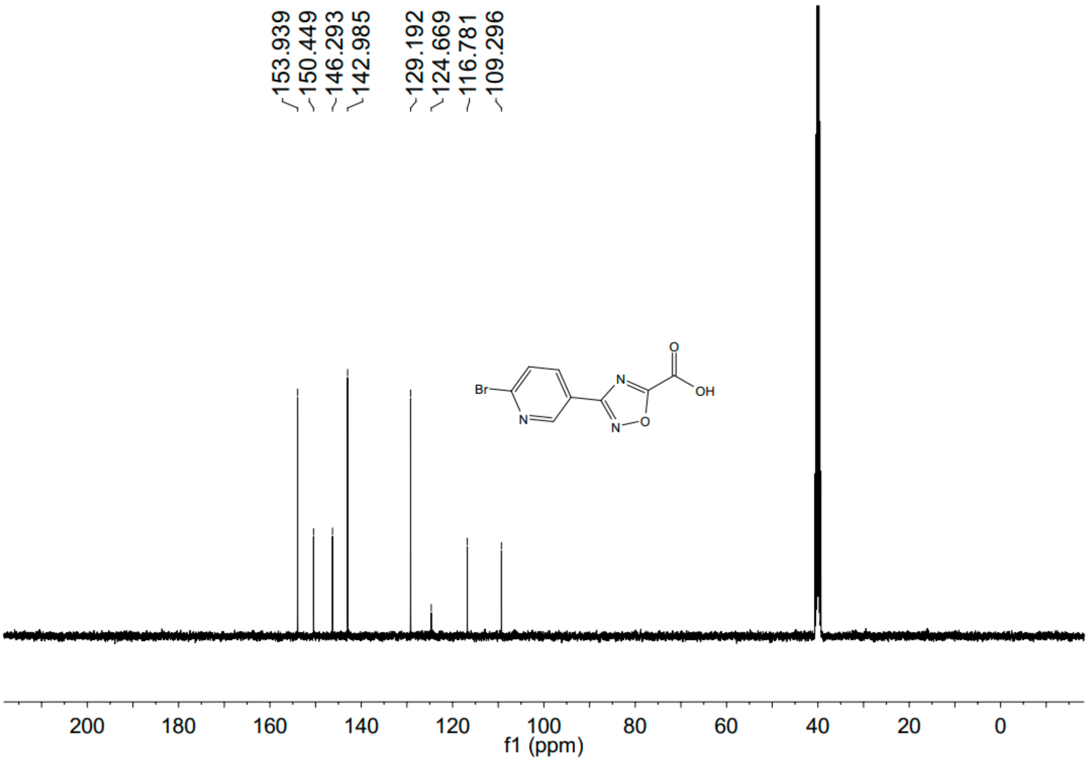

^{13}C NMR of intermediate **c5**

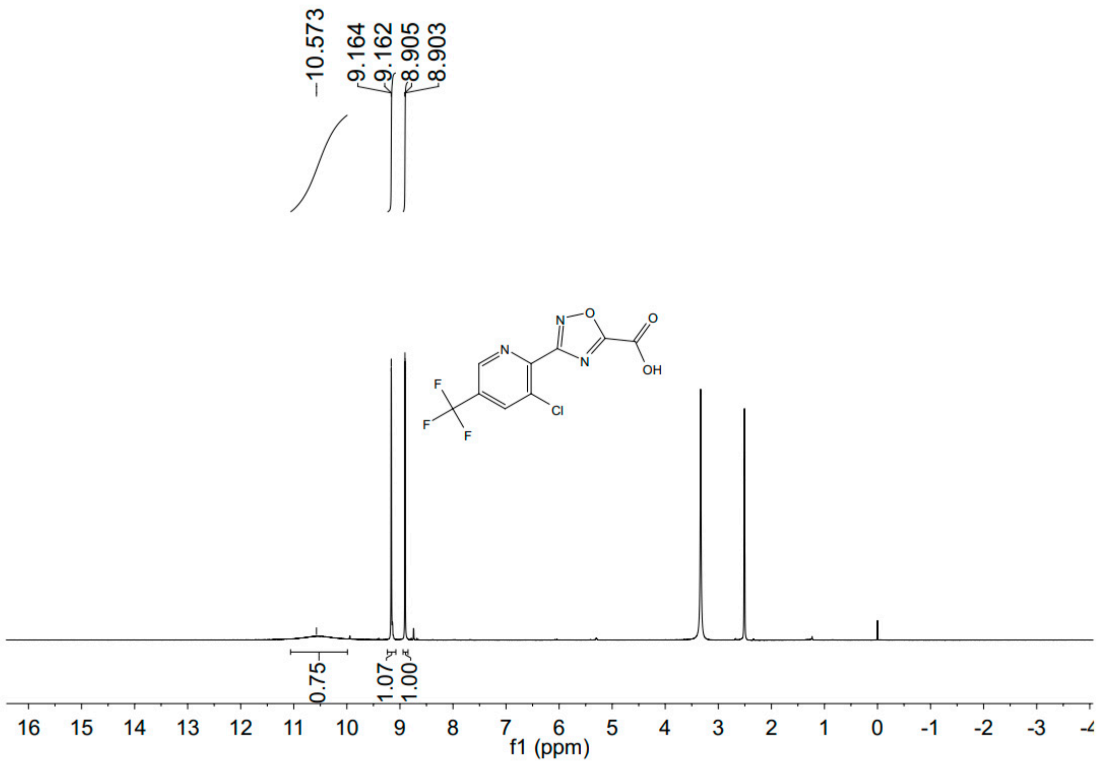

^{1}H NMR of intermediate **c6**

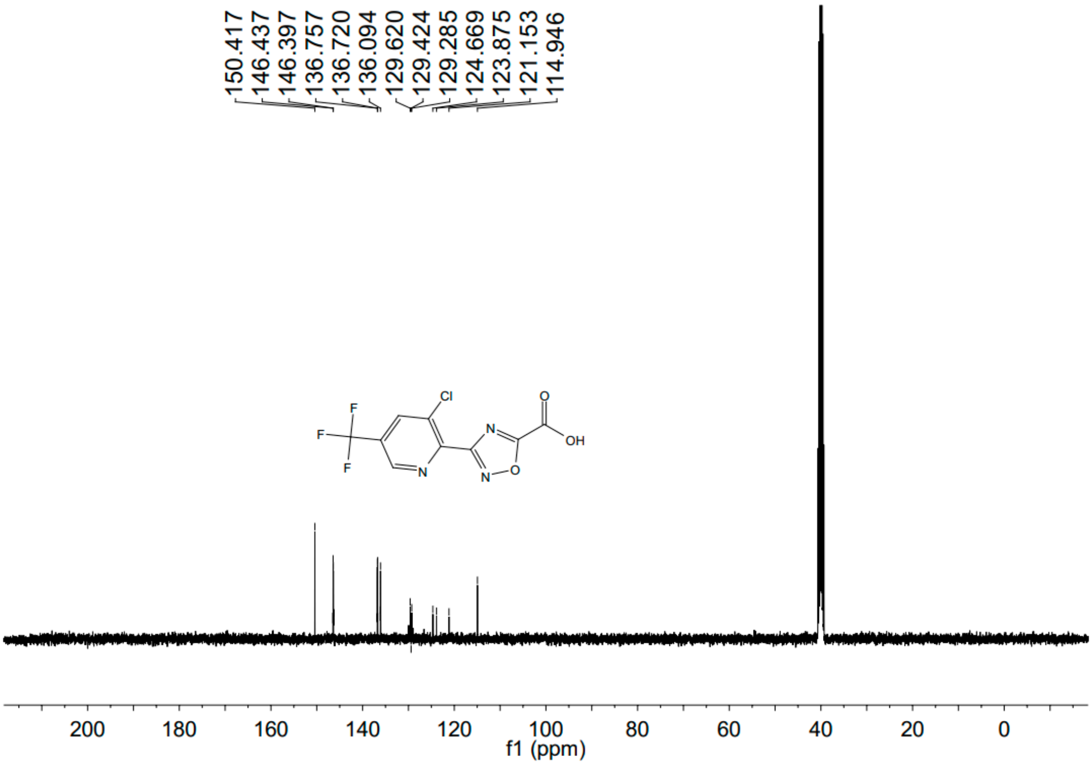

^{13}C NMR of intermediate **c6**

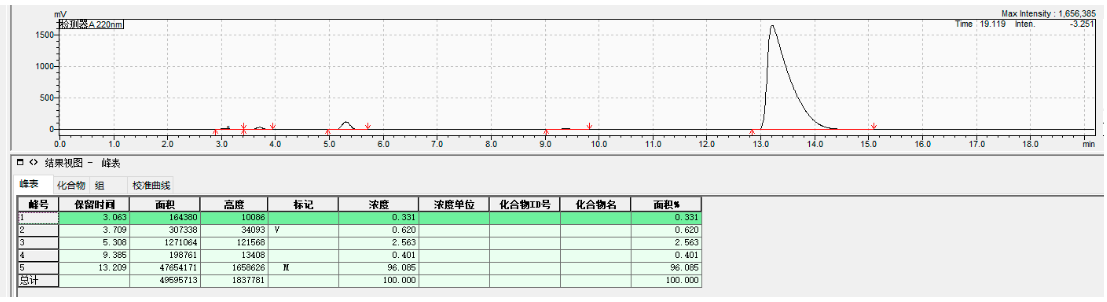

峰号	保留时间	面积	高度	标记	浓度	浓度单位	化合物ID号	化合物名	面积%
1	3. 063	164380	10086		0. 331				0. 331
2	3. 709	307338	34093	V	0. 620				0. 620
3	5. 308	1271064	121568		2. 563				2. 563
4	9. 385	198761	13408		0. 401				0. 401
5	13. 209	47654171	1658626	M	96. 085				96. 085
总计		49595713	1837781		100. 000				100. 000

HPLC of compound **F1**

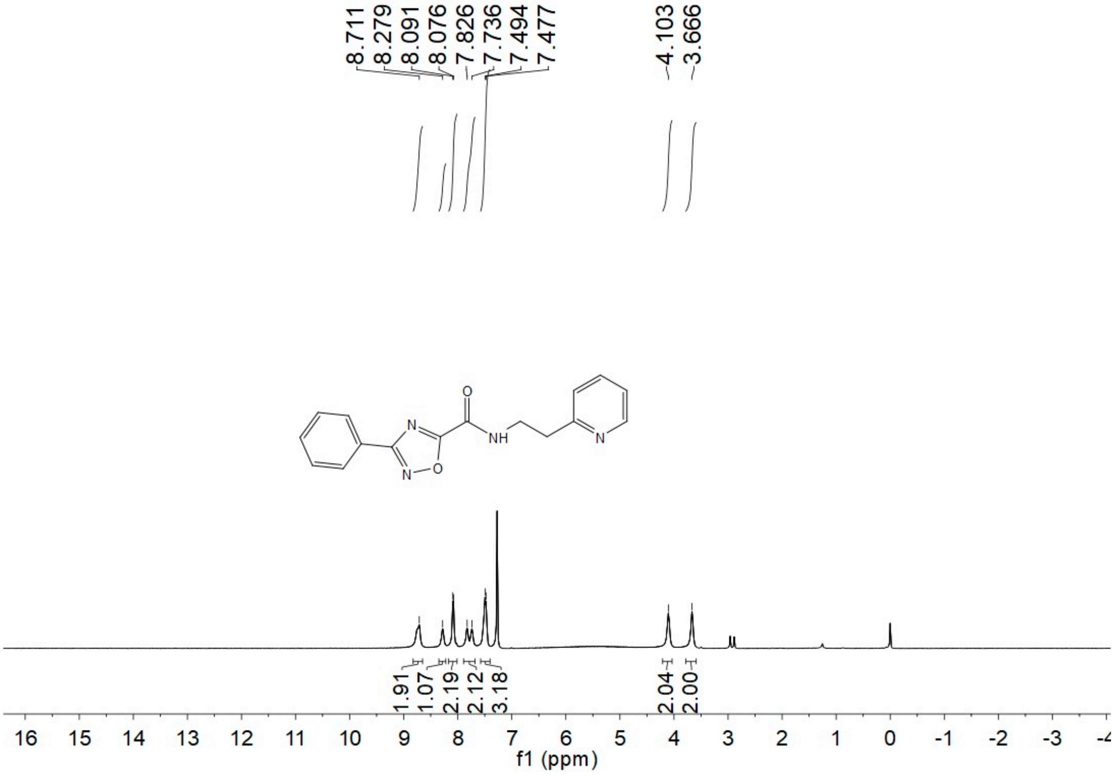

^{1}H NMR of compound **F1**

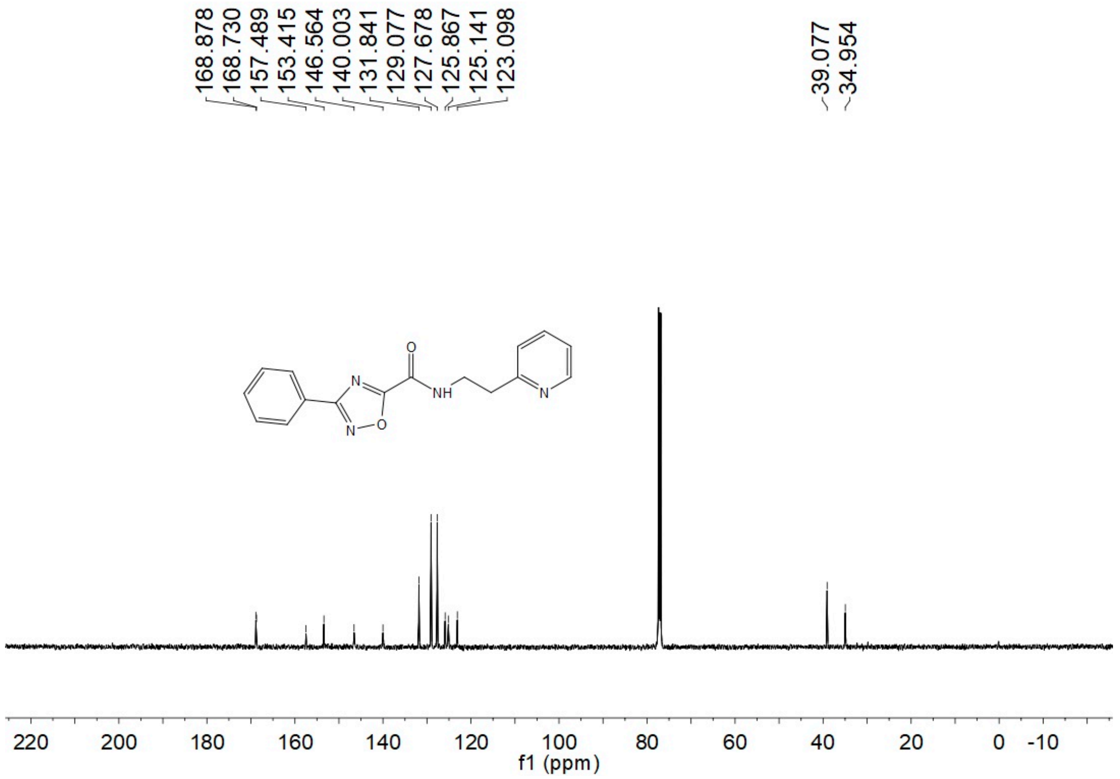

^{13}C NMR of compound **F1**

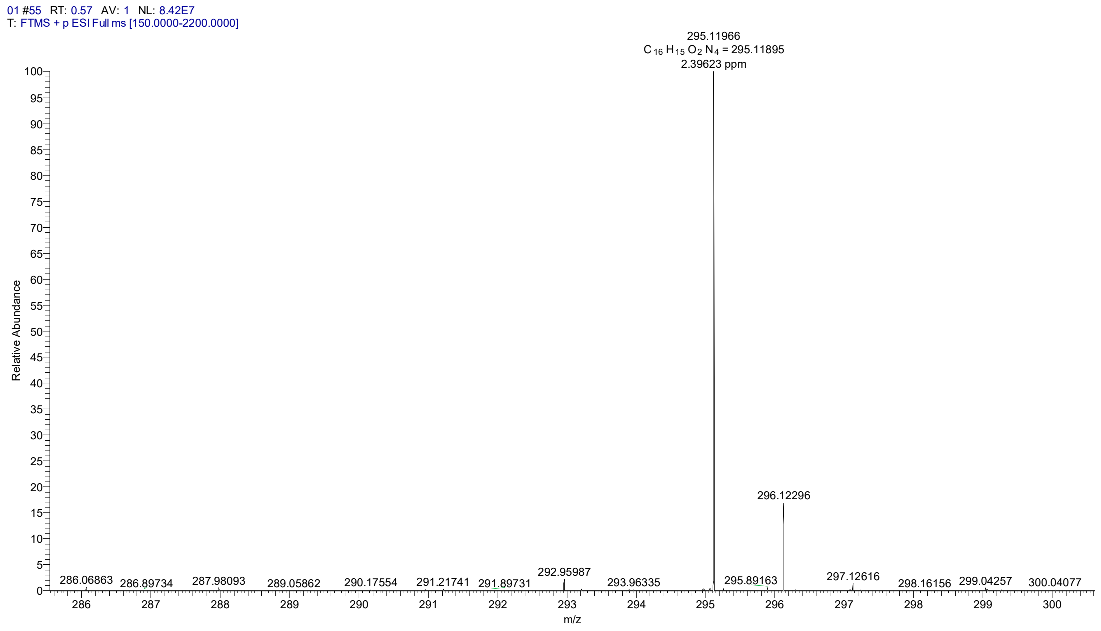

HRMS of compound **F1**

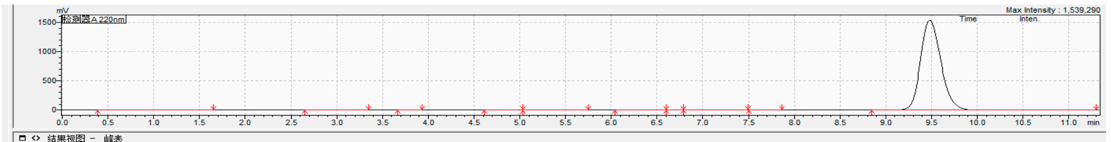

峰号	保留时间	面积	高度	标记	浓度	浓度单位	化合物ID号	化合物名	面积%
5	5.238	39691	4984	V	0.166				0.166
6	6.387	10281	641		0.043				0.043
7	6.692	1322	125	V	0.006				0.006
8	7.066	46219	3182	V	0.193				0.193
9	7.706	3163	237		0.013				0.013
10	9.481	23693183	1539843		98.989				98.989
总计		23935176	1560214		100.000				100.000

HPLC of compound **F2**

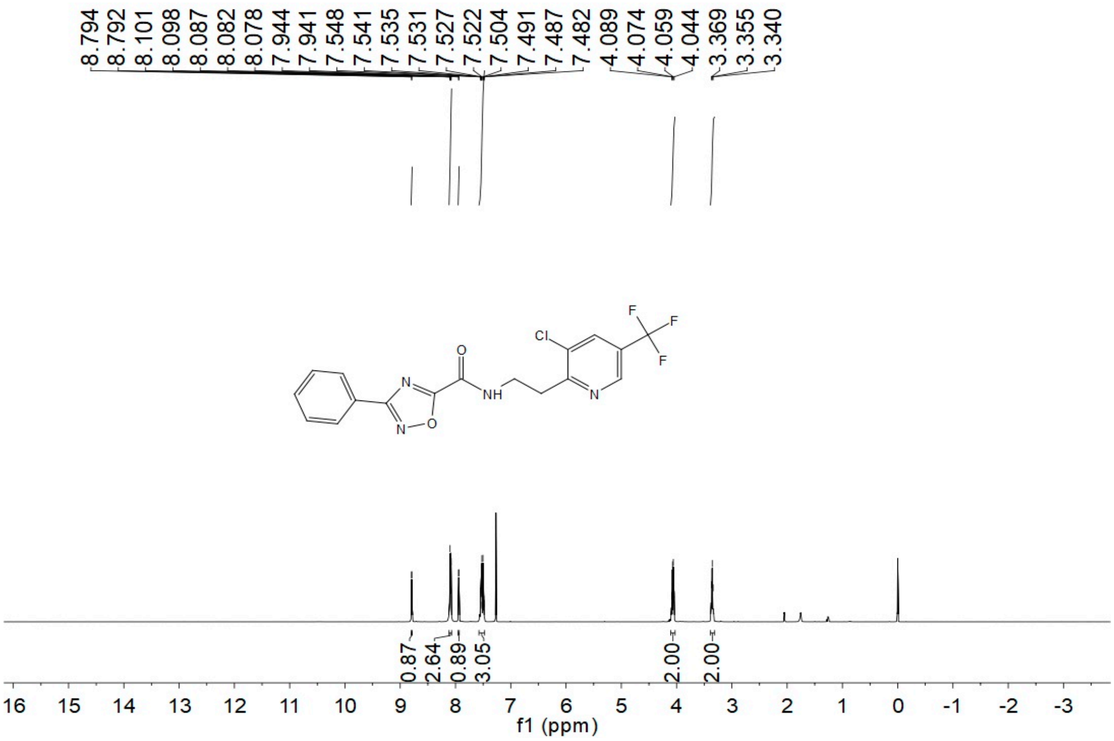

^{1}H NMR of compound **F2**

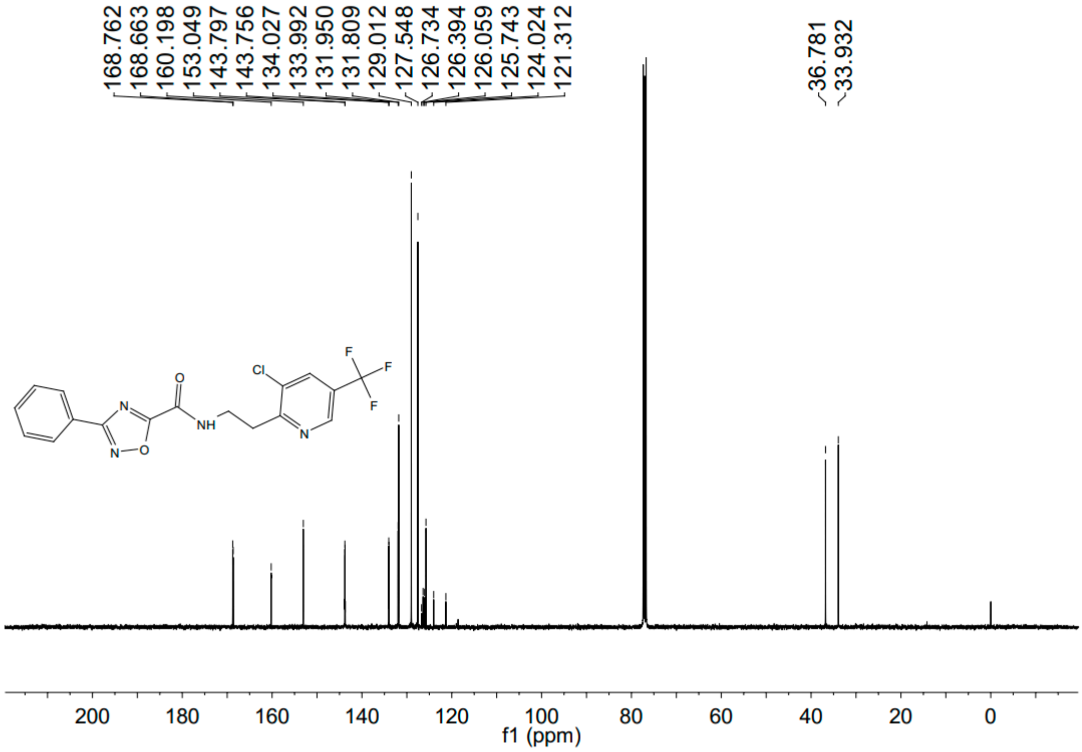

^{13}C NMR of compound **F2**

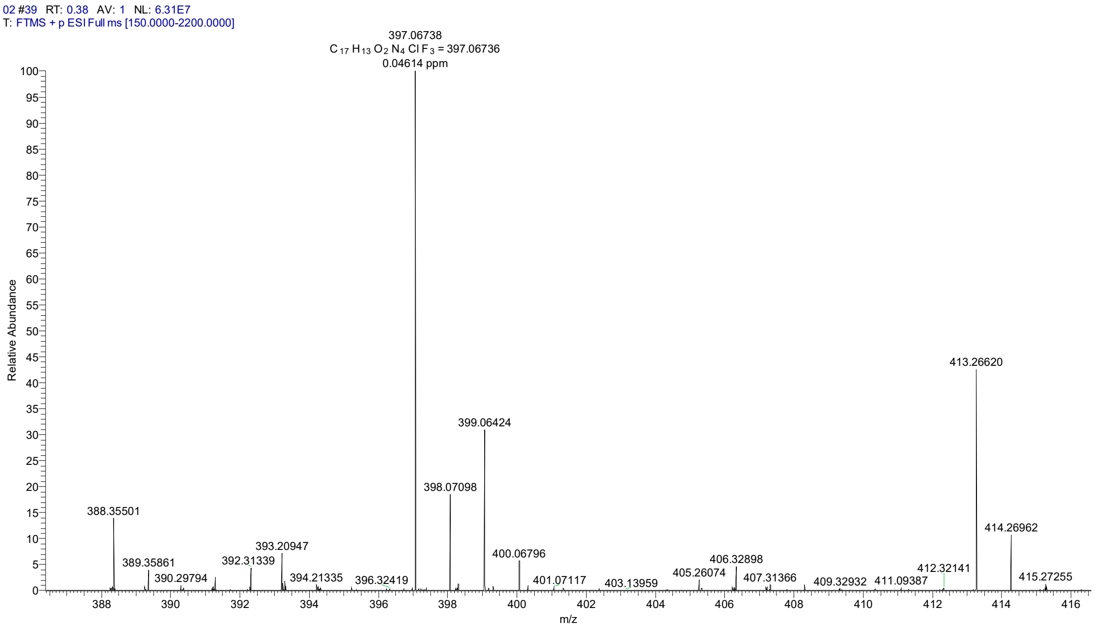

HRMS of compound **F2**

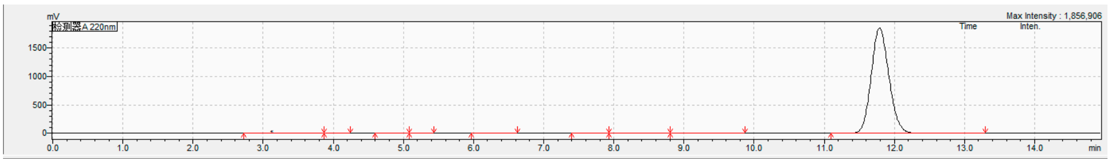

结果视图 − 峰表

峰表 化合物 组 校准曲线

峰号	保留时间	面积	高度	标记	浓度	浓度单位	化合物ID号	化合物名	面积%
4	5. 233	3819	498		0. 012				0. 012
5	6. 369	10045	637		0. 031				0. 031
6	7. 610	95354	9315	V	0. 290				0. 290
7	8. 324	44036	2396		0. 134				0. 134
8	9. 123	120529	7745		0. 366				0. 366
9	11. 789	32495337	1857767		98. 749				98. 749
总计		32906969	1887568		100. 000				100. 000

HPLC of compound **F3**

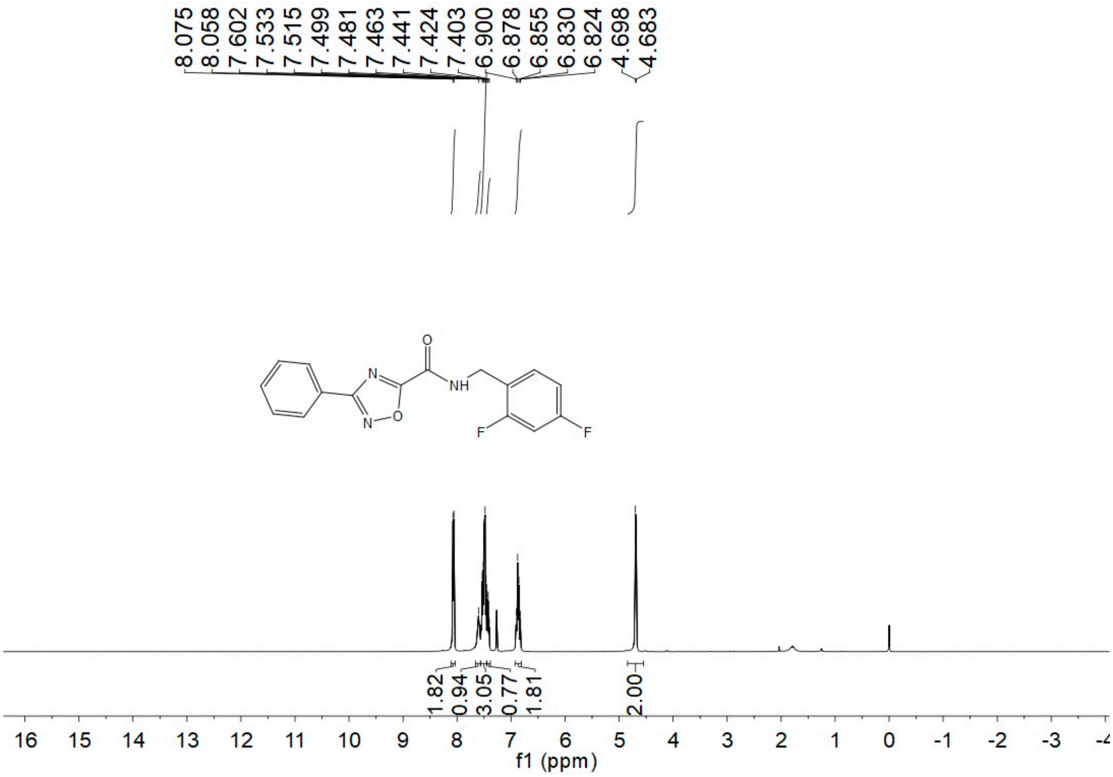

^{1}H NMR of compound **F3**

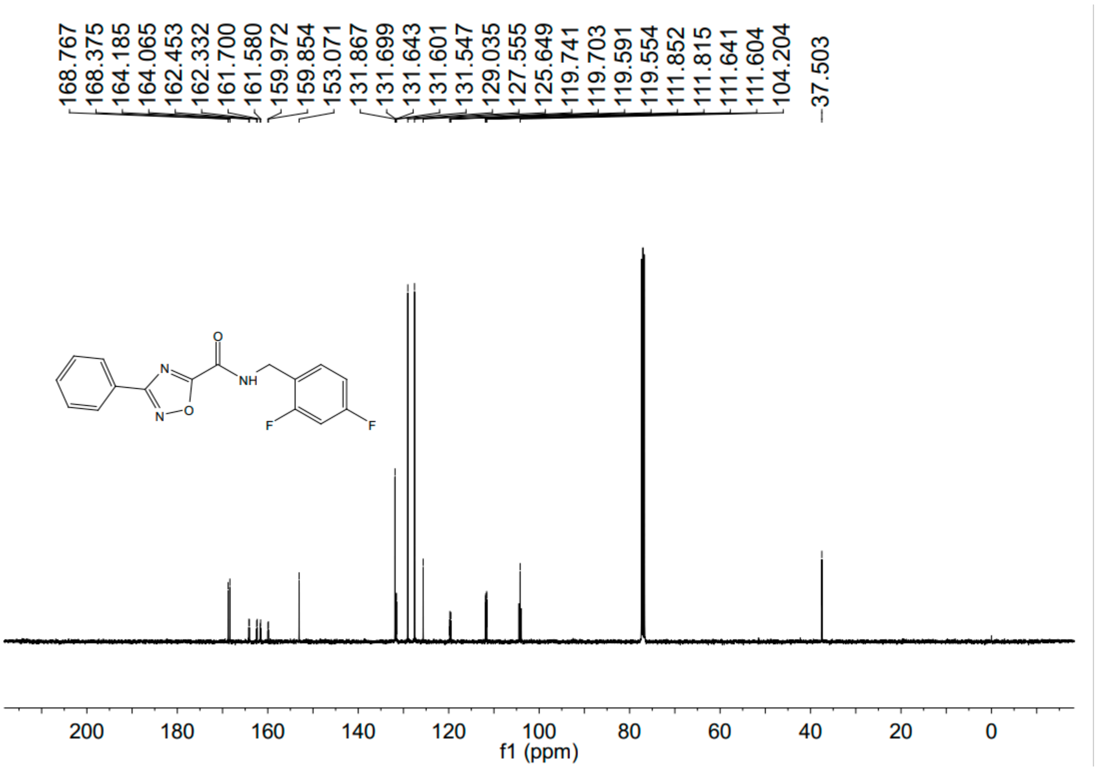

^{13}C NMR of compound **F3**

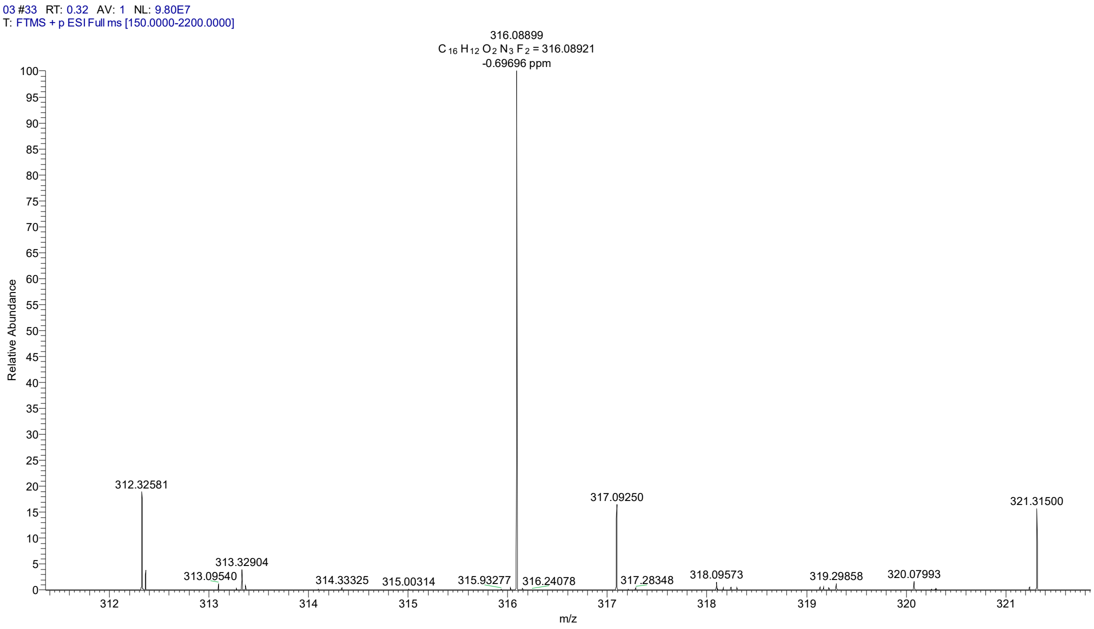

HRMS of compound **F3**

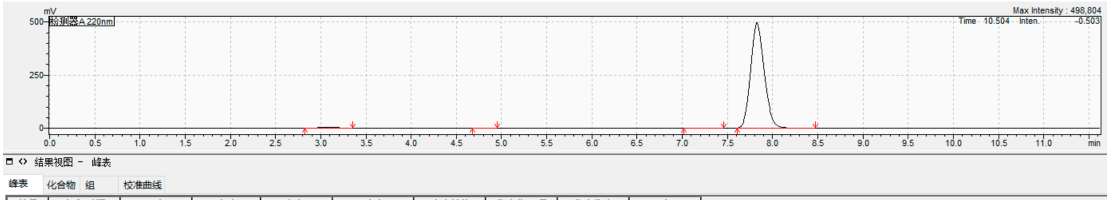

峰号	保留时间	面积	高度	标记	浓度	浓度单位	化合物ID号	化合物名	面积%
1	3.068	82863	5776		1.559				1.559
2	4.785	1265	191		0.024				0.024
3	7.147	2529	116		0.048				0.048
4	7.827	5230096	498066	M	98.370				98.370
总计		5316753	504148		100.000				100.000

HPLC of compound **F4**

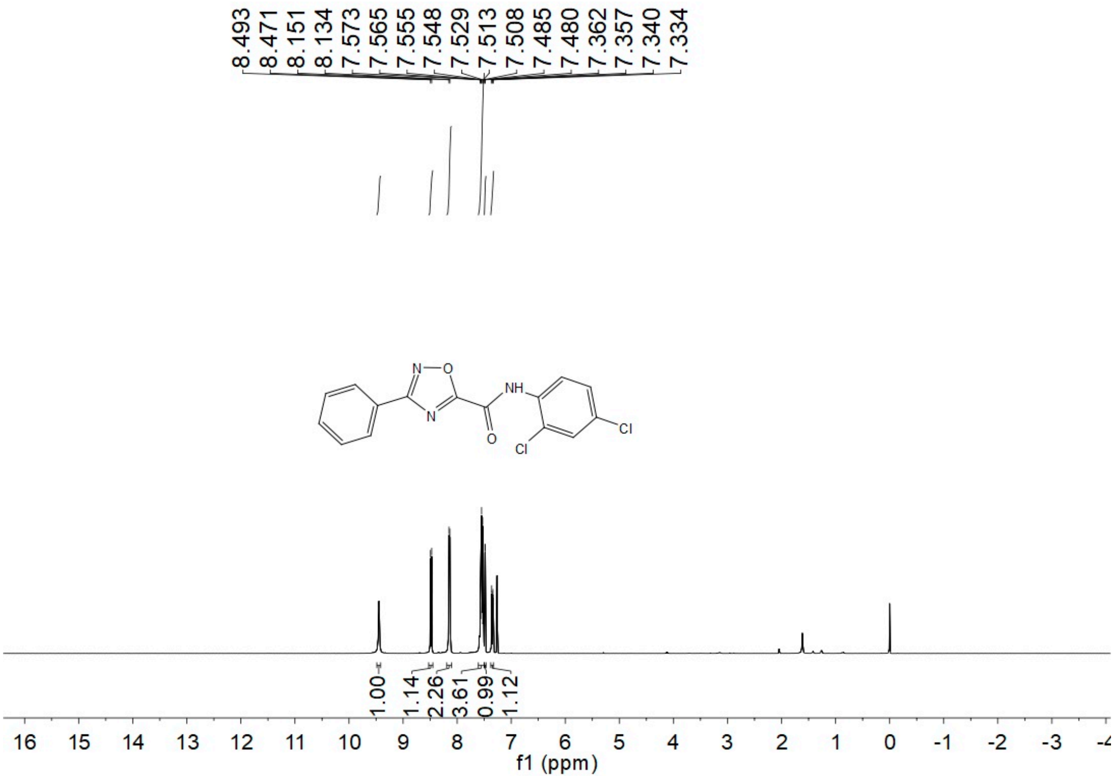

^{1}H NMR of compound **F4**

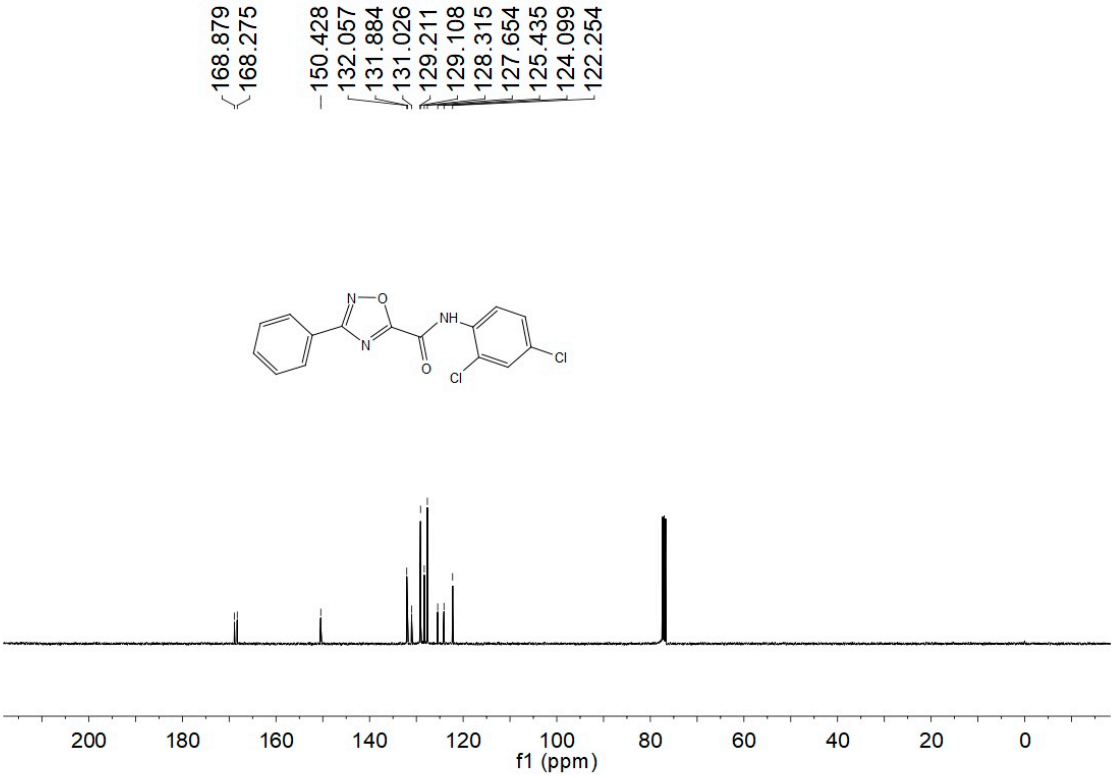

^{13}C NMR of compound **F4**

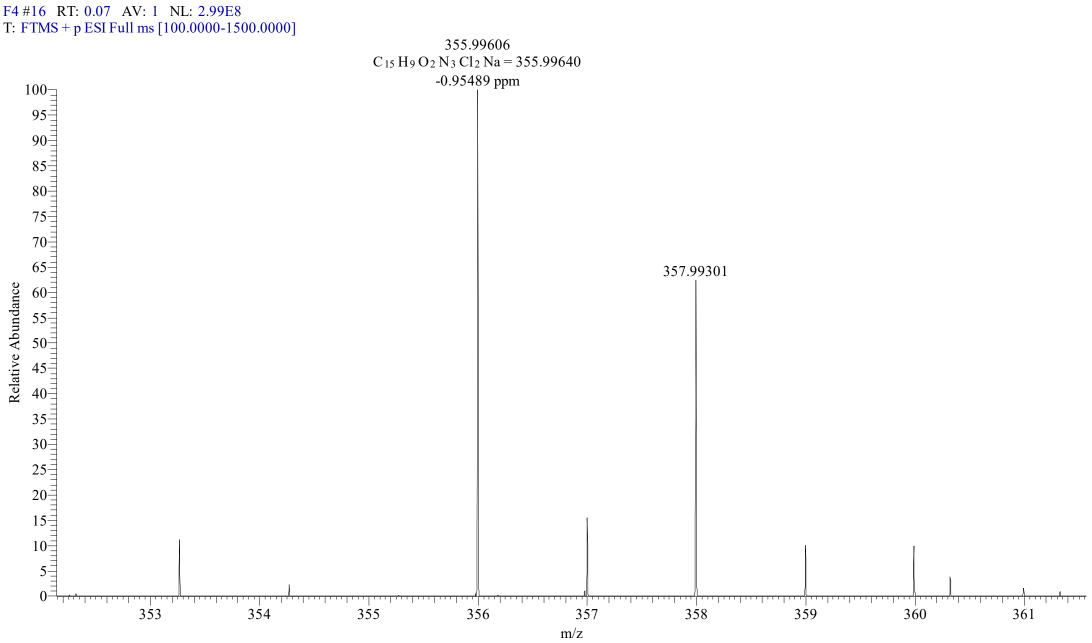

HRMS of compound **F4**

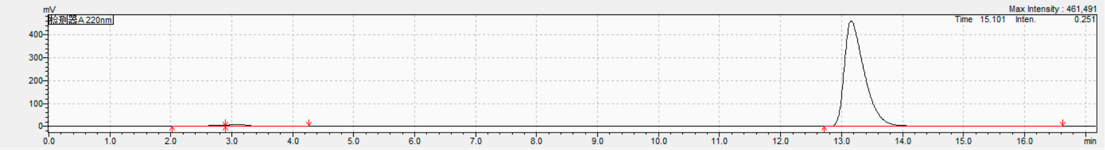

结果视图 - 峰表

峰表 化合物 组 校准曲线

峰号	保留时间	面积	高度	标记	浓度	浓度单位	化合物ID号	化合物名	面积%
1	2. 774	94462	3624		0. 870				0. 870
2	3. 070	199468	9168	SV	1. 837				1. 837
3	13. 159	10562911	461751		97. 293				97. 293
总计		10856841	474543		100. 000				100. 000

HPLC of compound **F5**

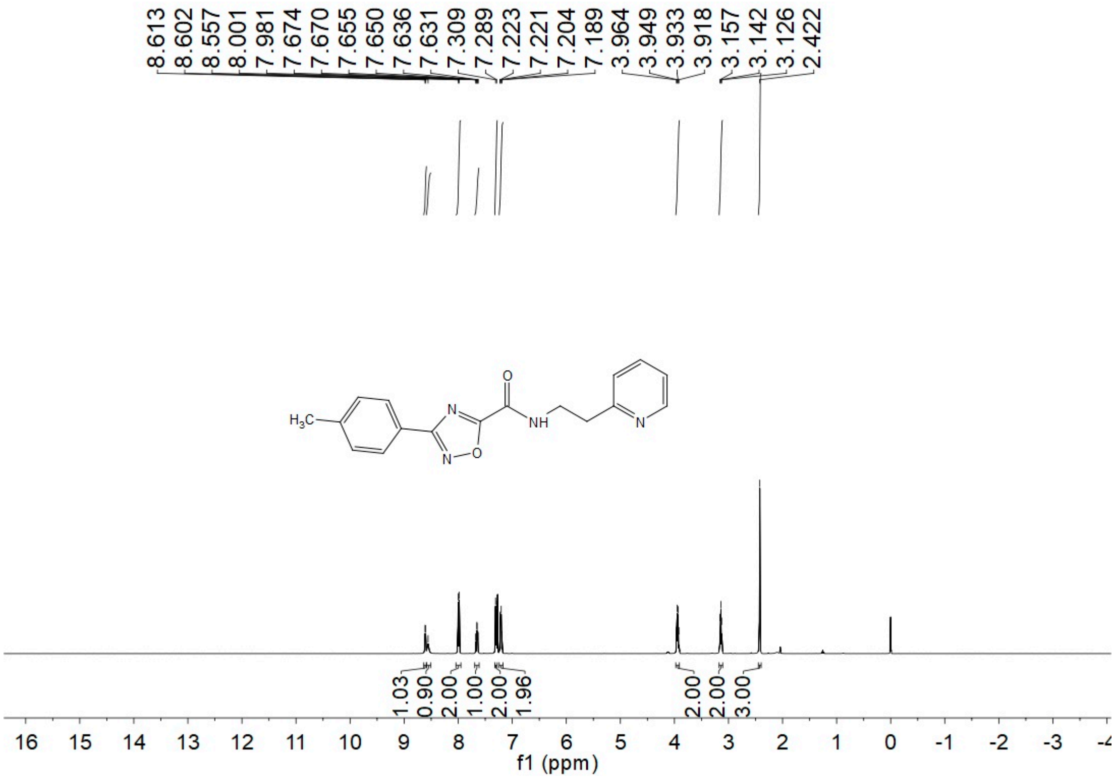

^{1}H NMR of compound **F5**

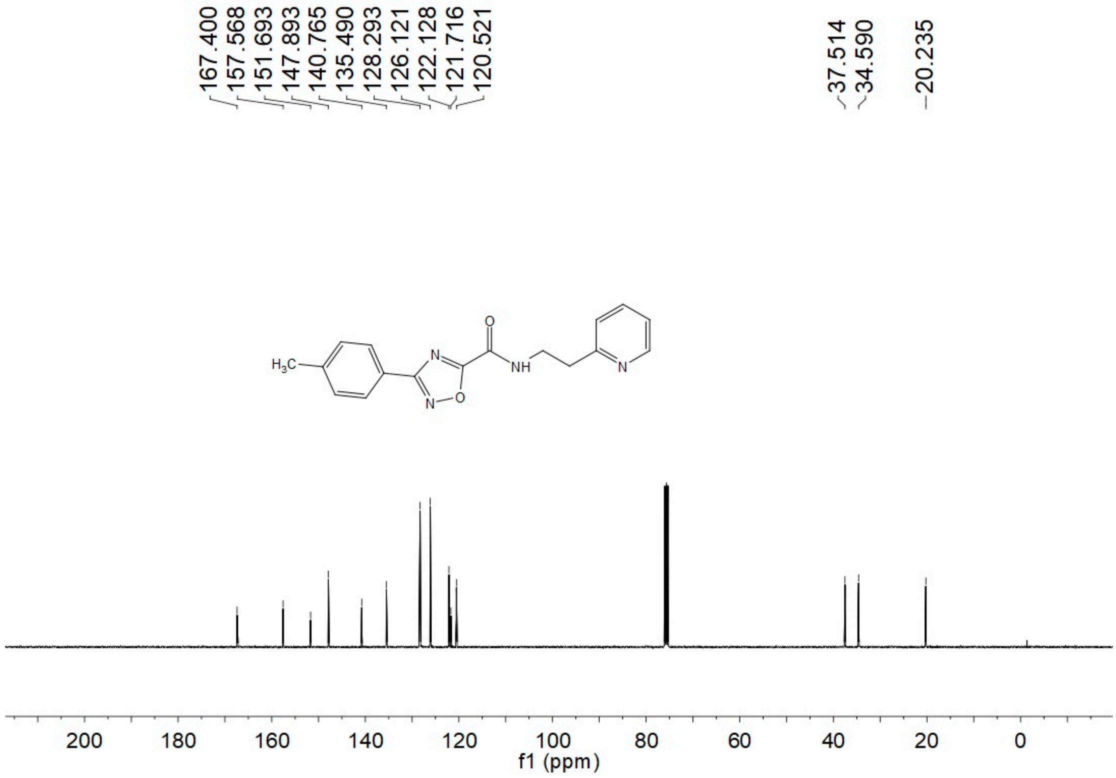

^{13}C NMR of compound **F5**

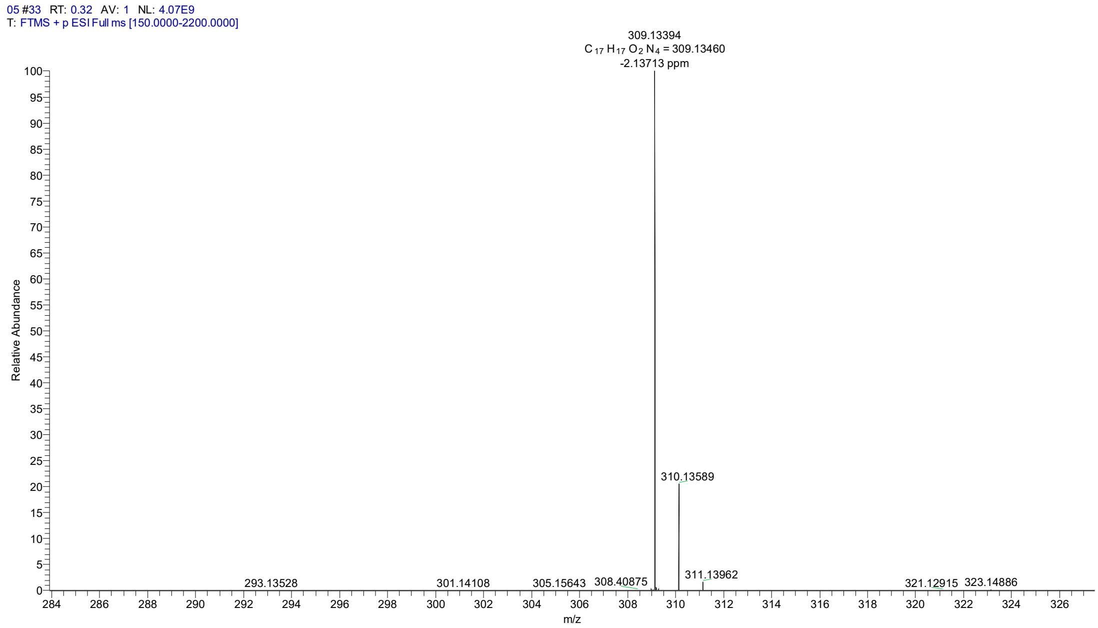

HRMS of compound **F5**

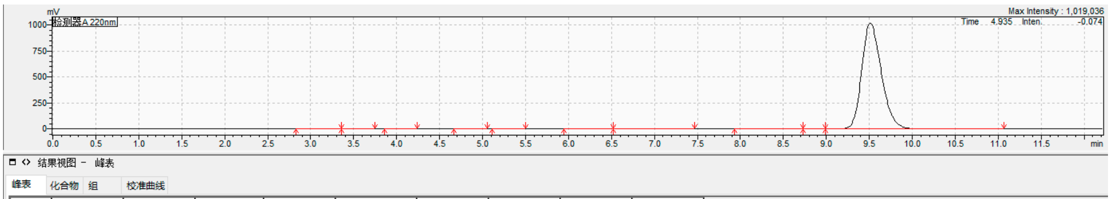

峰号	保留时间	面积	高度	标记	浓度	浓度单位	化合物ID号	化合物名	面积%
5	5.317	6898	859		0.042				0.042
6	6.314	10051	618		0.061				0.061
7	6.971	20646	1231		0.126				0.126
8	8.258	52251	2650		0.319				0.319
9	8.895	1167	99	V	0.007				0.007
10	9.512	16198329	1019277	V	98.880				98.880
总计		16381849	1031512		100.000				100.000

HPLC of compound **F6**

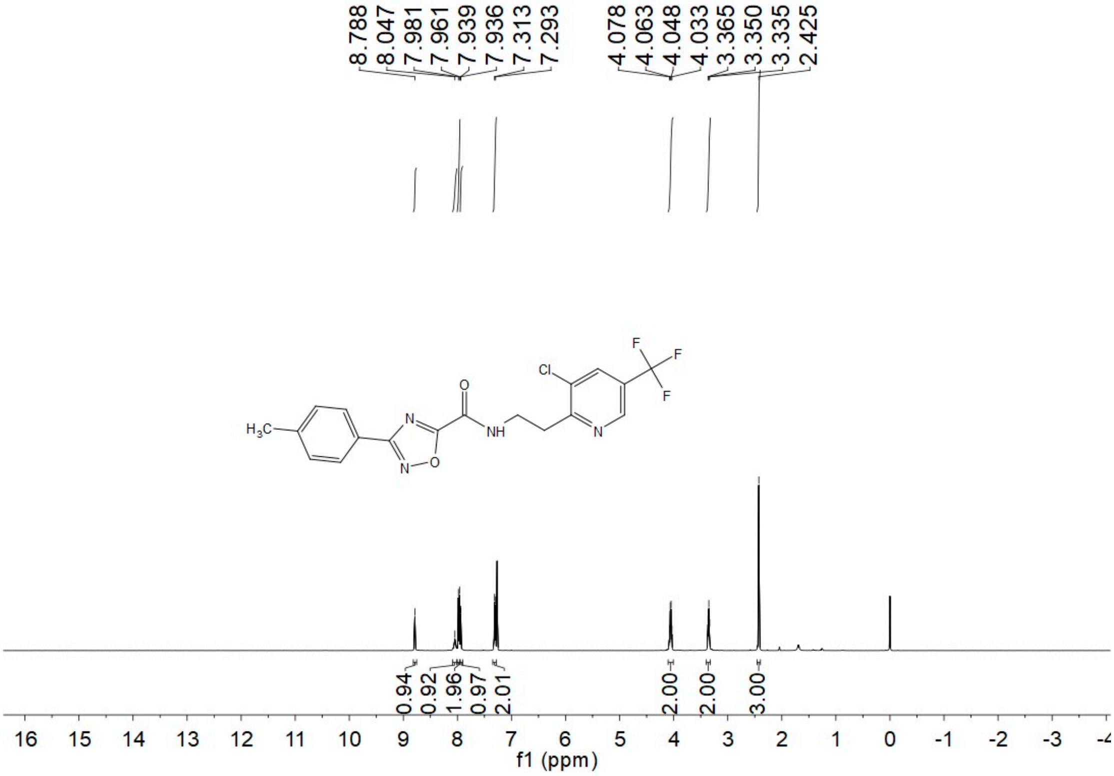

^{1}H NMR of compound **F6**

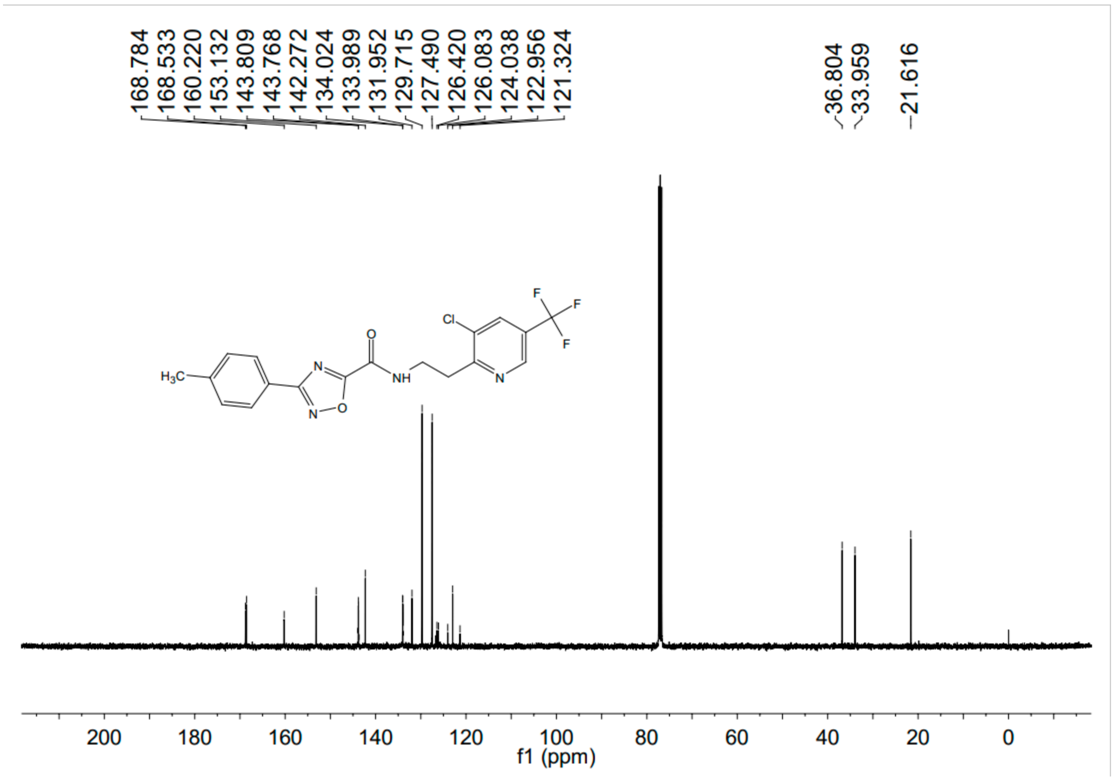

^{13}C NMR of compound **F6**

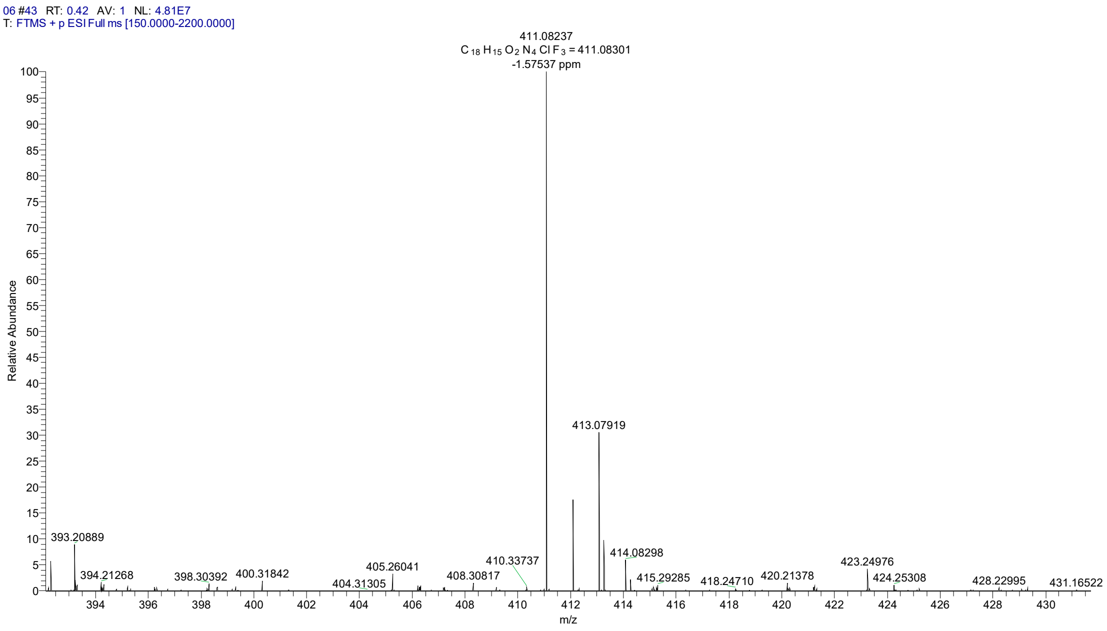

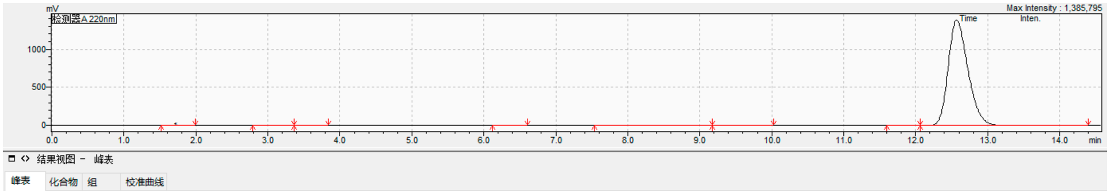

峰号	保留时间	面积	高度	标记	浓度	浓度单位	化合物ID号	化合物名	面积%
3	3.542	2442	189	V	0.000				0.009
4	6.259	5890	424		0.000				0.022
5	8.329	61980	2666		0.000				0.234
6	9.554	29839	1774	V	0.000				0.113
7	11.816	2324	147	V	0.000				0.009
8	12.569	26269032	1386597	S	0.000				99.212
总计		26477778	1401765		0.000				100.000

HRMS of compound **F6**

HPLC of compound **F7**

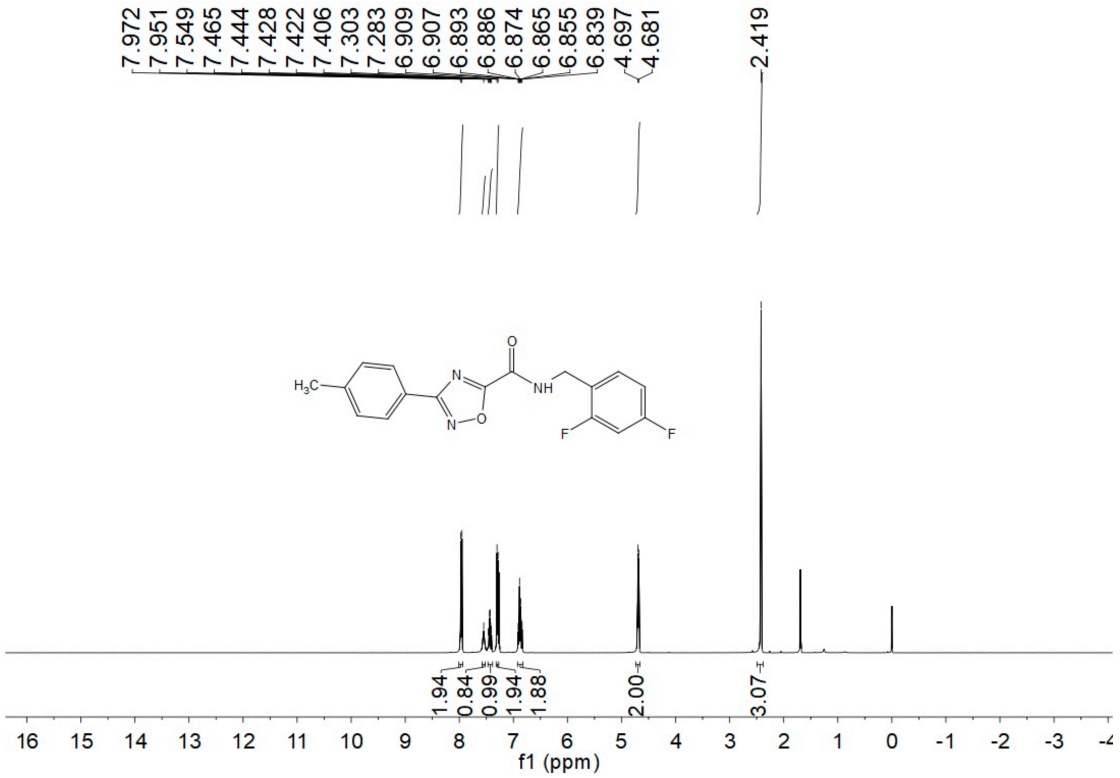

^{1}H NMR of compound **F7**

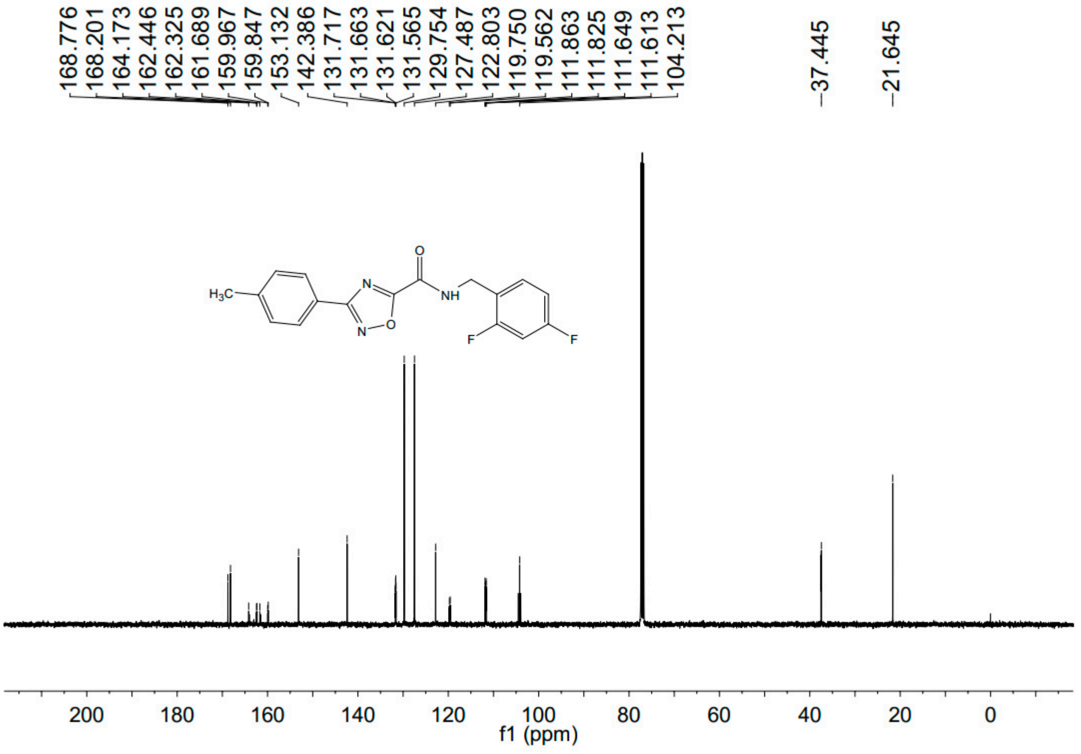

^{13}C NMR of compound **F7**

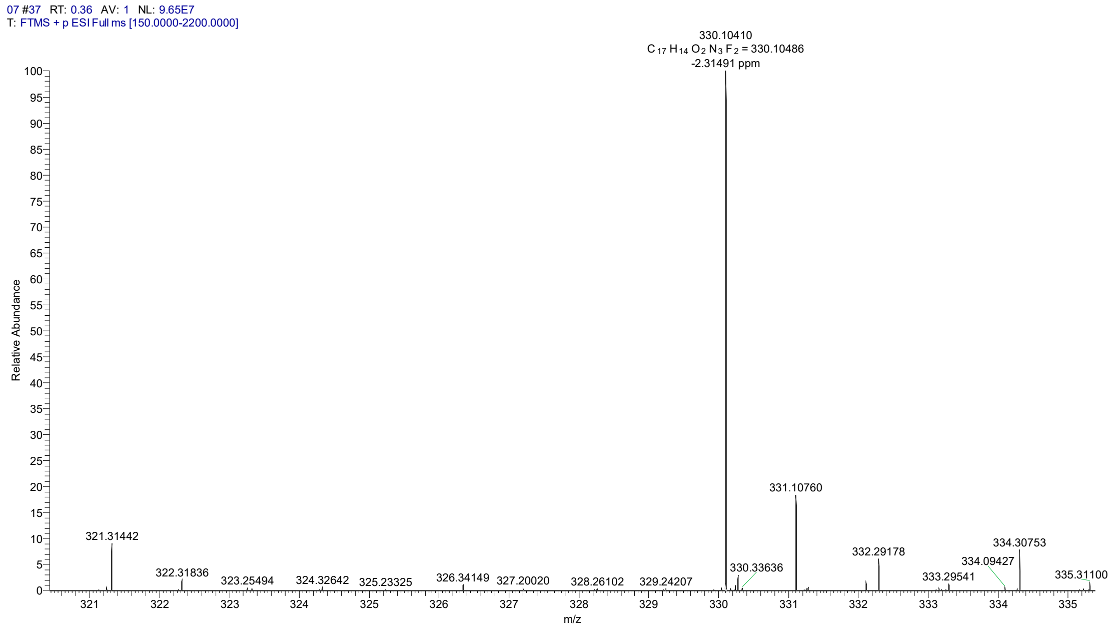

HRMS of compound **F7**

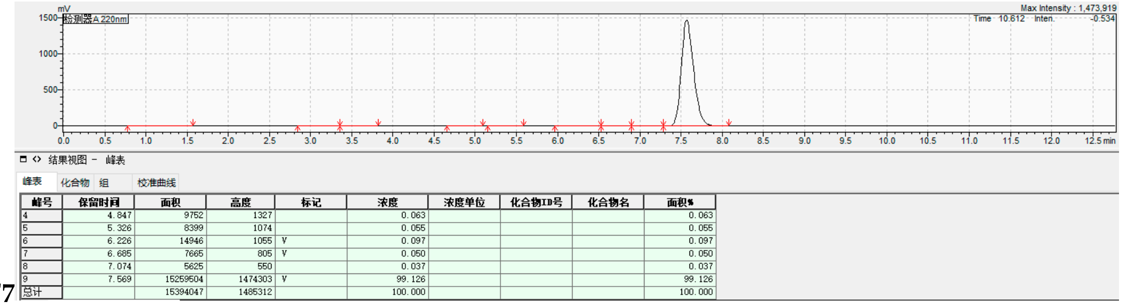

峰号	保留时间	面积	高度	标记	浓度	浓度单位	化合物ID号	化合物名	面积%
4	4.847	9752	1327		0.063				0.063
5	5.326	8399	1074		0.055				0.055
6	6.226	14946	1055	V	0.097				0.097
7	6.685	7665	805	V	0.050				0.050
8	7.074	5625	550		0.037				0.037
9	7.569	15259504	1474303	V	99.126				99.126
总计		15394047	1485312		100.000				100.000

HPLC of compound **F8**

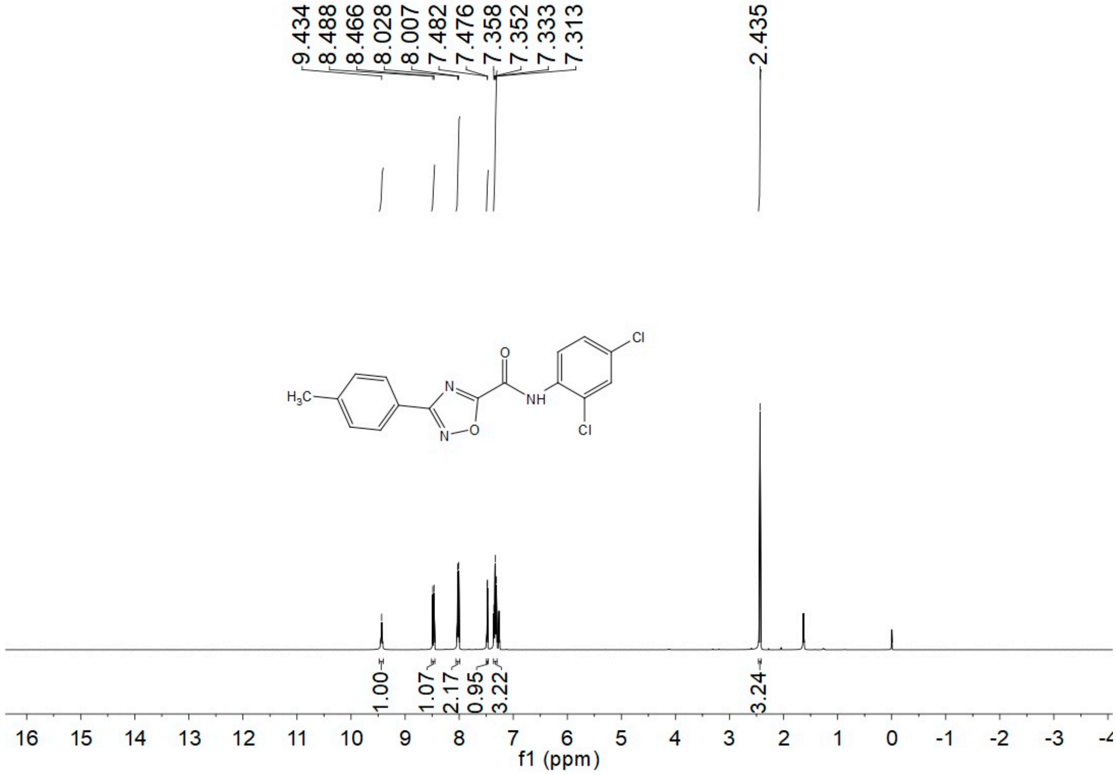

^{1}H NMR of compound **F8**

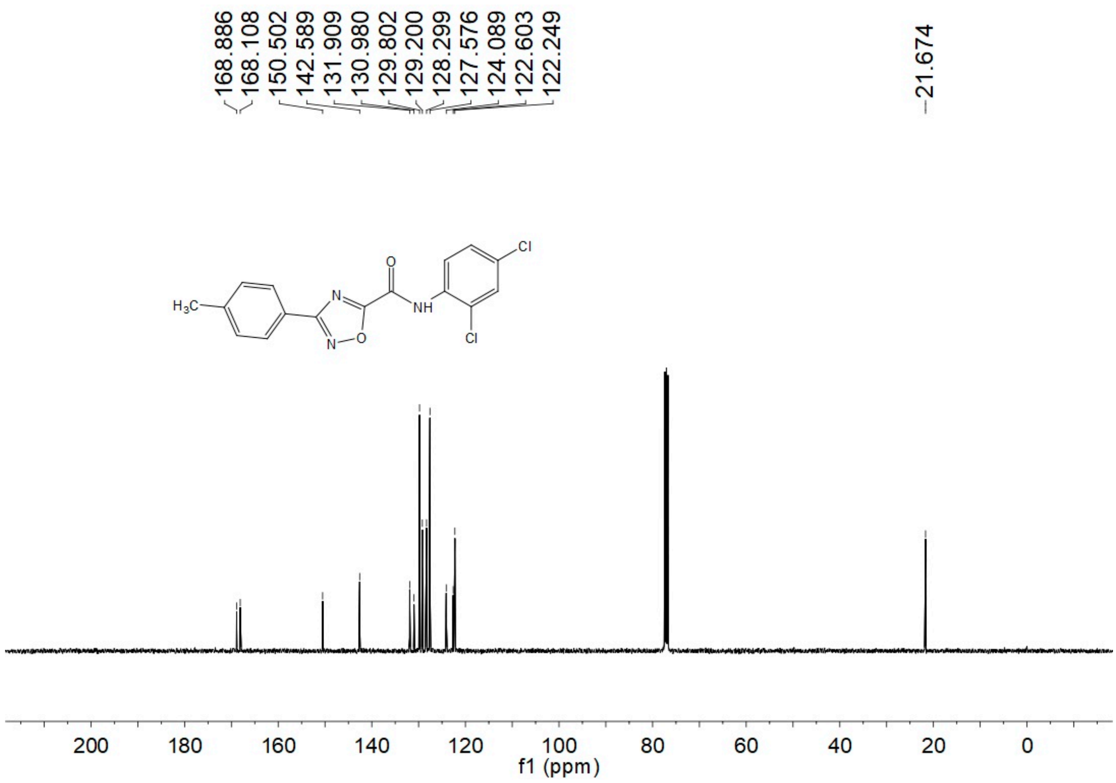

^{13}C NMR of compound **F8**

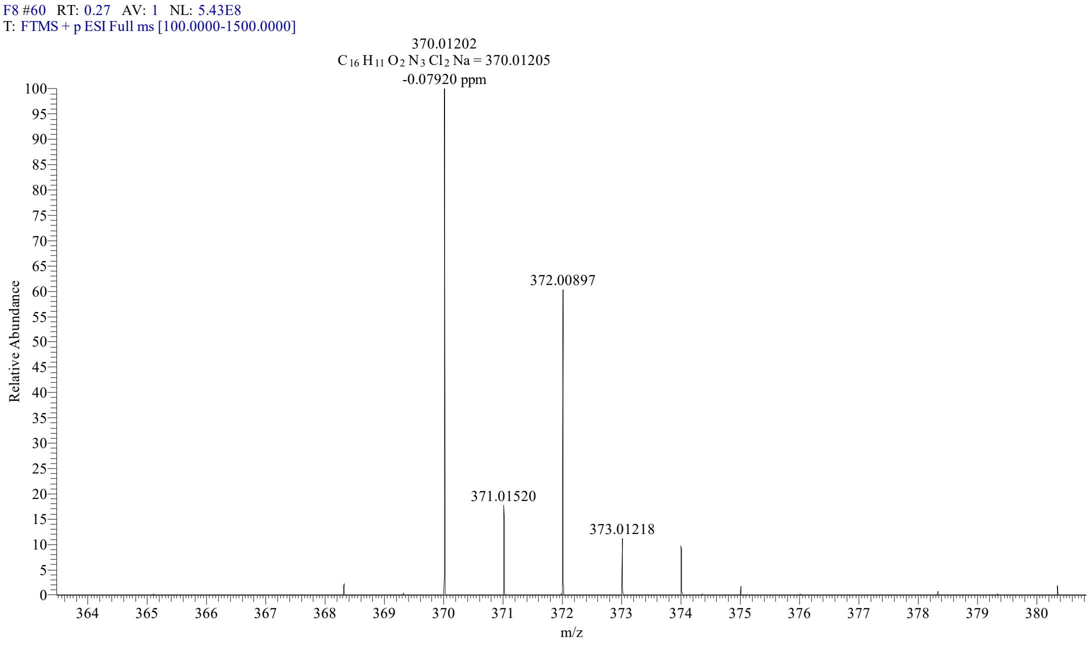

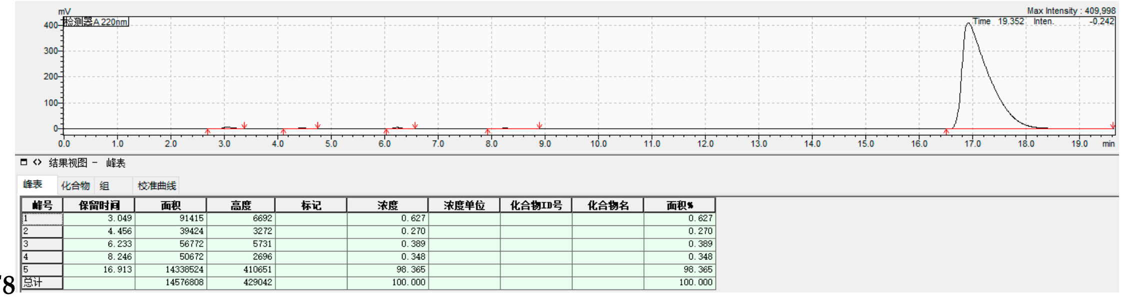

峰号	保留时间	面积	高度	标记	浓度	浓度单位	化合物ID号	化合物名	面积%
1	3.049	91415	6692		0.627				0.627
2	4.456	39424	3272		0.270				0.270
3	6.233	56772	5731		0.389				0.389
4	8.246	50672	2696		0.348				0.348
5	16.913	14338524	410651		98.365				98.365
总计		14576808	429042		100.000				100.000

HRMS of compound **F8**

HPLC of compound **F9**

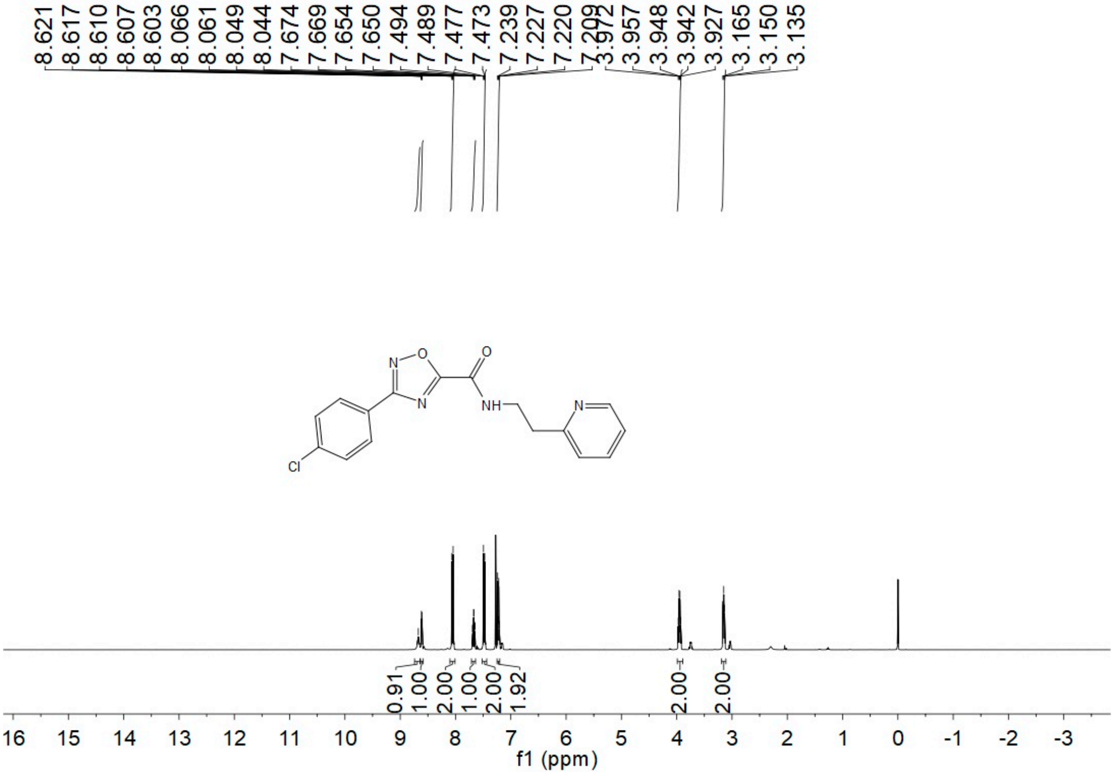

^{1}H NMR of compound **F9**

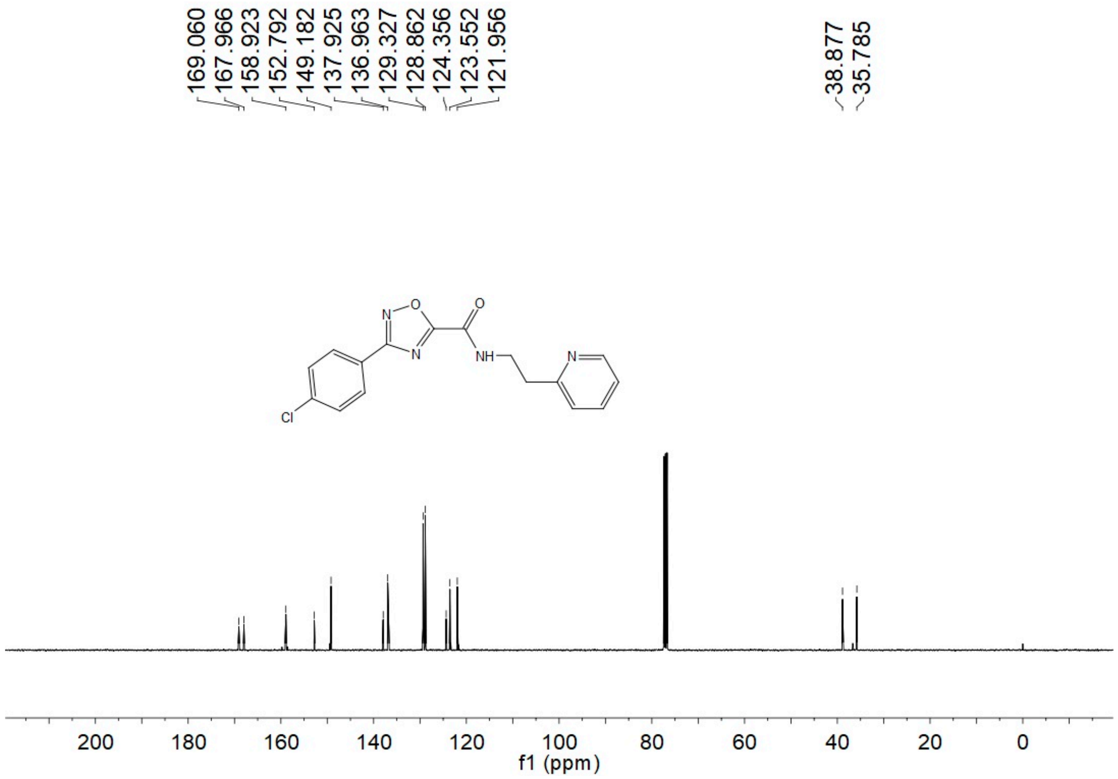

^{13}C NMR of compound **F9**

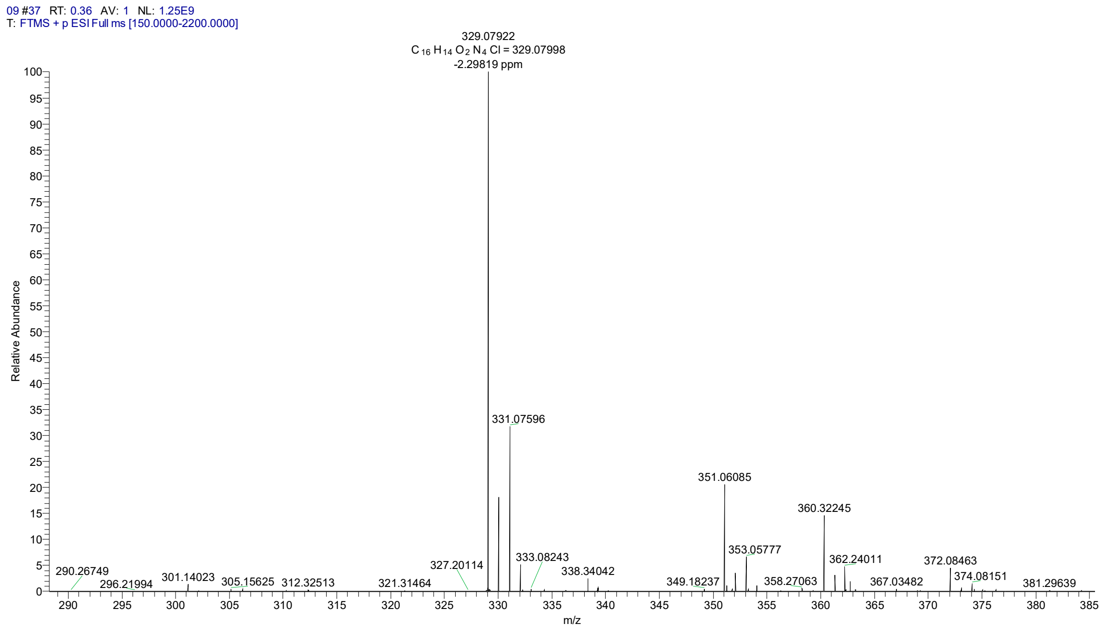

HRMS of compound **F9**

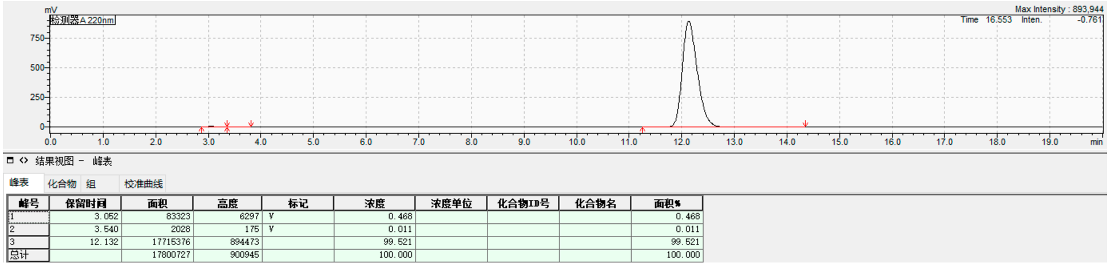

峰号	保留时间	面积	高度	标记	浓度	浓度单位	化合物ID号	化合物名	面积%
1	3.052	83323	6297	V	0.468				0.468
2	3.540	2028	175	V	0.011				0.011
3	12.132	17715376	894473		99.521				99.521
总计		17800727	900945		100.000				100.000

HPLC of compound **F10**

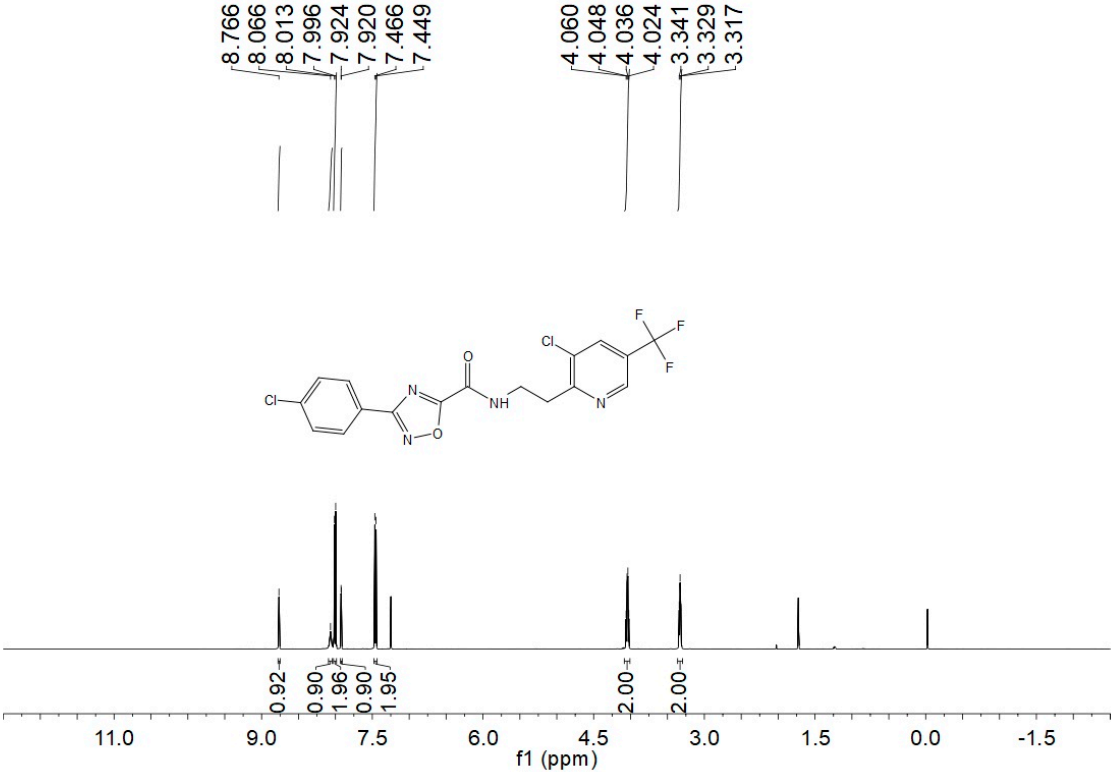

^{1}H NMR of compound **F10**

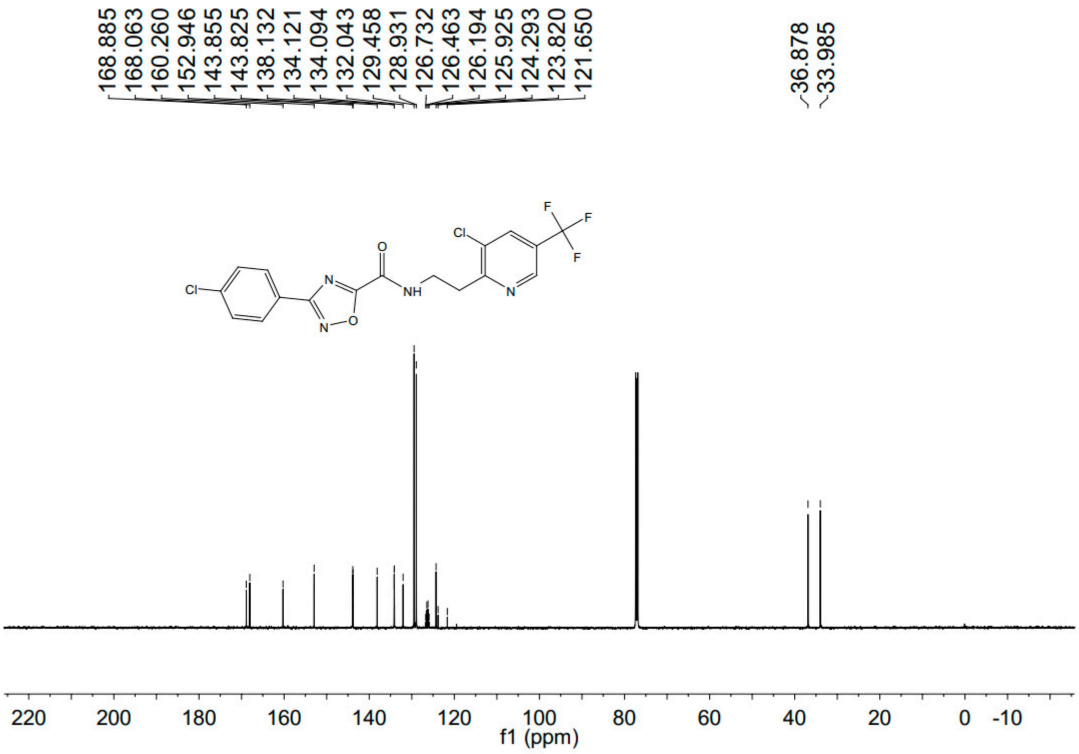

^{13}C NMR of compound **F10**

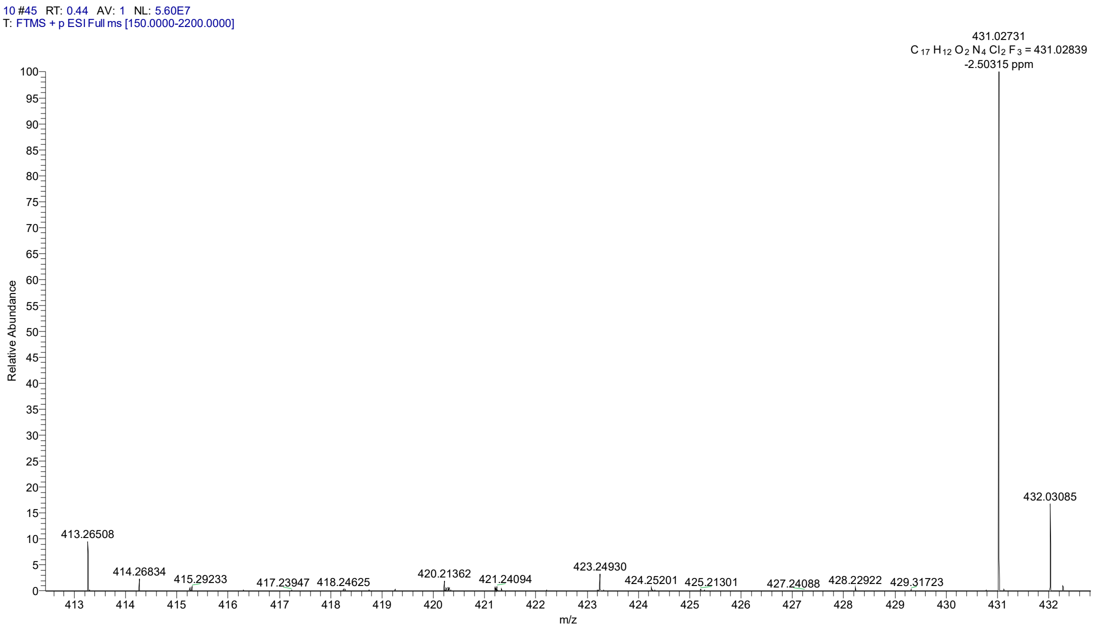

HRMS of compound **F10**

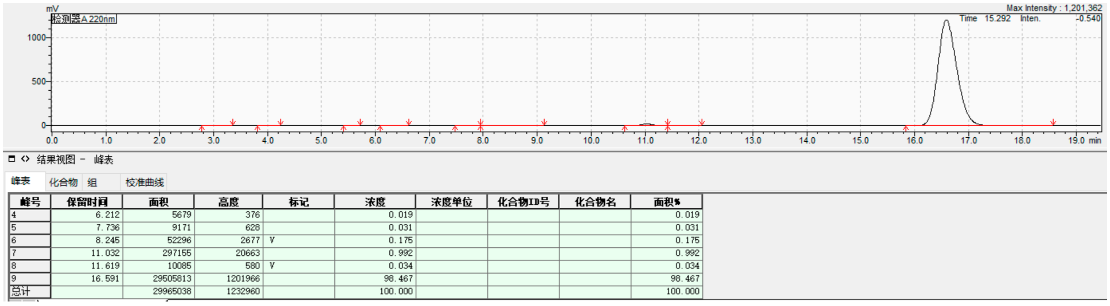

峰号	保留时间	面积	高度	标记	浓度	浓度单位	化合物ID号	化合物名	面积%
4	6.212	5679	376		0.019				0.019
5	7.736	9171	628		0.031				0.031
6	8.245	52296	2677	V	0.175				0.175
7	11.032	297155	20663		0.992				0.992
8	11.619	10085	580	V	0.034				0.034
9	16.591	29505813	1201966		98.467				98.467
总计		29965038	1232960		100.000				100.000

HPLC of compound **F11**

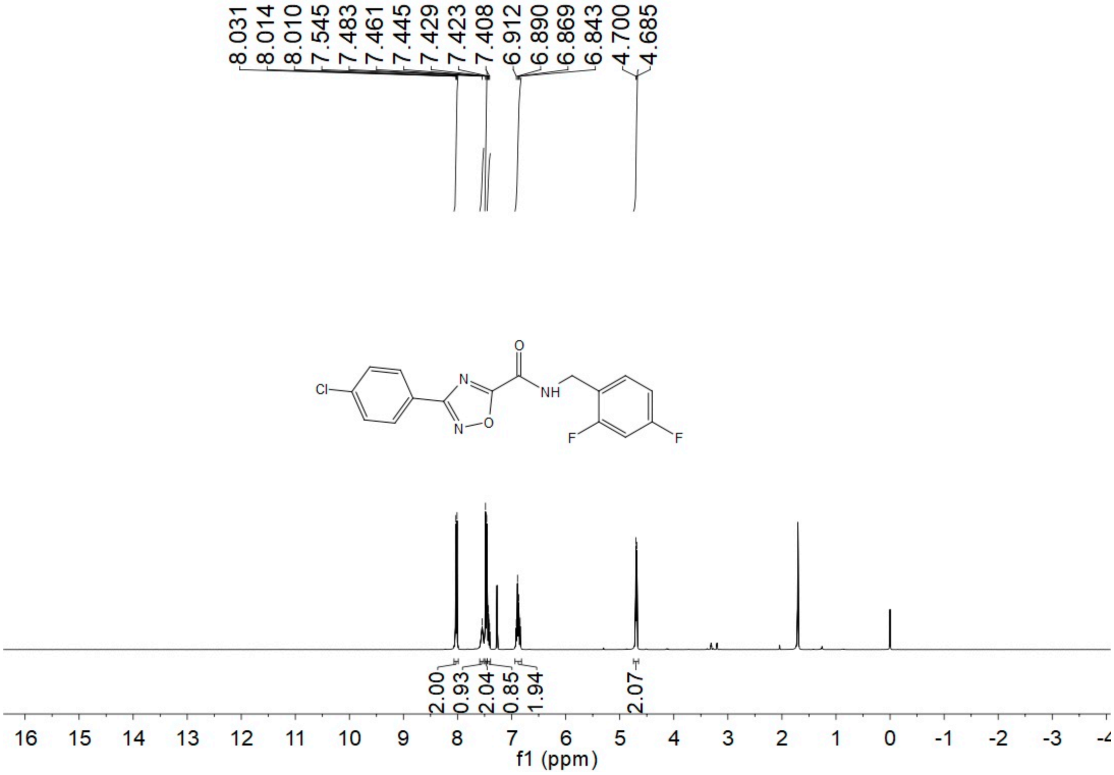

^{1}H NMR of compound **F11**

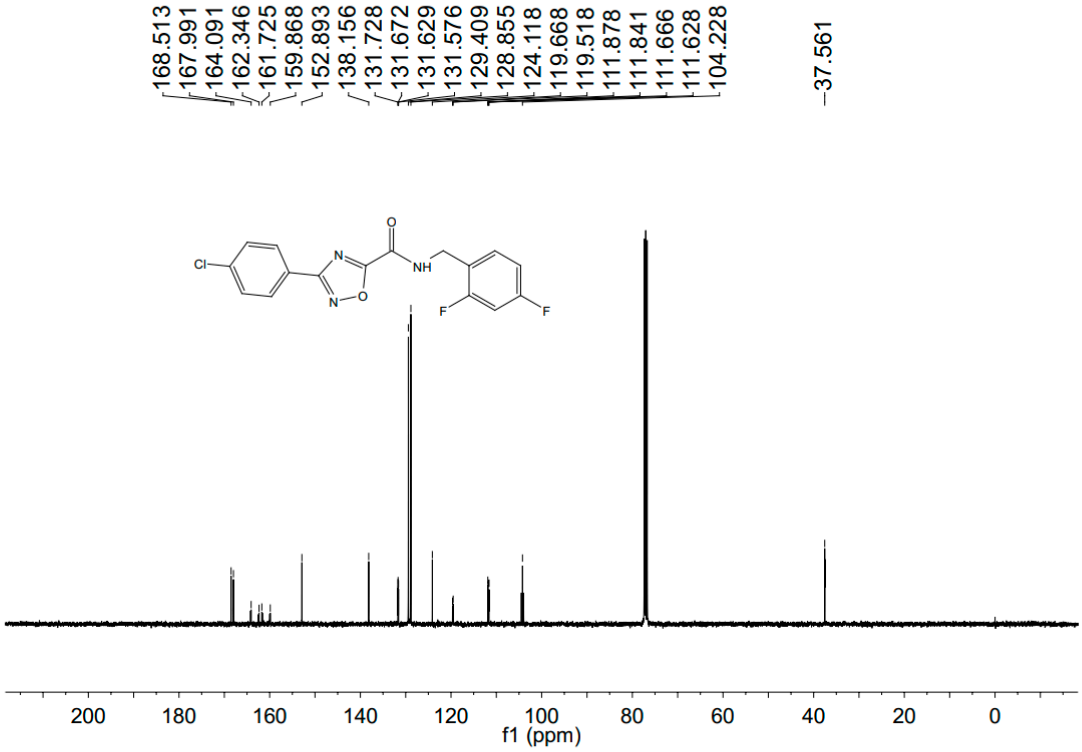

^{13}C NMR of compound **F11**

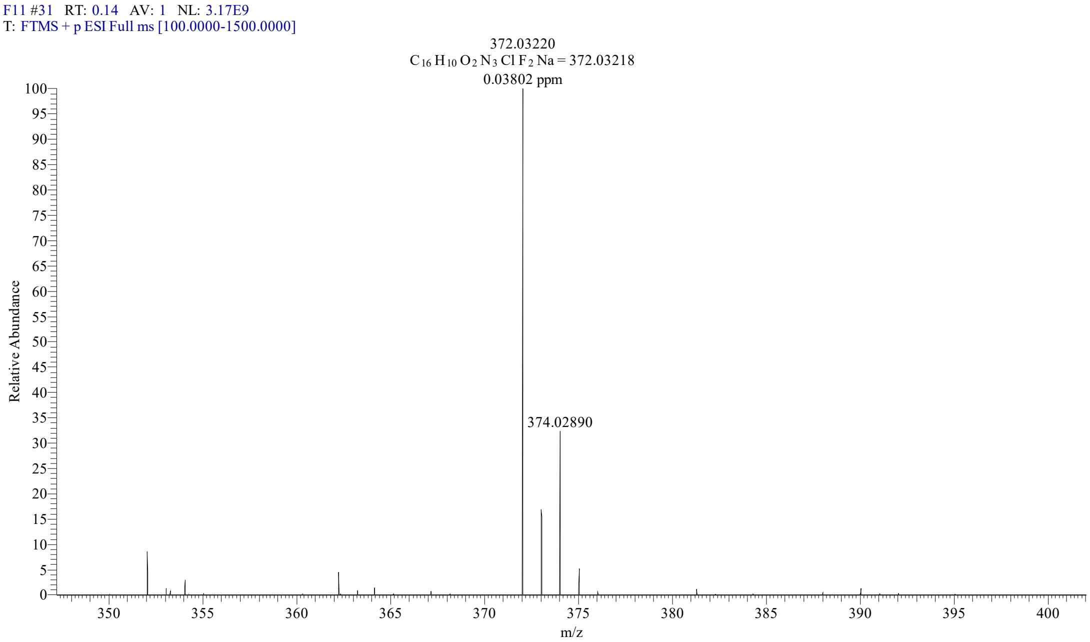

HRMS of compound **F11**

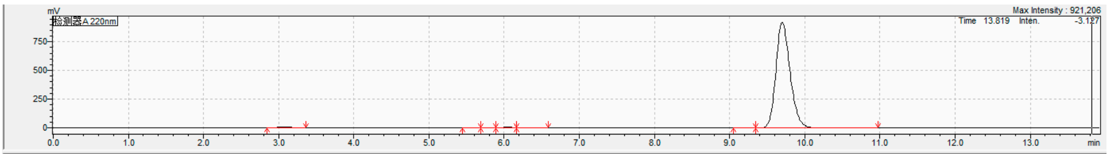

结果视图 - 峰表

峰表 化合物 组 校准曲线

峰号	保留时间	面积	高度	标记	浓度	浓度单位	化合物ID号	化合物名	面积%
2	5. 594	1921	258		0. 016				0. 016
3	5. 794	4398	547	V	0. 036				0. 036
4	6. 040	63228	8004	V	0. 517				0. 517
5	6. 303	38811	3403	V	0. 317				0. 317
6	9. 177	1696	170	V	0. 014				0. 014
7	9. 700	12051844	923246		98. 460				98. 460
总计		12240386	941152		100. 000				100. 000

HPLC of compound **F12**

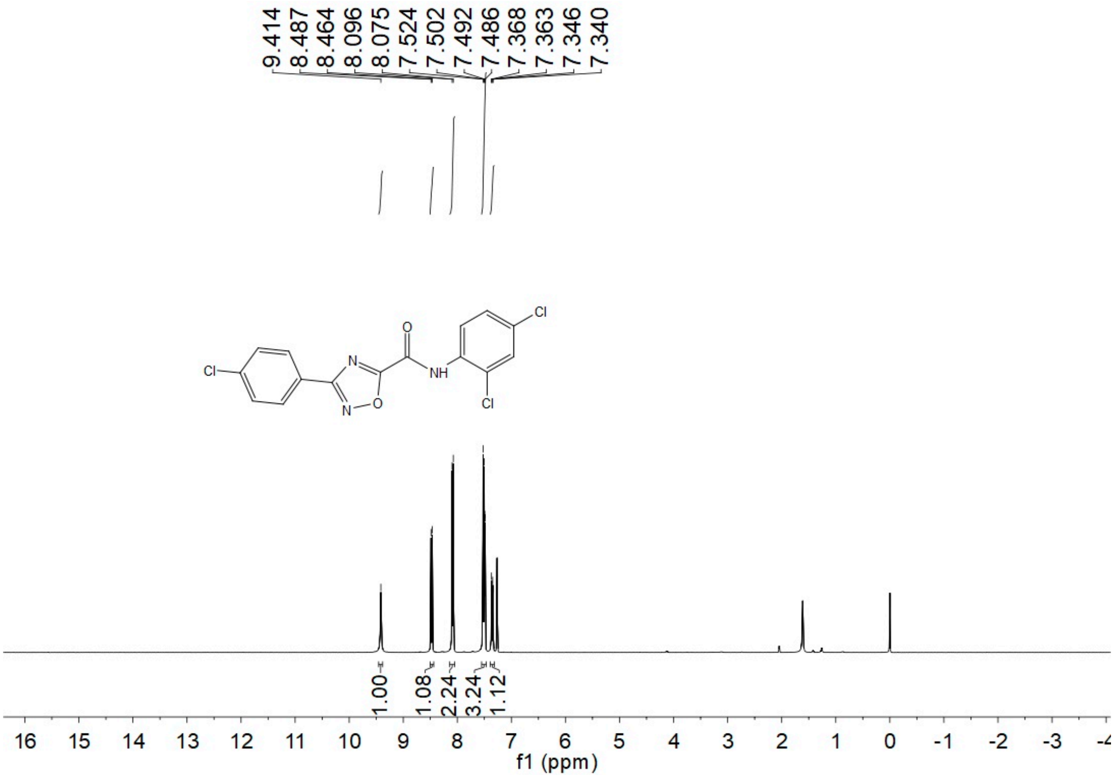

^{1}H NMR of compound **F12**

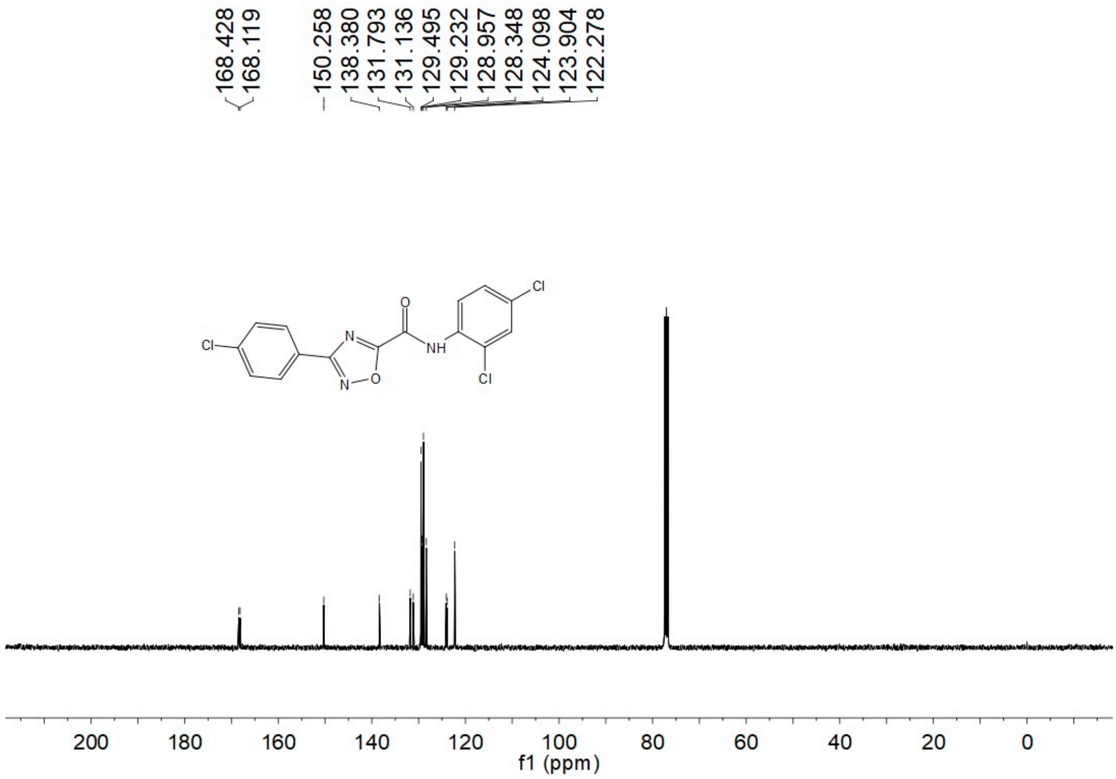

^{13}C NMR of compound **F12**

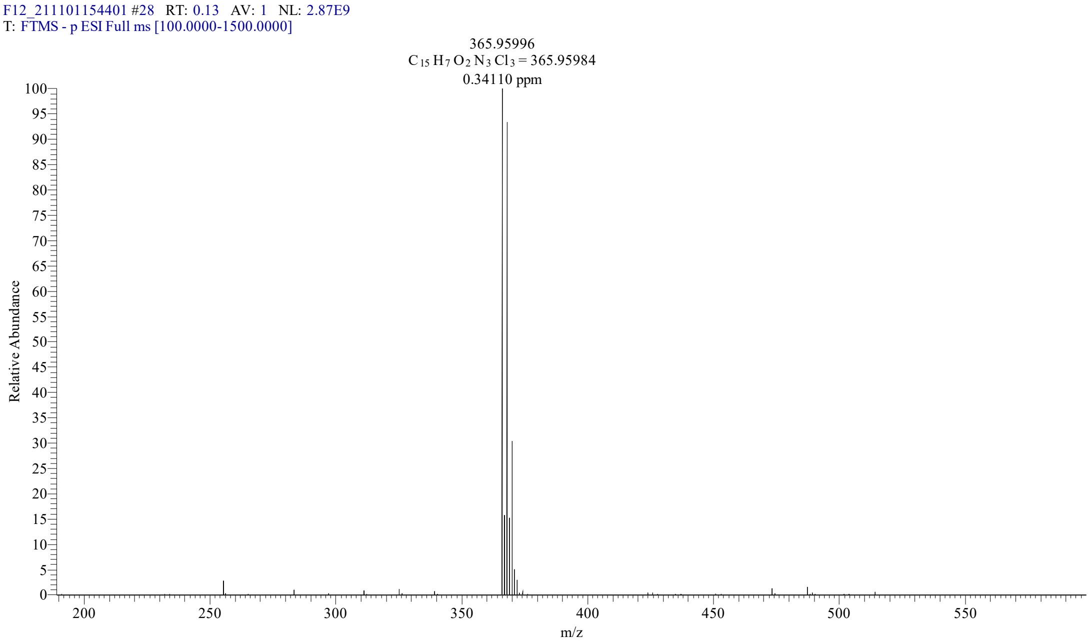

HRMS of compound **F12**

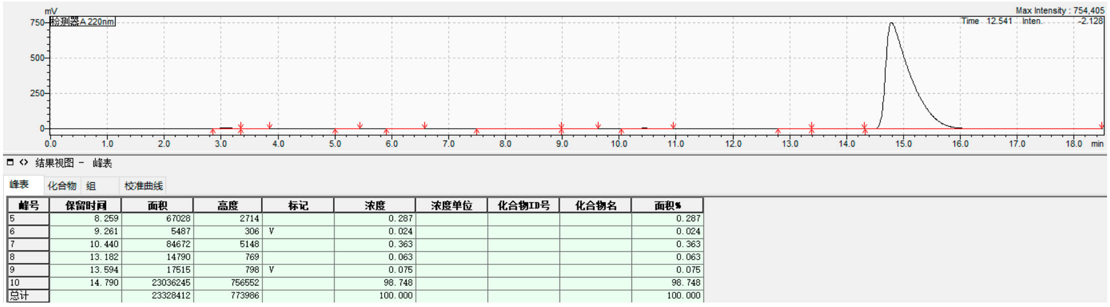

峰号	保留时间	面积	高度	标记	浓度	浓度单位	化合物ID号	化合物名	面积%
5	8.259	67028	2714		0.287				0.287
6	9.261	5487	306	V	0.024				0.024
7	10.440	84672	5148		0.363				0.363
8	13.182	14790	769		0.063				0.063
9	13.594	17515	798	V	0.075				0.075
10	14.790	23036245	756552		98.748				98.748
总计		23328412	773986		100.000				100.000

HPLC of compound **F13**

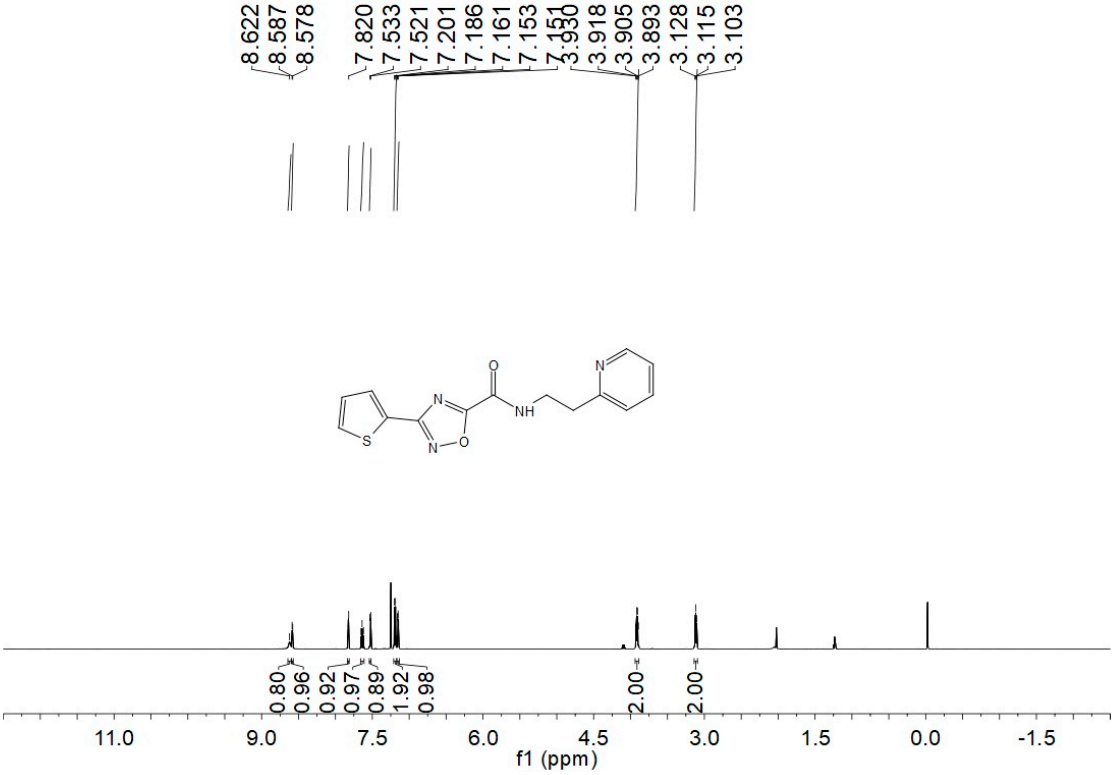

^{1}H NMR of compound **F13**

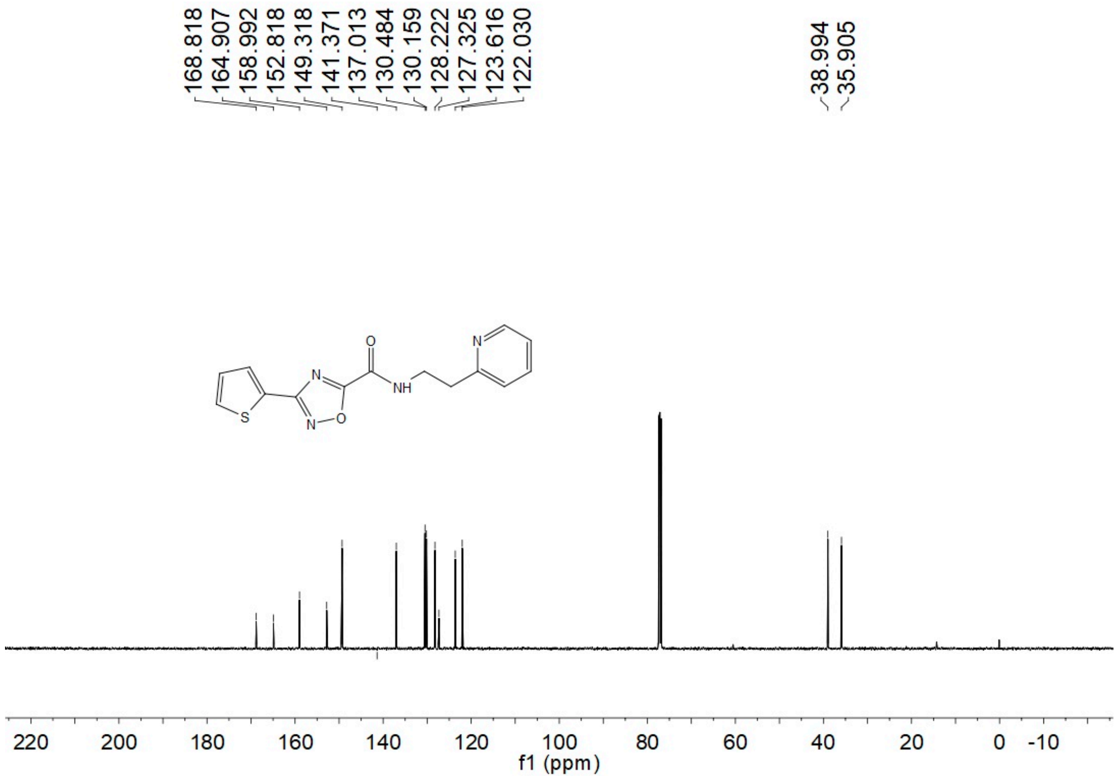

^{13}C NMR of compound **F13**

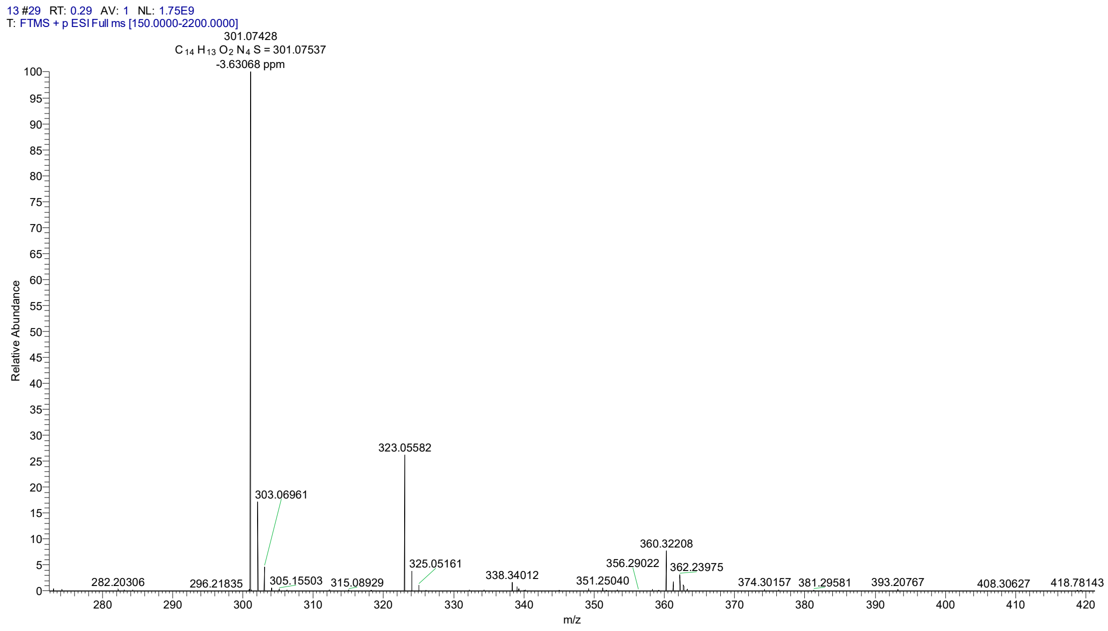

HRMS of compound **F13**

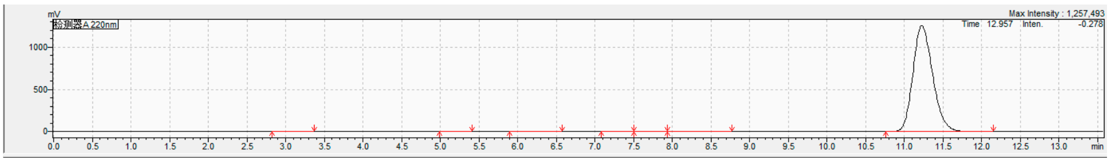

结果视图 － 峰表

峰表 化合物 组 校准曲线

峰号	保留时间	面积	高度	标记	浓度	浓度单位	化合物ID号	化合物名	面积%
2	5.191	1338	117		0.006				0.006
3	6.205	6518	397		0.029				0.029
4	7.181	4075	284		0.018				0.018
5	7.744	3819	220		0.017				0.017
6	8.249	44712	2513	V	0.199				0.199
7	11.228	22310667	1258186		99.382				99.382
总计		22449368	1267214		100.000				100.000

HPLC of compound **F14**

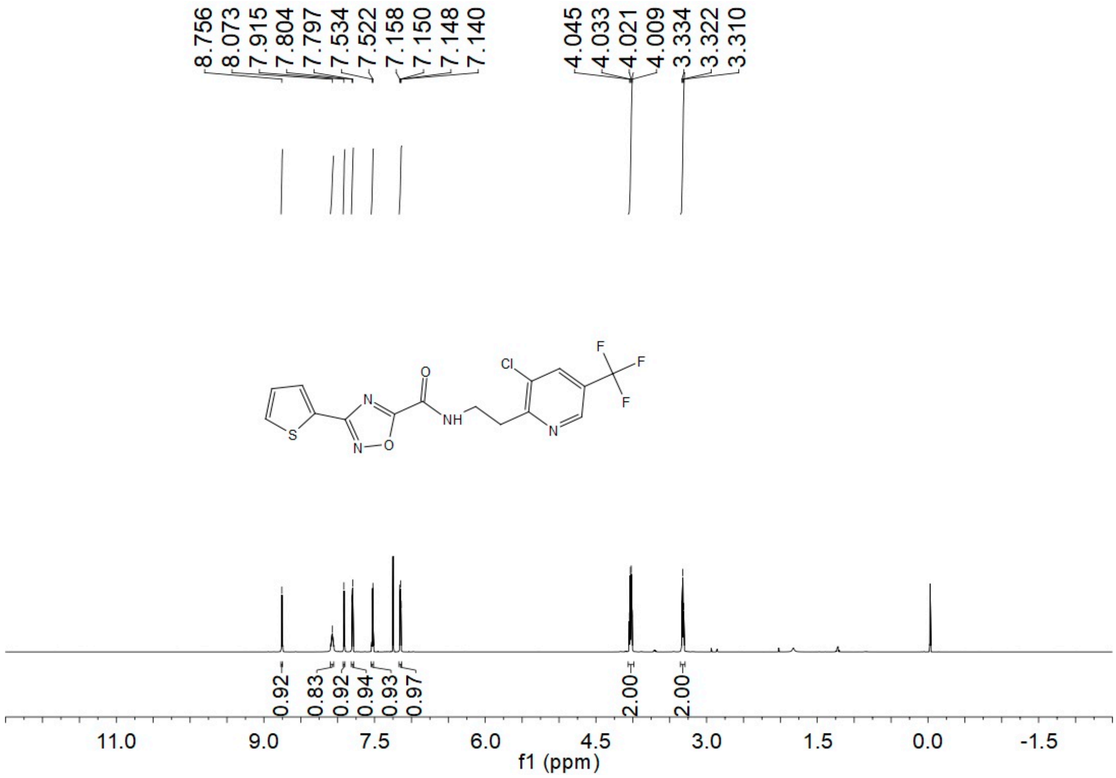

^{1}H NMR of compound **F14**

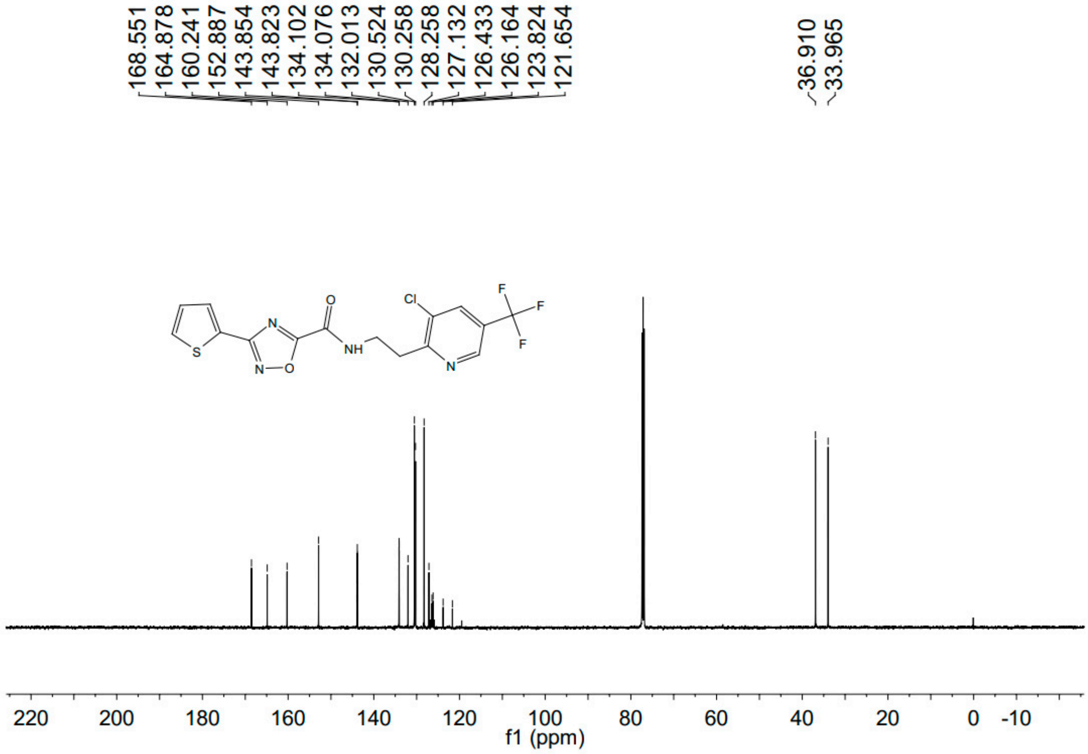

^{13}C NMR of compound **F14**

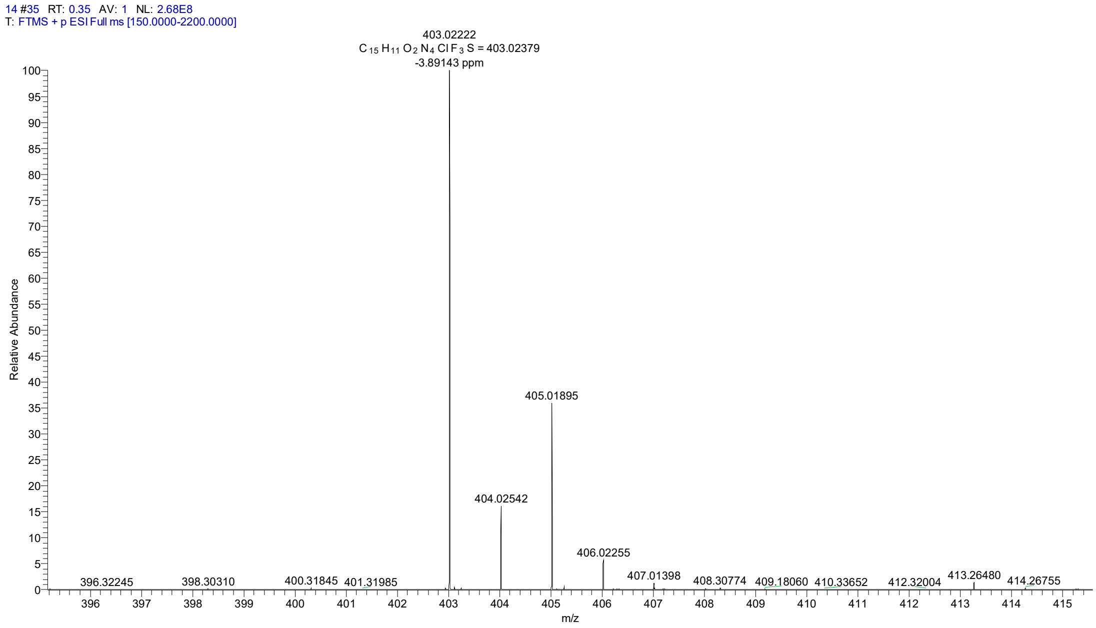

HRMS of compound **F14**

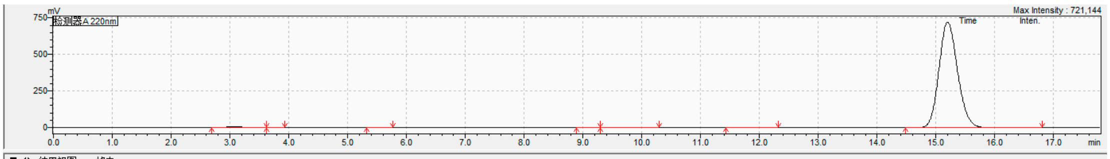

结果视图 - 峰表

峰表 化合物 组 校准曲线

峰号	保留时间	面积	高度	标记	浓度	浓度单位	化合物ID号	化合物名	面积%
2	3.754	4689	580		0.029				0.029
3	5.514	14651	1646		0.091				0.091
4	9.189	2580	180		0.016				0.016
5	9.585	43406	2074	V	0.270				0.270
6	11.828	13313	626		0.083				0.083
7	15.199	15915322	722723		98.832				98.832
总计		16103409	735366		100.000				100.000

HPLC of compound **F15**

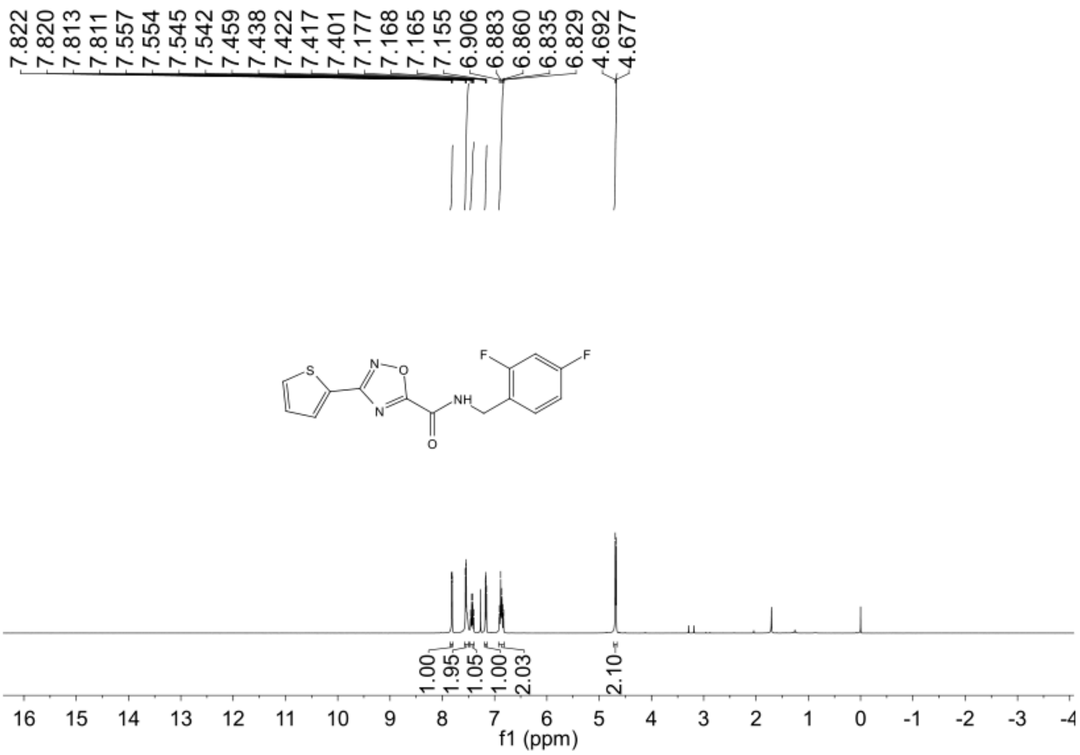

^{1}H NMR of compound **F15**

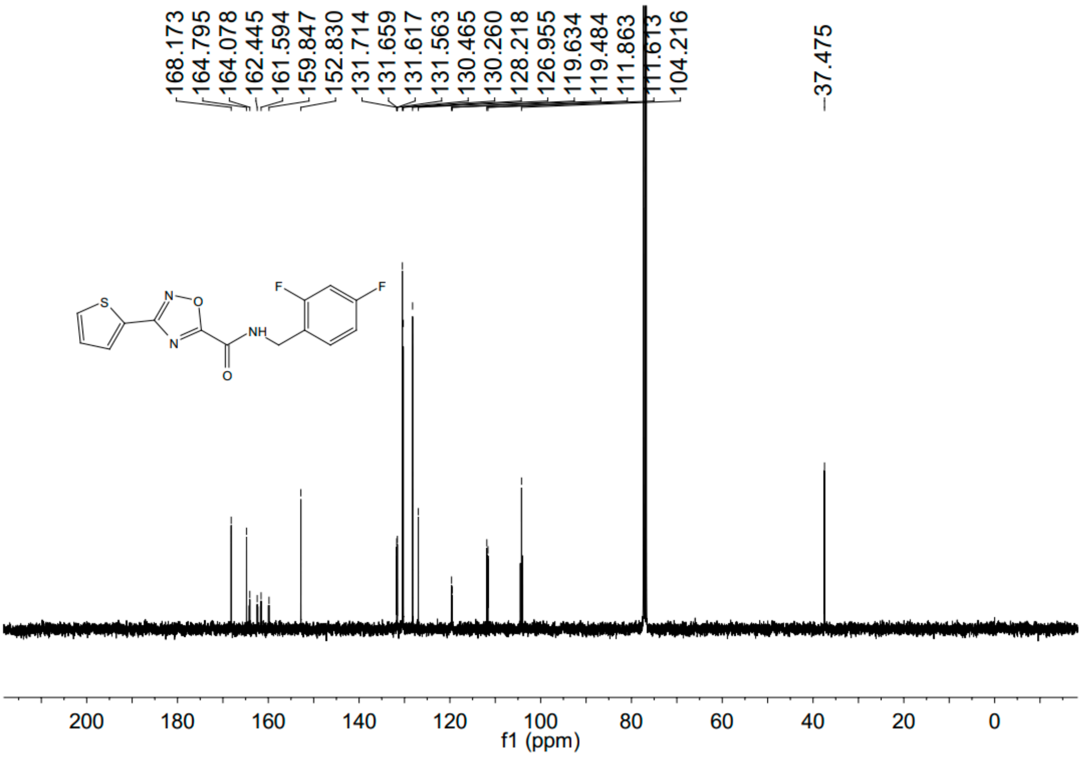

^{13}C NMR of compound **F15**

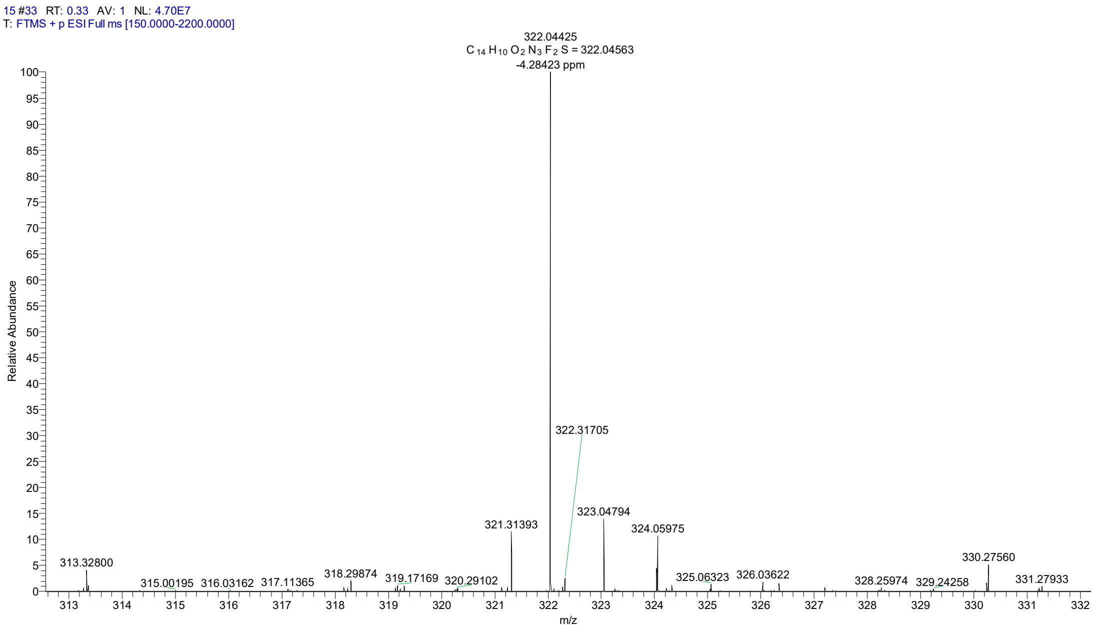

HRMS of compound **F15**

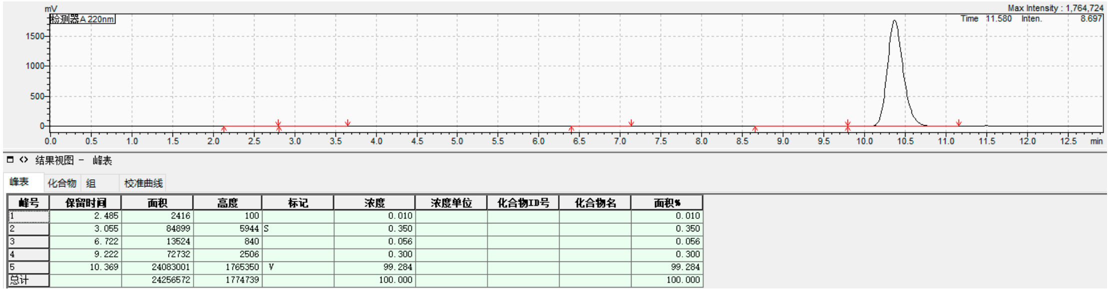

峰号	保留时间	面积	高度	标记	浓度	浓度单位	化合物ID号	化合物名	面积%
1	2. 485	2416	100		0. 010				0. 010
2	3. 055	84899	5944	S	0. 350				0. 350
3	6. 722	13524	840		0. 056				0. 056
4	9. 222	72732	2506		0. 300				0. 300
5	10. 369	24083001	1765350	V	99. 284				99. 284
总计		24256572	1774739		100. 000				100. 000

HPLC of compound **F16**

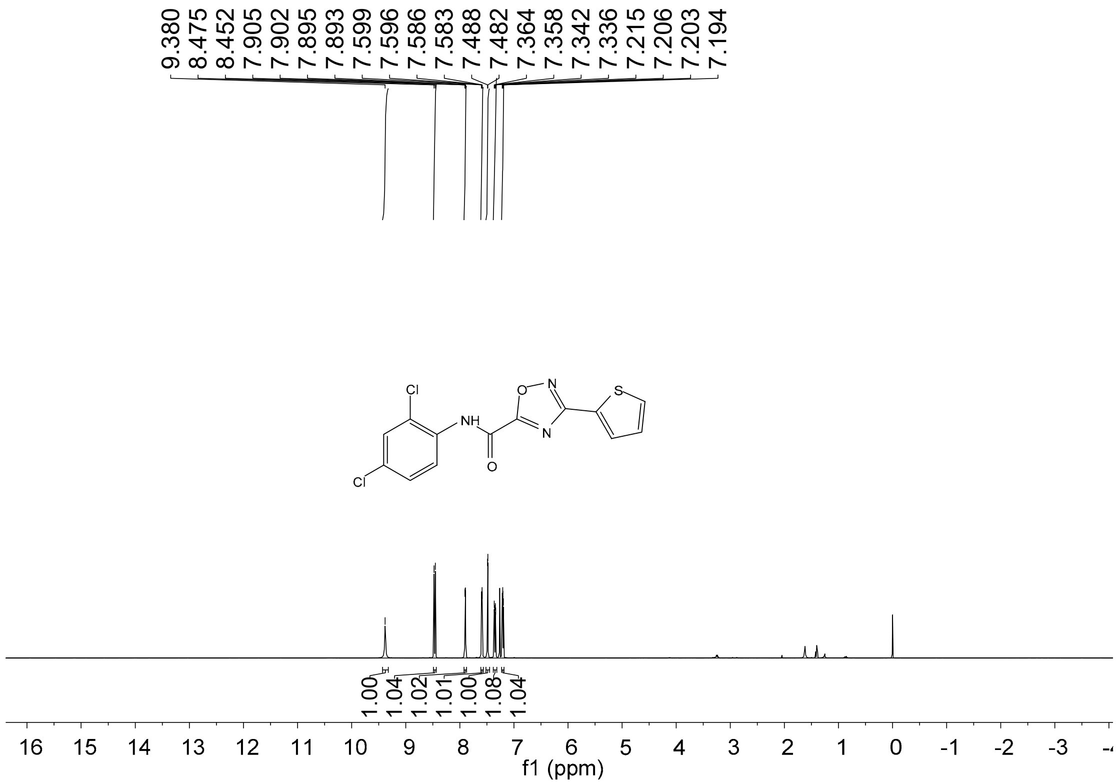

^{1}H NMR of compound **F16**

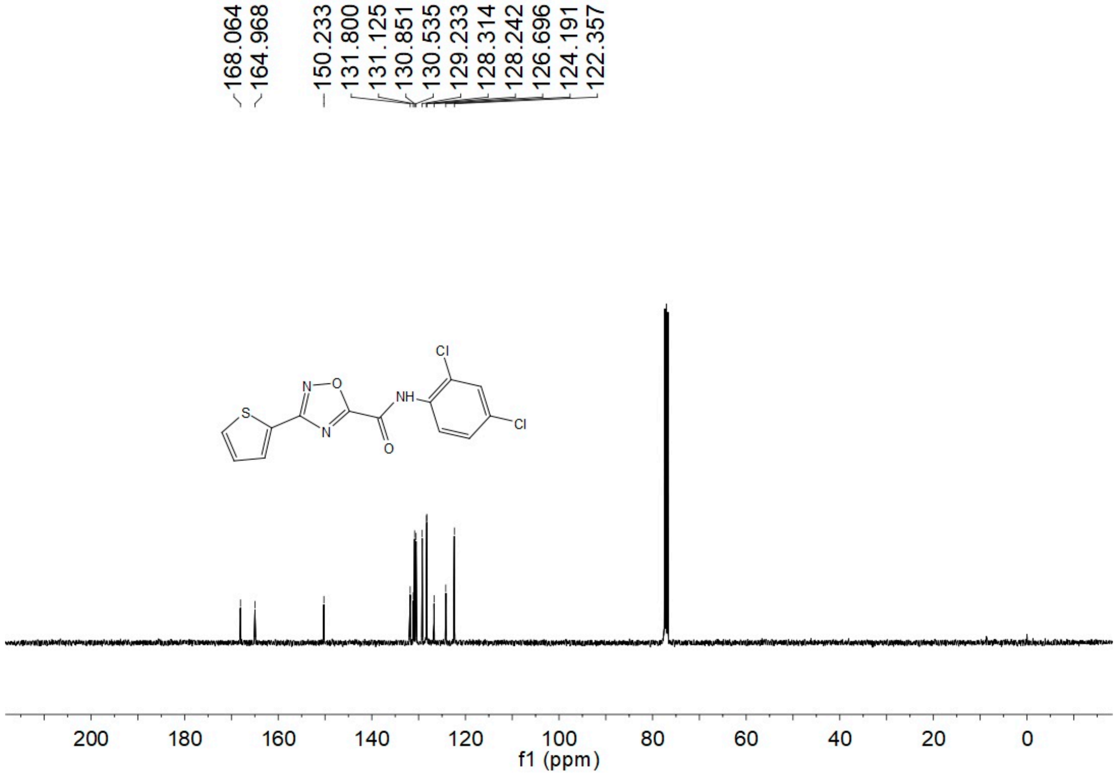

^{13}C NMR of compound **F16**

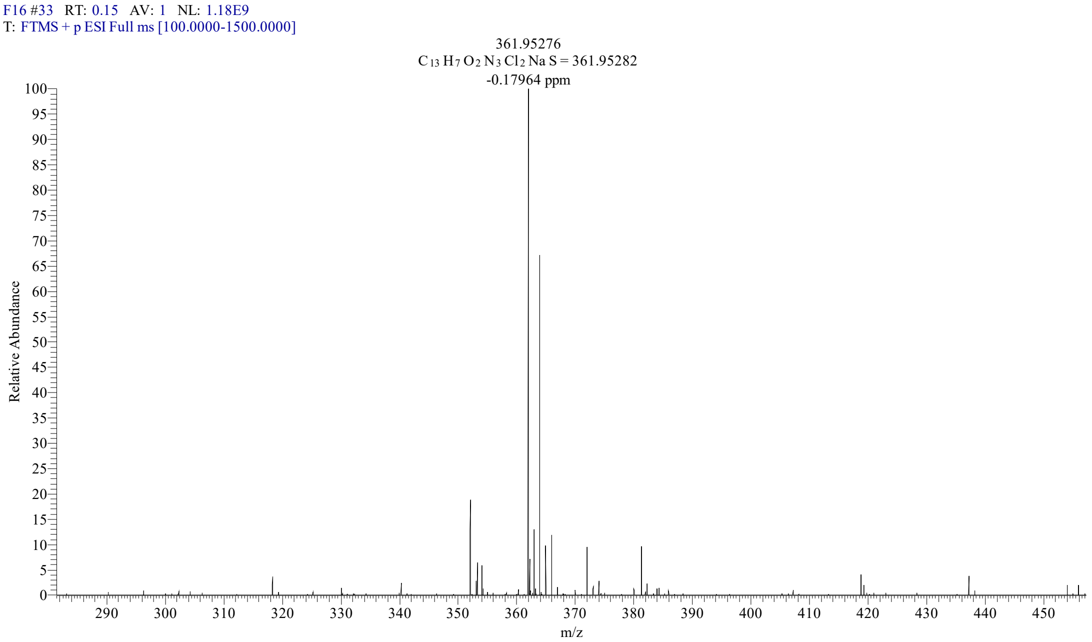

HRMS of compound **F16**

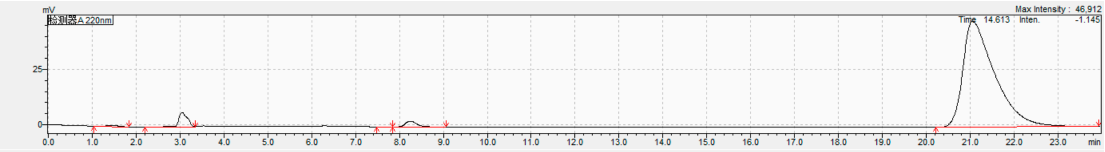

结果视图 - 峰表

峰表 化合物 组 校准曲线

峰号	保留时间	面积	高度	标记	浓度	浓度单位	化合物ID号	化合物名	面积%
1	1.458	12346	682		0.476				0.476
2	3.052	90749	6370		3.498				3.498
3	7.767	3232	194		0.125				0.125
4	8.251	53559	2709	V	2.065				2.065
5	21.059	2434144	48080		93.836				93.836
总计		2594031	58035		100.000				100.000

HPLC of compound **F17**

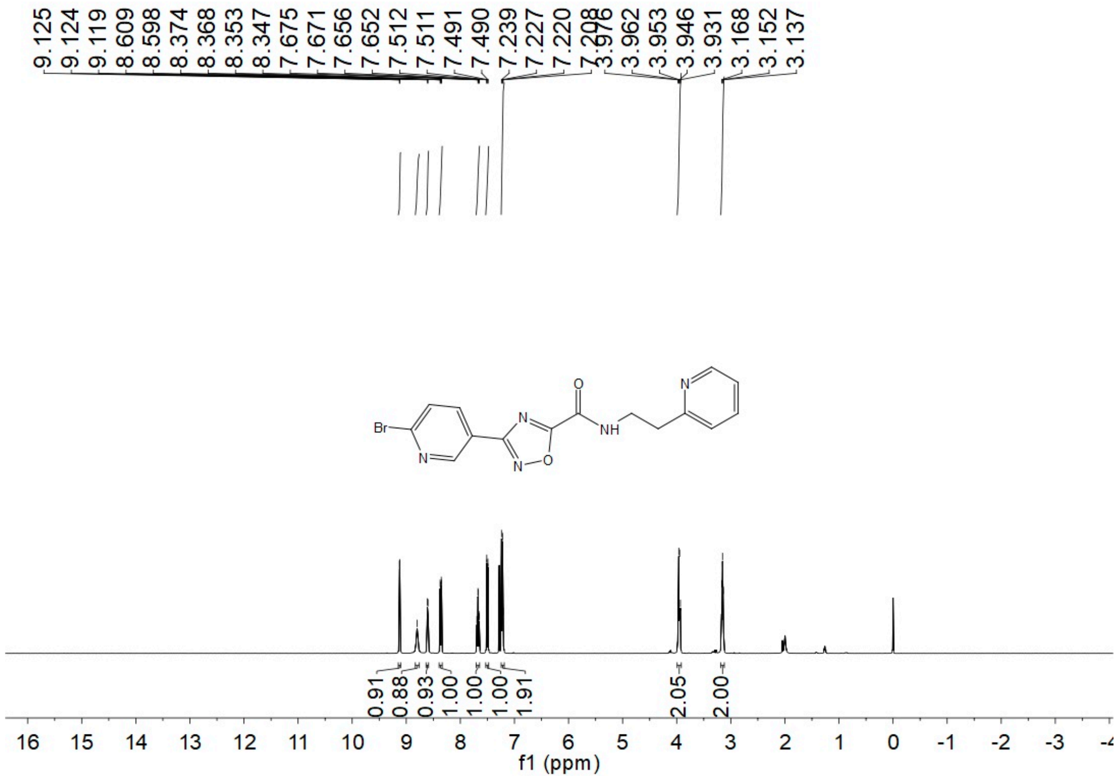

^{1}H NMR of compound **F17**

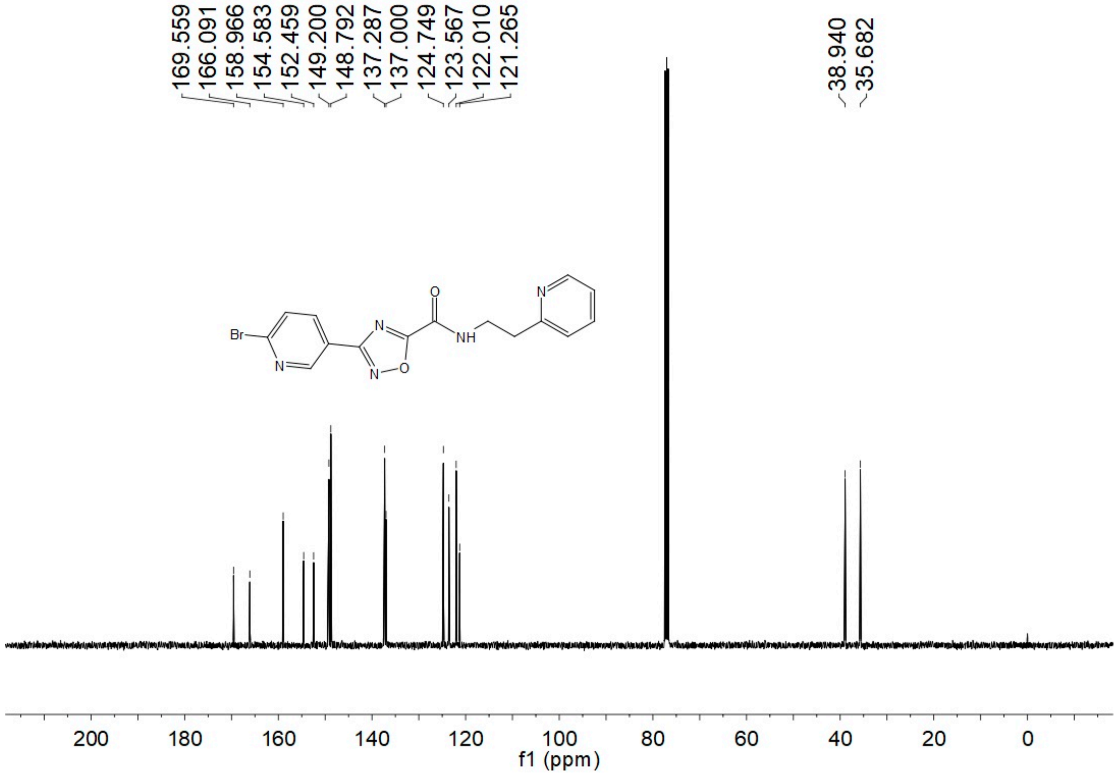

^{13}C NMR of compound **F17**

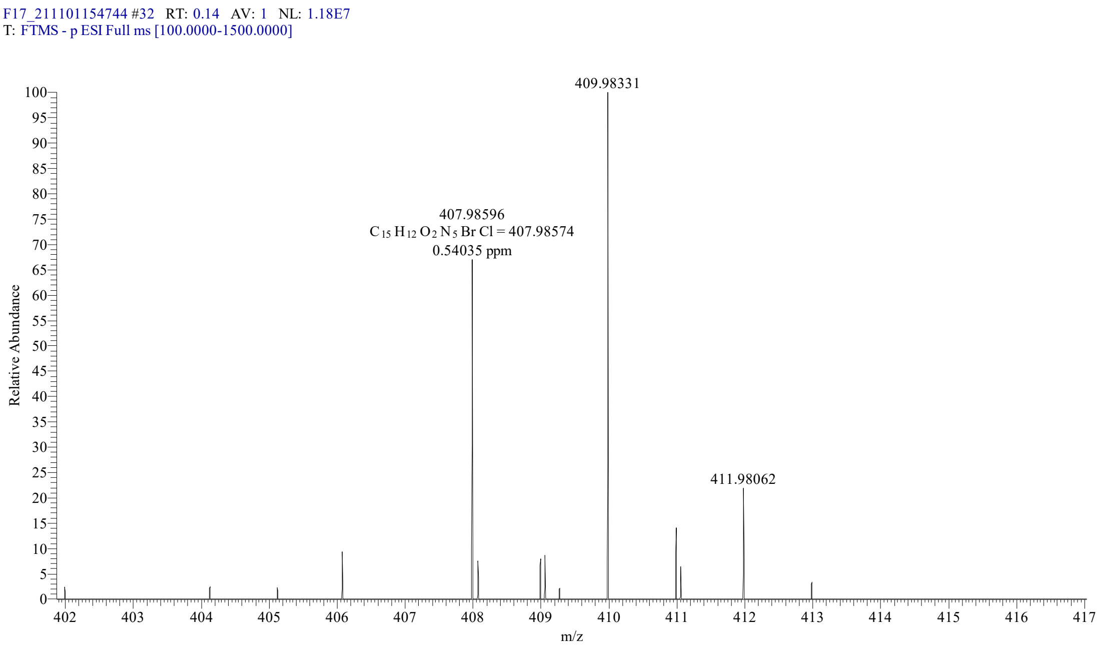

HRMS of compound **F17**

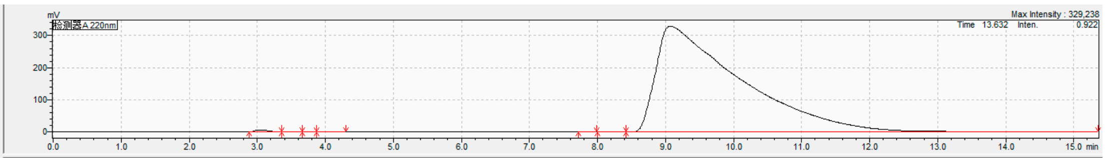

结果视图 - 峰表

峰表 化合物 组 校准曲线

峰号	保留时间	面积	高度	标记	浓度	浓度单位	化合物ID号	化合物名	面积%
2	3.546	4554	397	V	0.016				0.016
3	3.721	2006	180	V	0.007				0.007
4	4.010	3671	193	V	0.013				0.013
5	7.776	6063	675		0.021				0.021
6	8.217	3732	192		0.013				0.013
7	9.073	28576529	329626	V	99.640				99.640
总计		28679719	337206		100.000				100.000

HPLC of compound **F18**

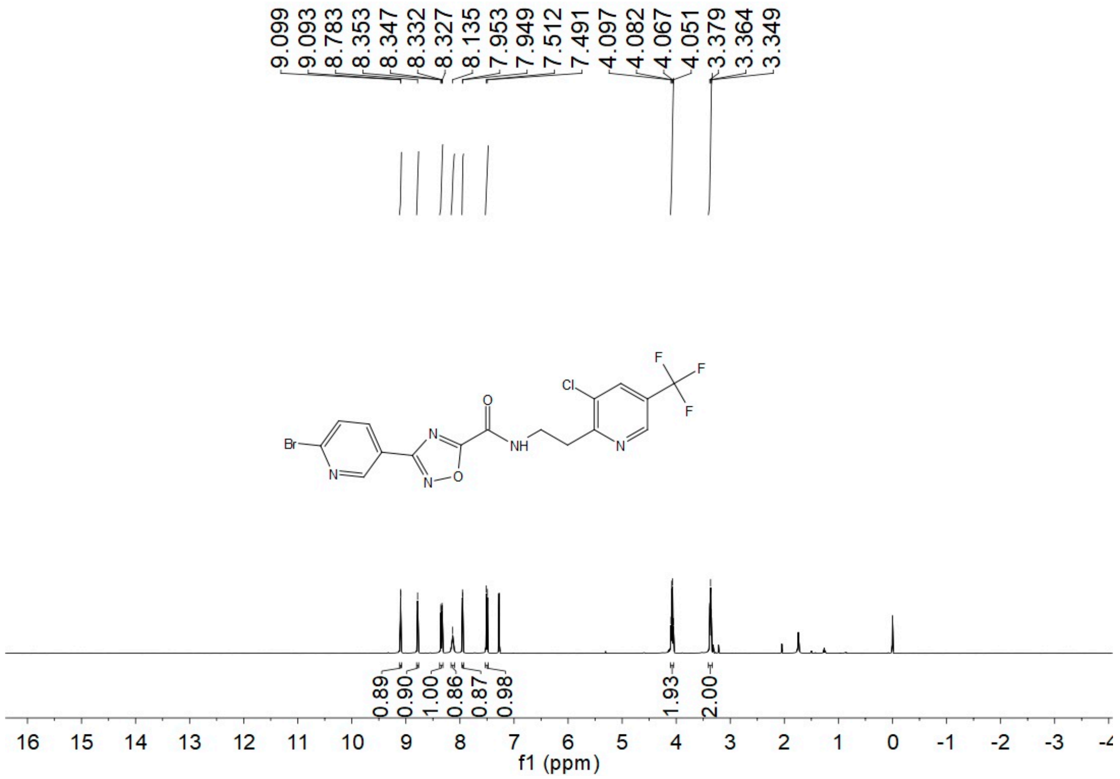

^{1}H NMR of compound **F18**

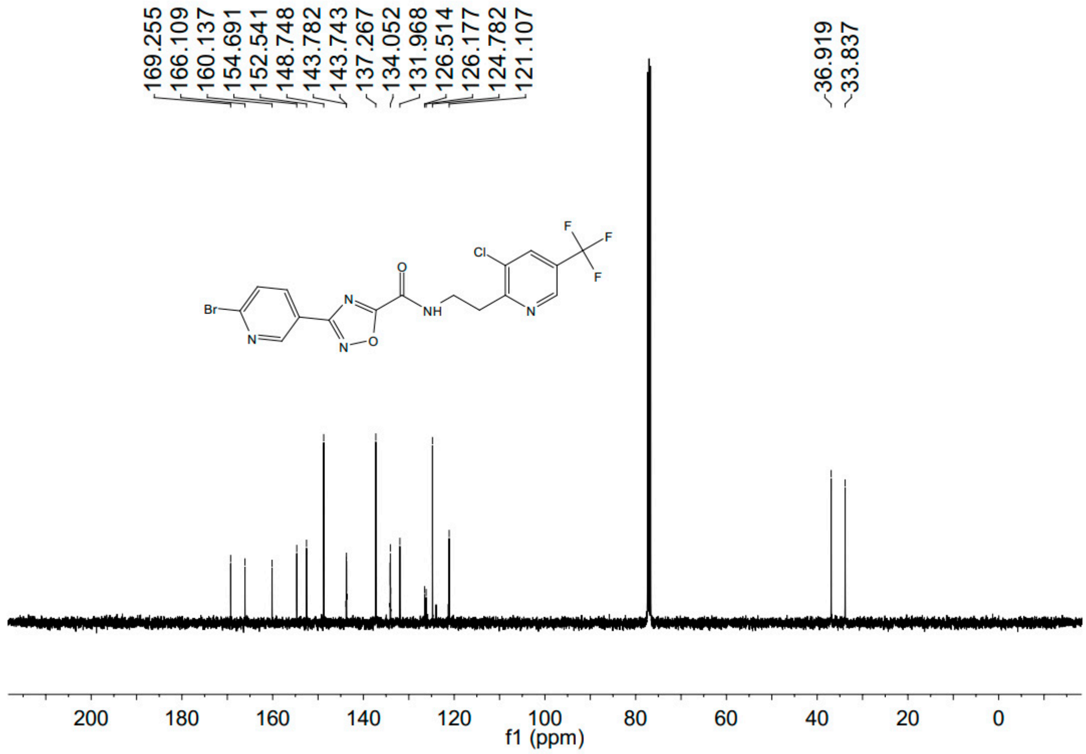

^{13}C NMR of compound **F18**

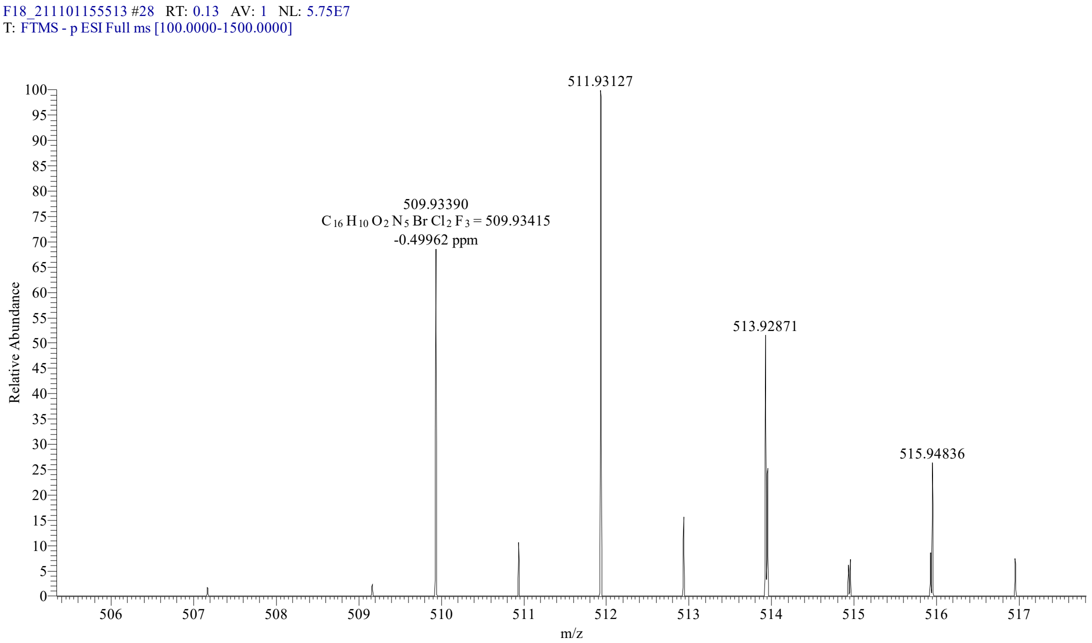

HRMS of compound **F18**

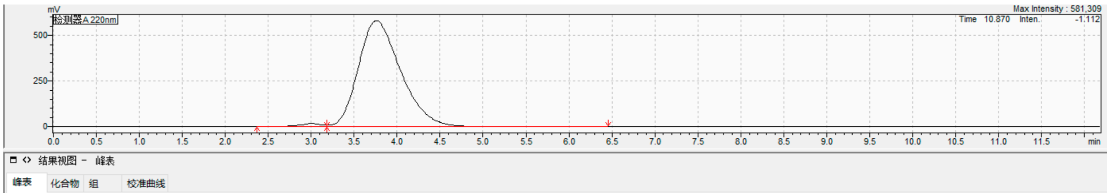

峰号	保留时间	面积	高度	标记	浓度	浓度单位	化合物ID号	化合物名	面积%
1	2.990	307705	18271		1.519				1.519
2	3.759	19944441	581750	V	98.481				98.481
总计		20252147	600021		100.000				100.000

HPLC of compound **F19**

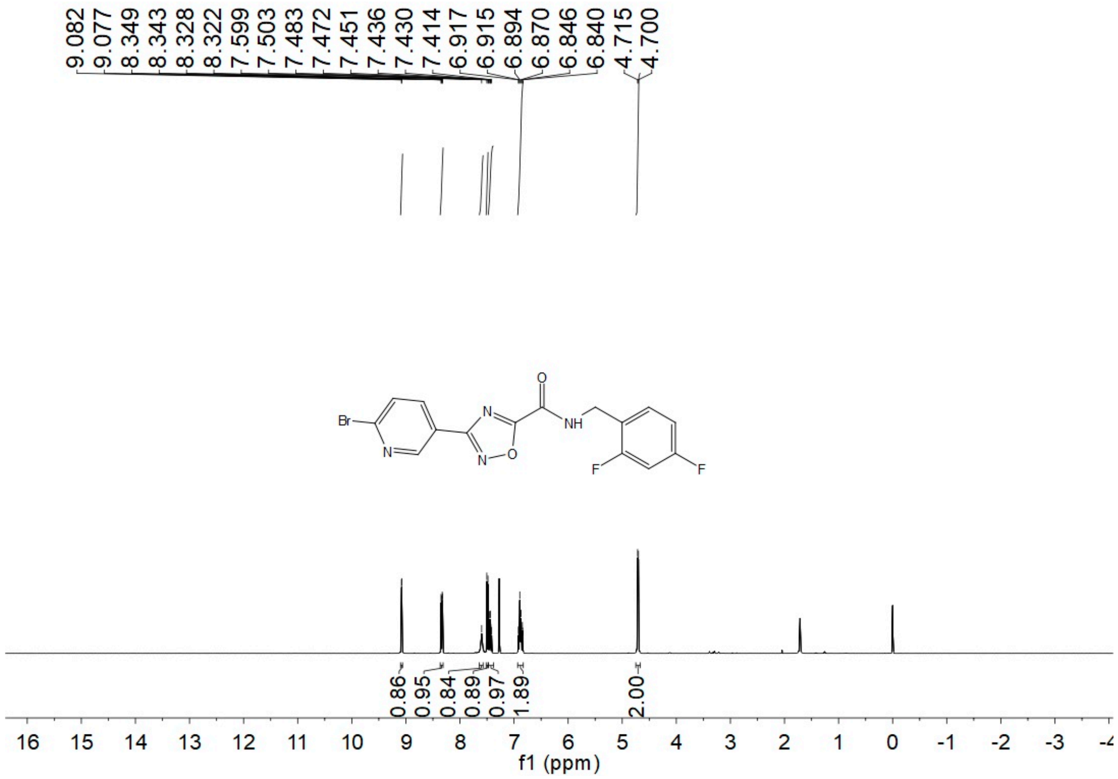

^{1}H NMR of compound **F19**

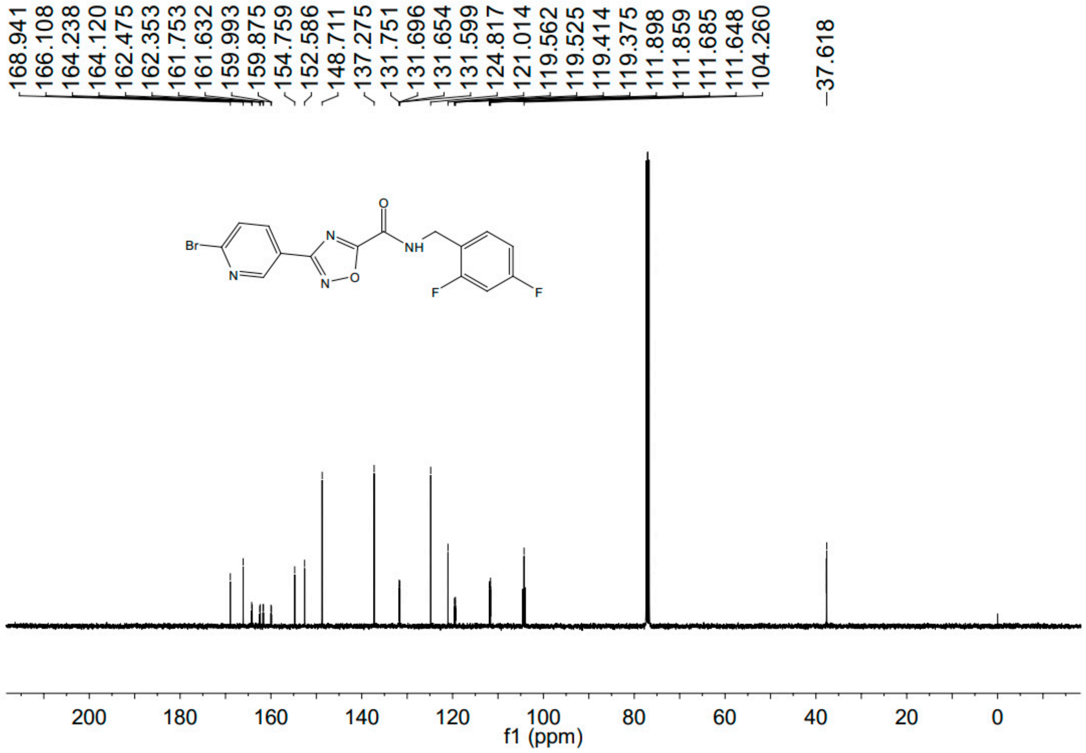

^{13}C NMR of compound **F19**

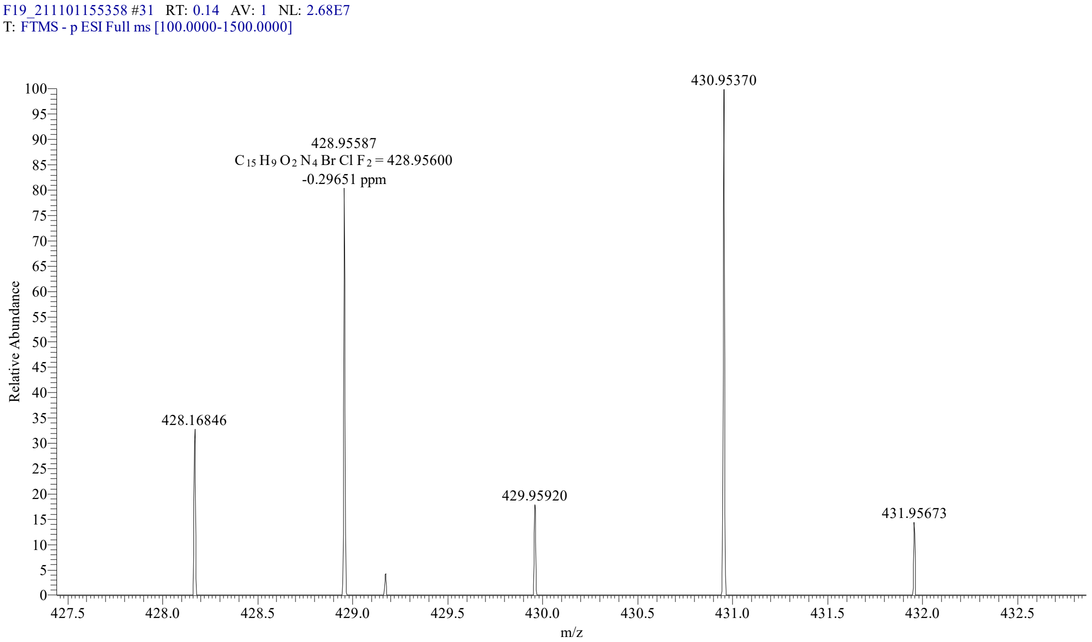

HRMS of compound **F19**

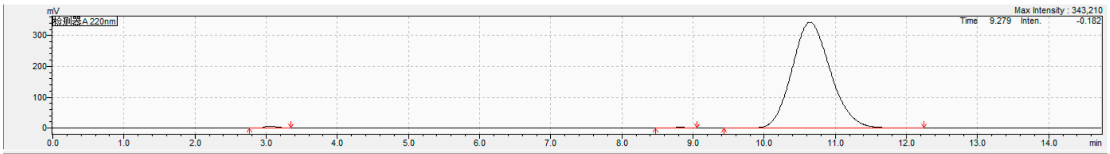

结果视图 - 峰表

峰表 化合物 组 校准曲线

峰号	保留时间	面积	高度	标记	浓度	浓度单位	化合物ID号	化合物名	面积%
1	3.058	80251	5779		0.612				0.612
2	8.821	46067	2425		0.351				0.351
3	10.644	12992980	343635		99.037				99.037
总计		13119298	351839		100.000				100.000

HPLC of compound **F20**

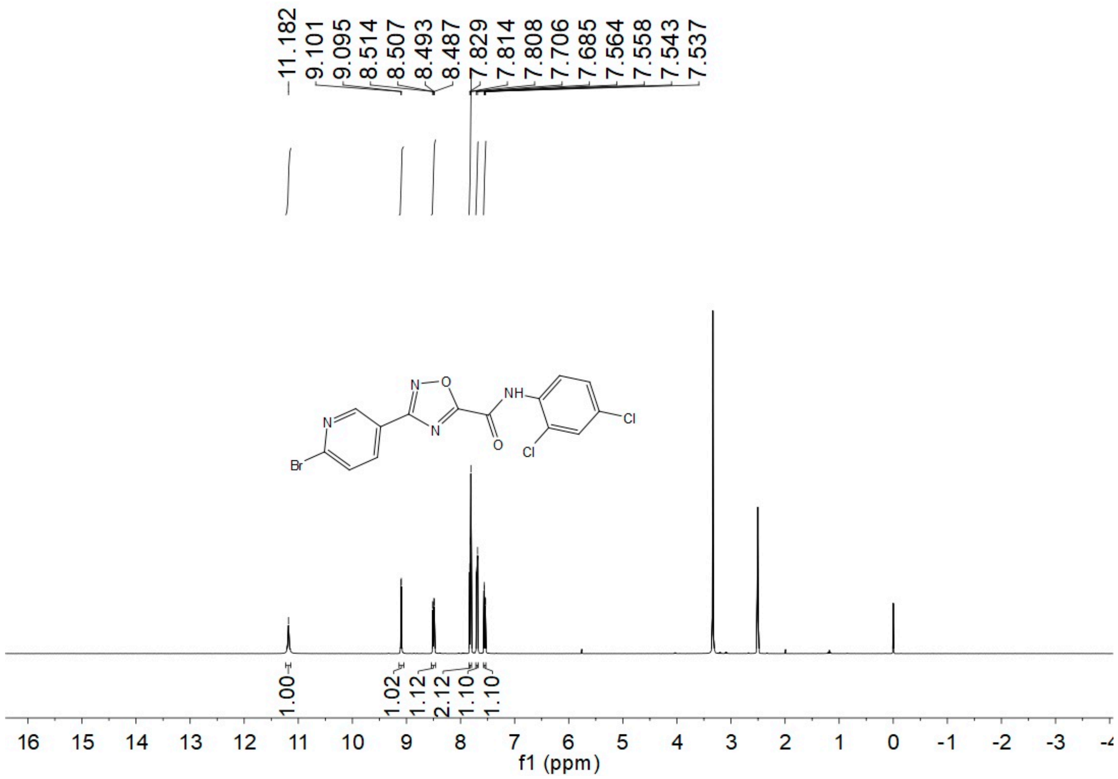

^{1}H NMR of compound **F20**

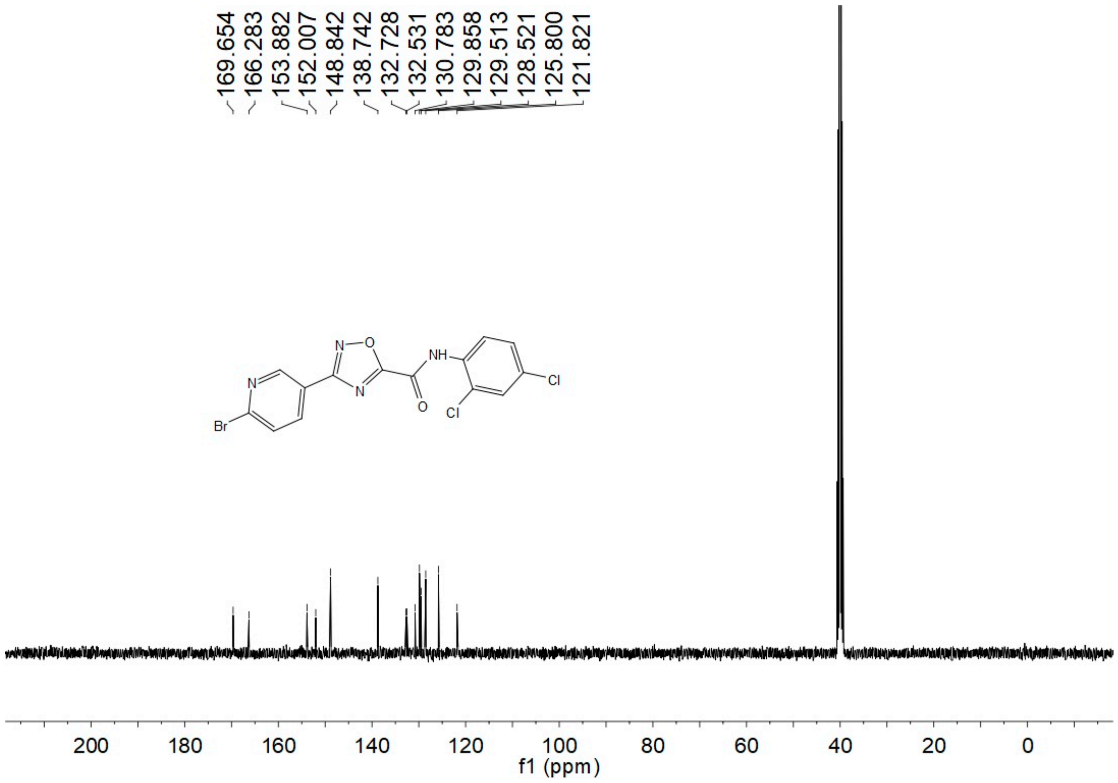

^{13}C NMR of compound **F20**

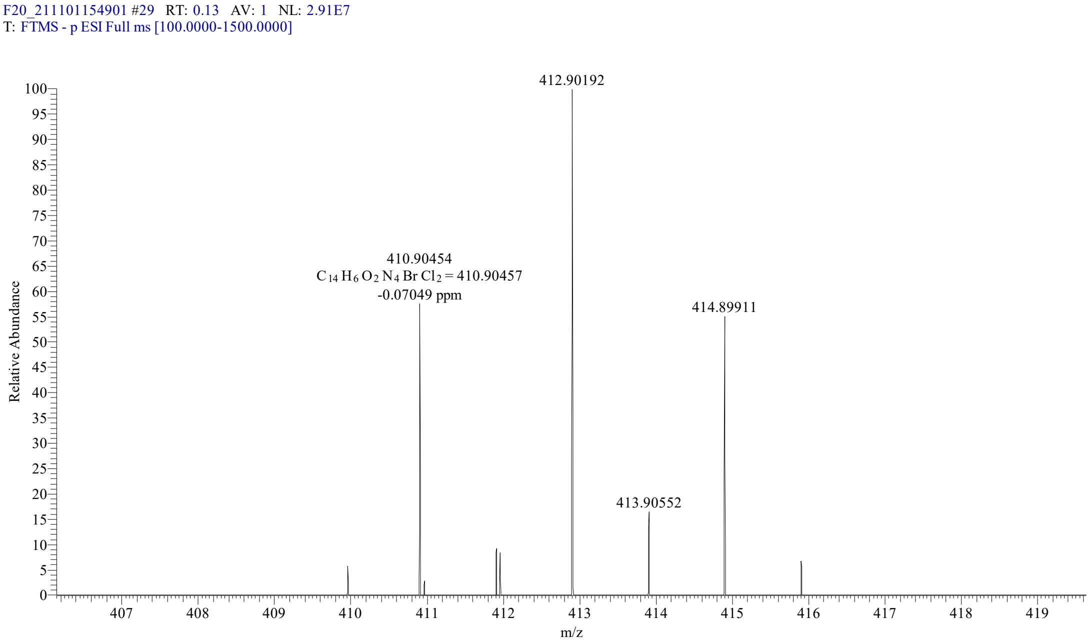

HRMS of compound **F20**

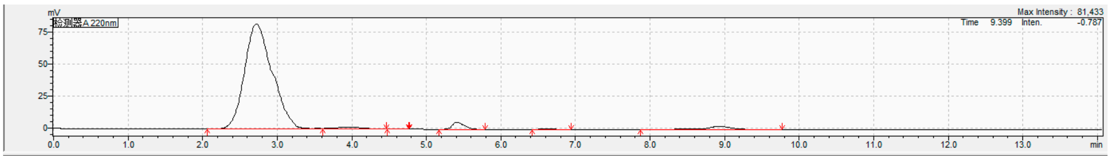

结果视图 - 峰表

峰表 化合物 组 校准曲线

峰号	保留时间	面积	高度	标记	浓度	浓度单位	化合物ID号	化合物名	面积%
1	2. 722	2156817	82028	S	92. 423				92. 423
2	3. 971	29853	1215	TV	1. 279				1. 279
3	4. 590	1308	139	T	0. 056				0. 056
4	5. 411	60837	5602		2. 607				2. 607
5	6. 557	4055	316		0. 174				0. 174
6	8. 932	80772	2631		3. 461				3. 461
总计		2333641	91932		100. 000				100. 000

HPLC of compound **F21**

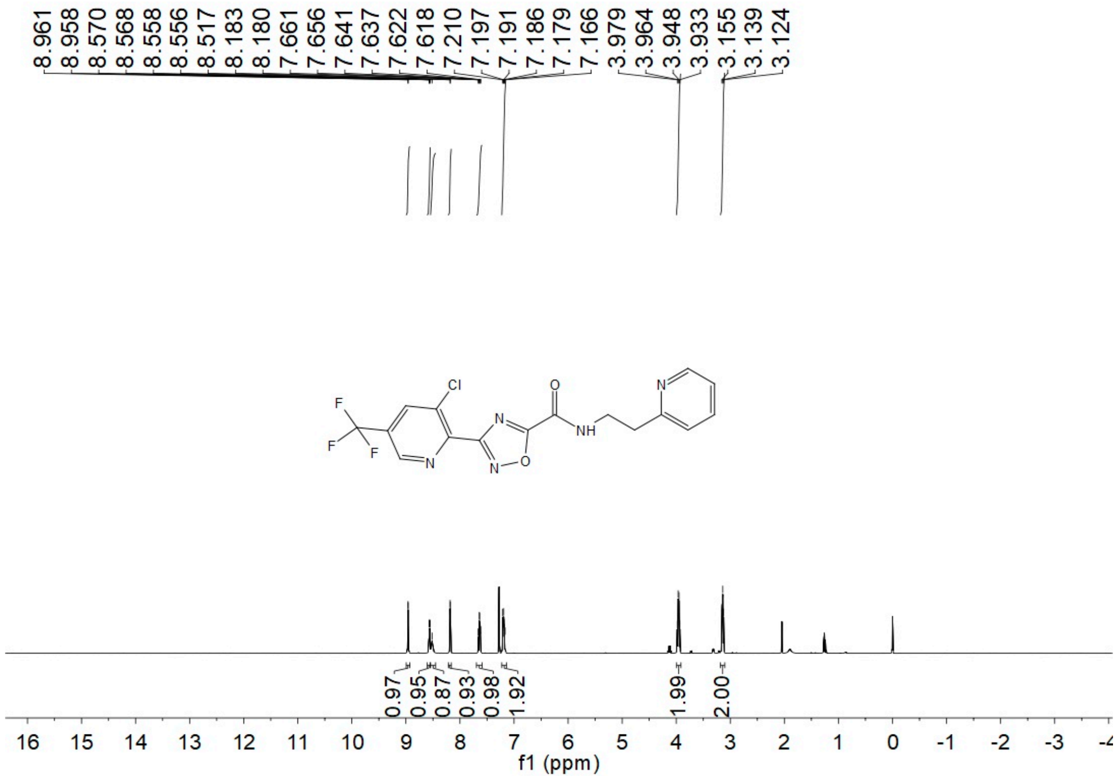

^{1}H NMR of compound **F21**

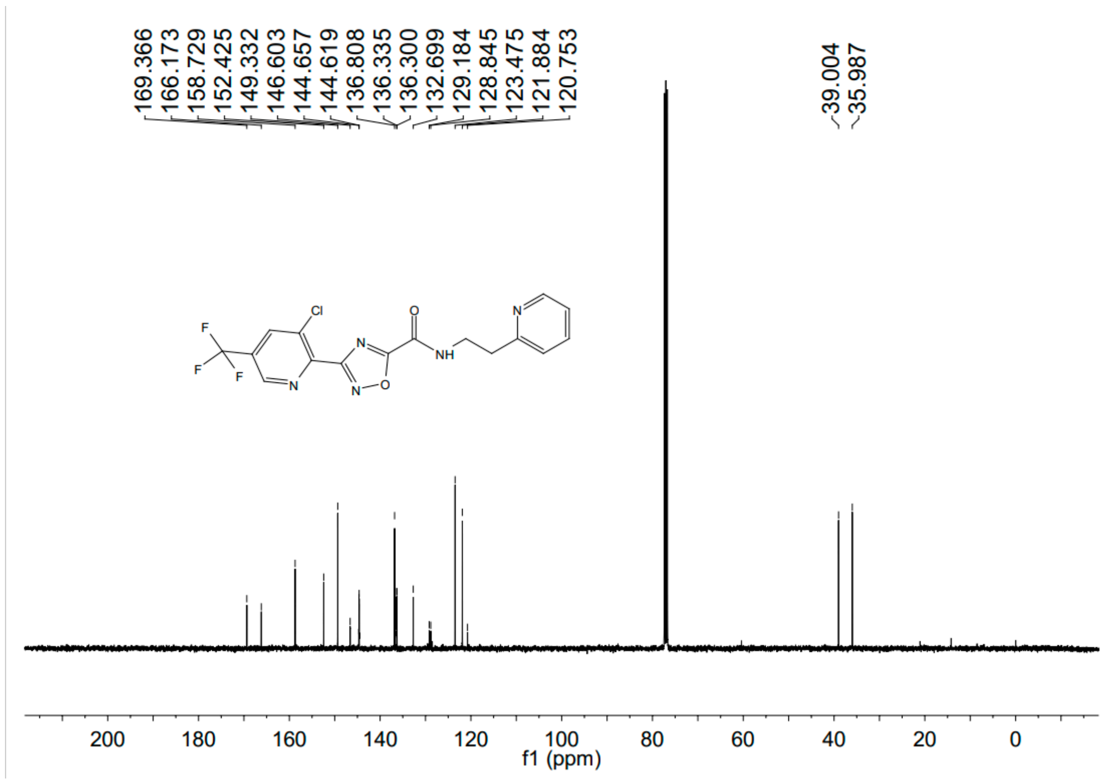

^{13}C NMR of compound **F21**

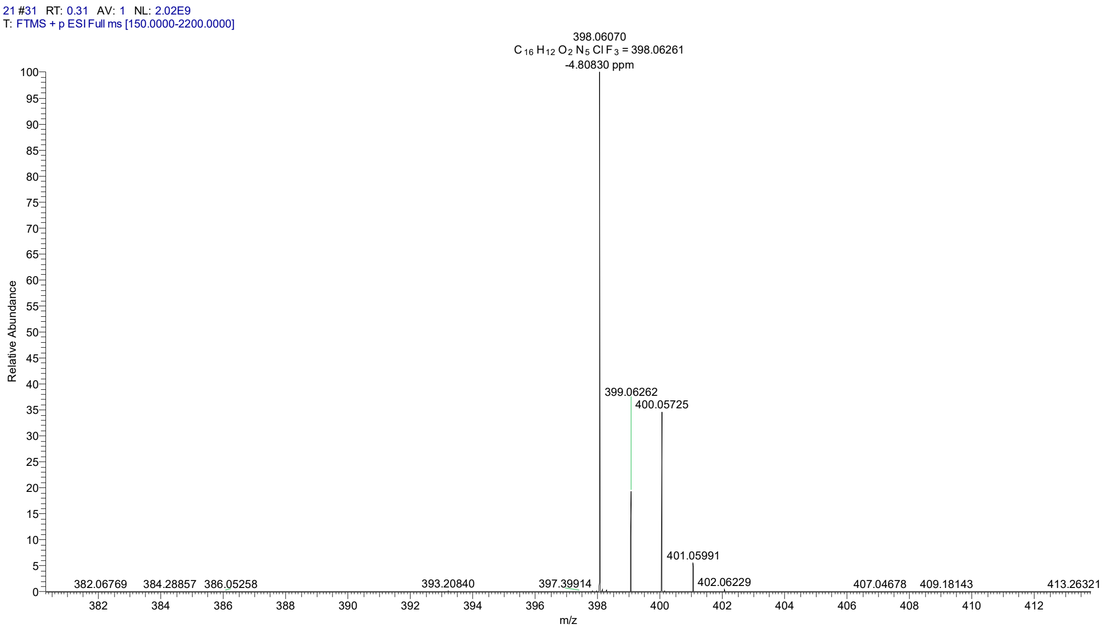

HRMS of compound **F21**

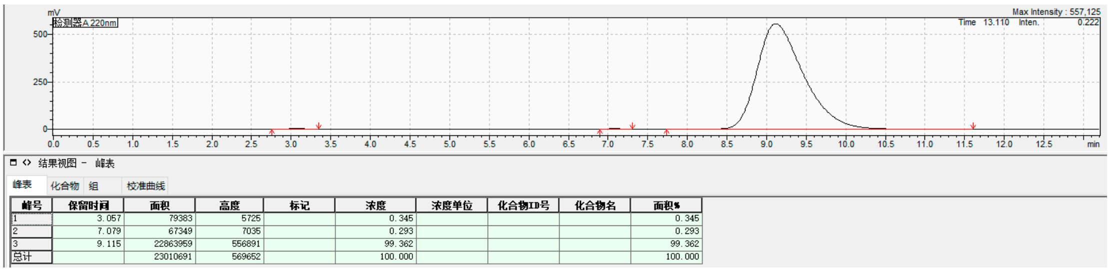

峰号	保留时间	面积	高度	标记	浓度	浓度单位	化合物ID号	化合物名	面积%
1	3.057	79383	5725		0.345				0.345
2	7.079	67349	7035		0.293				0.293
3	9.115	22863959	556891		99.362				99.362
总计		23010691	569652		100.000				100.000

HPLC of compound **F22**

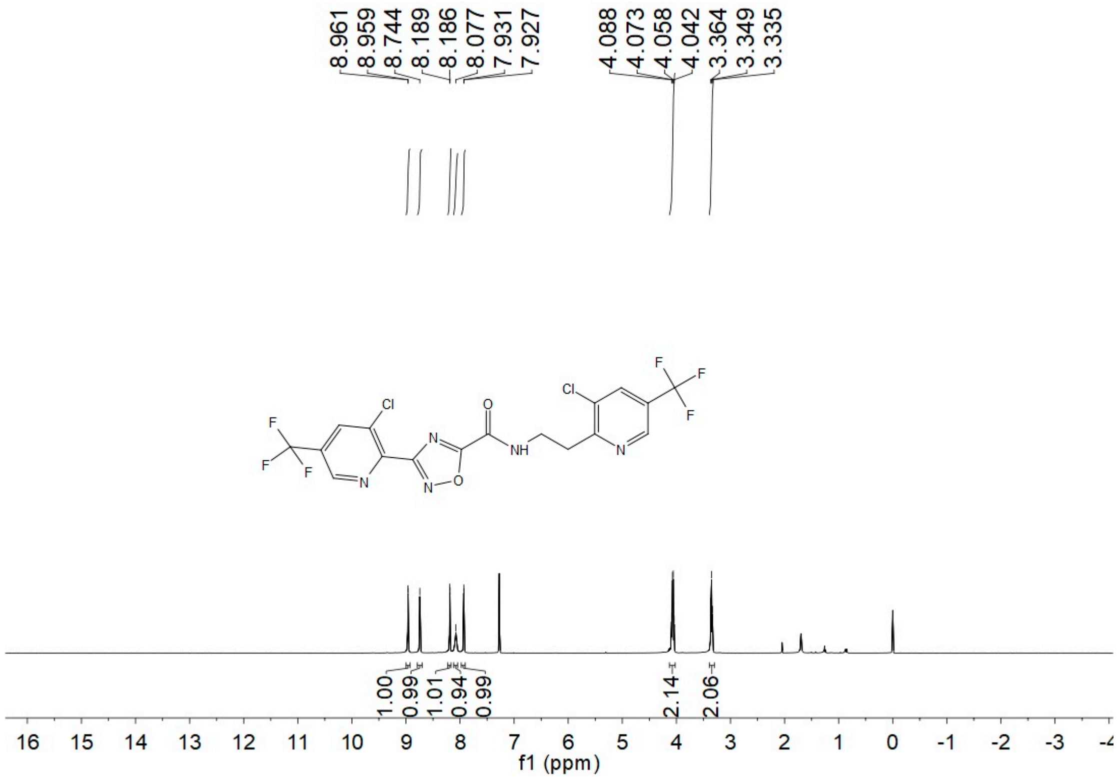

^{1}H NMR of compound **F22**

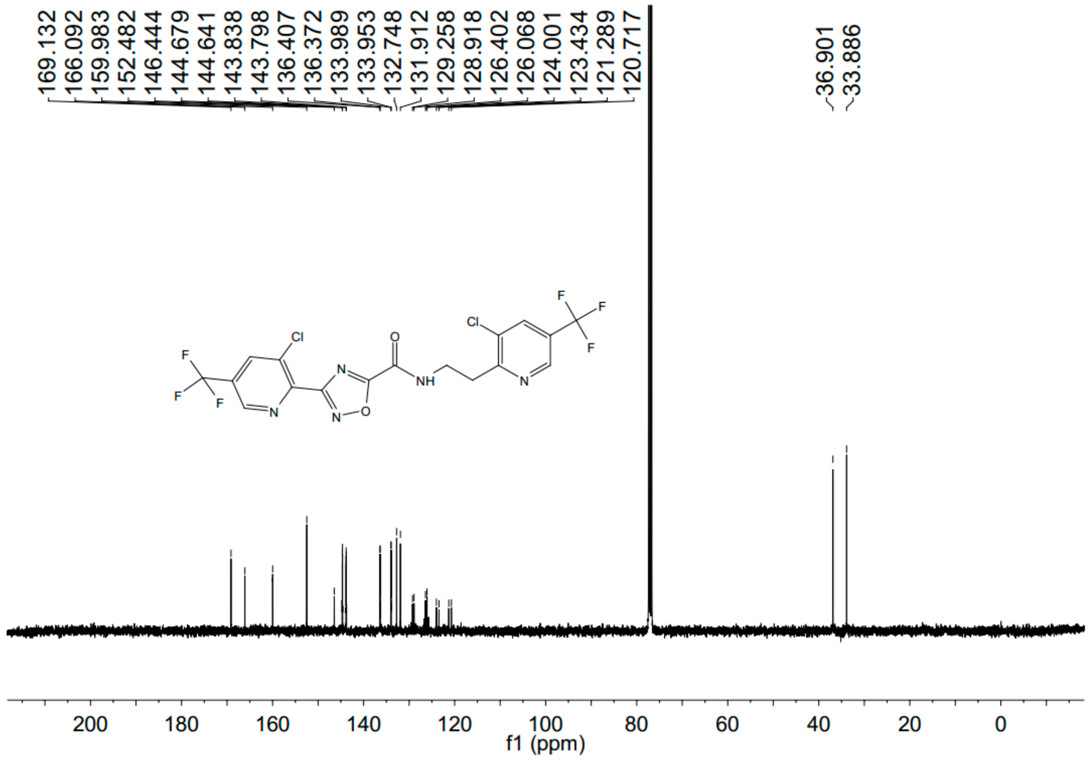

^{13}C NMR of compound **F22**

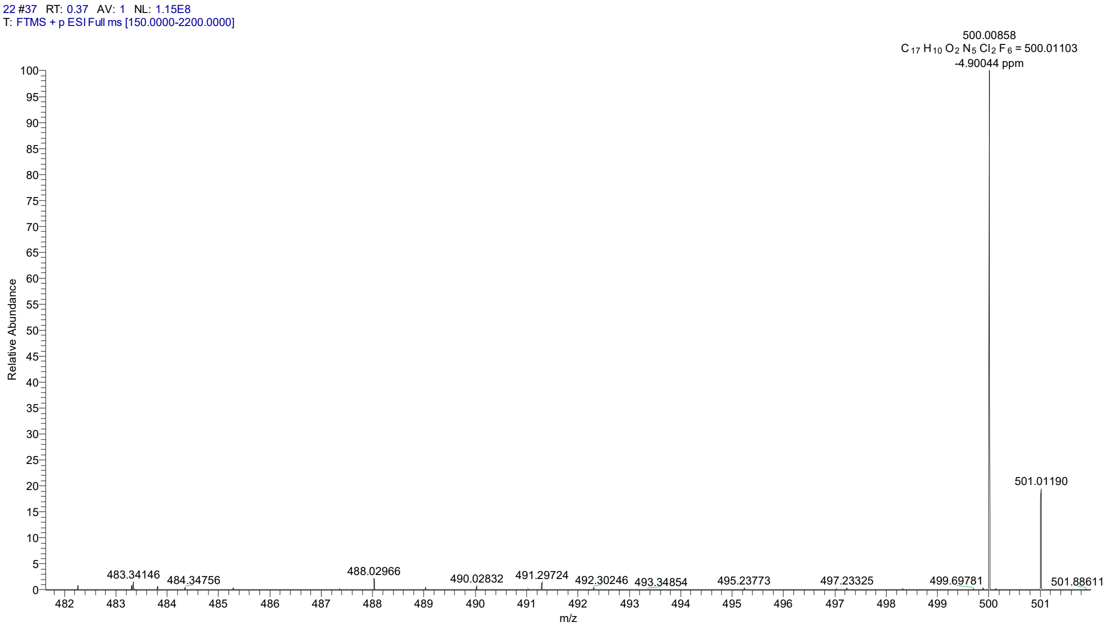

HRMS of compound **F22**

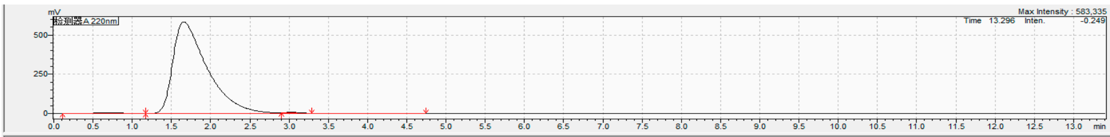

结果视图 – 峰表

峰表 化合物 组 校准曲线

峰号	保留时间	面积	高度	标记	浓度	浓度单位	化合物ID号	化合物名	面积%
1	0.680	178578	7747		0.981				0.981
2	1.658	17954012	583333	SV	98.604				98.604
3	2.998	75687	5549	T	0.416				0.416
总计		18208277	596629		100.000				100.000

HPLC of compound **F23**

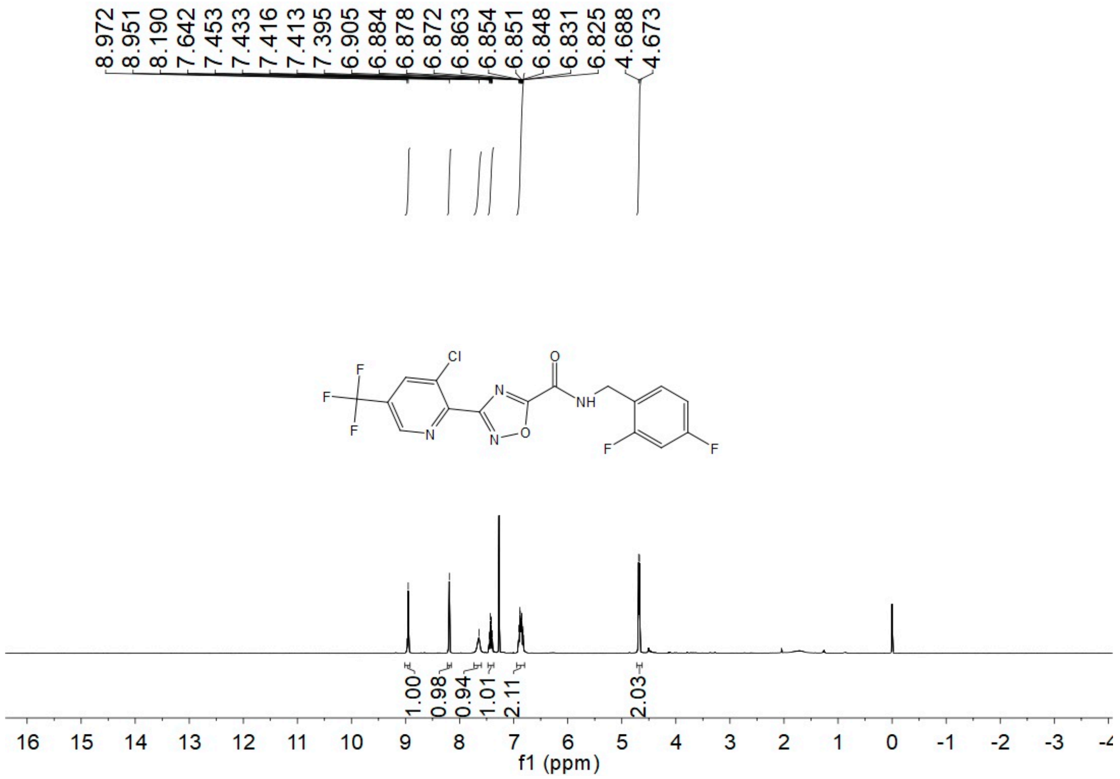

^{1}H NMR of compound **F23**

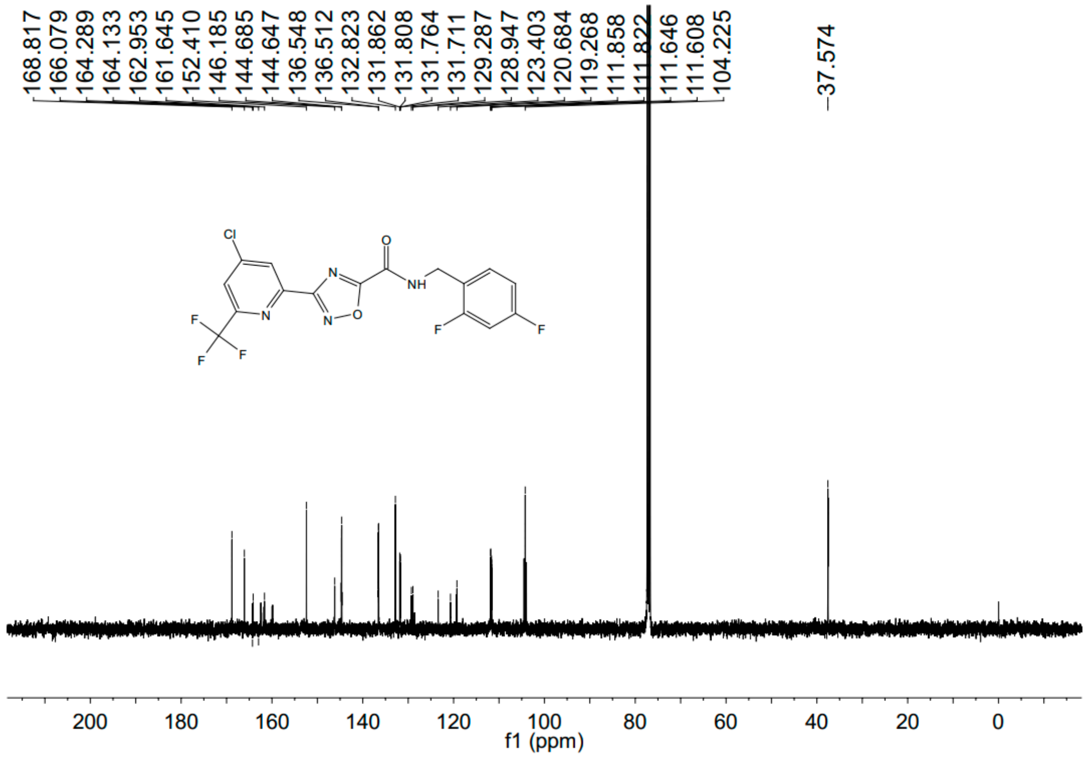

^{13}C NMR of compound **F23**

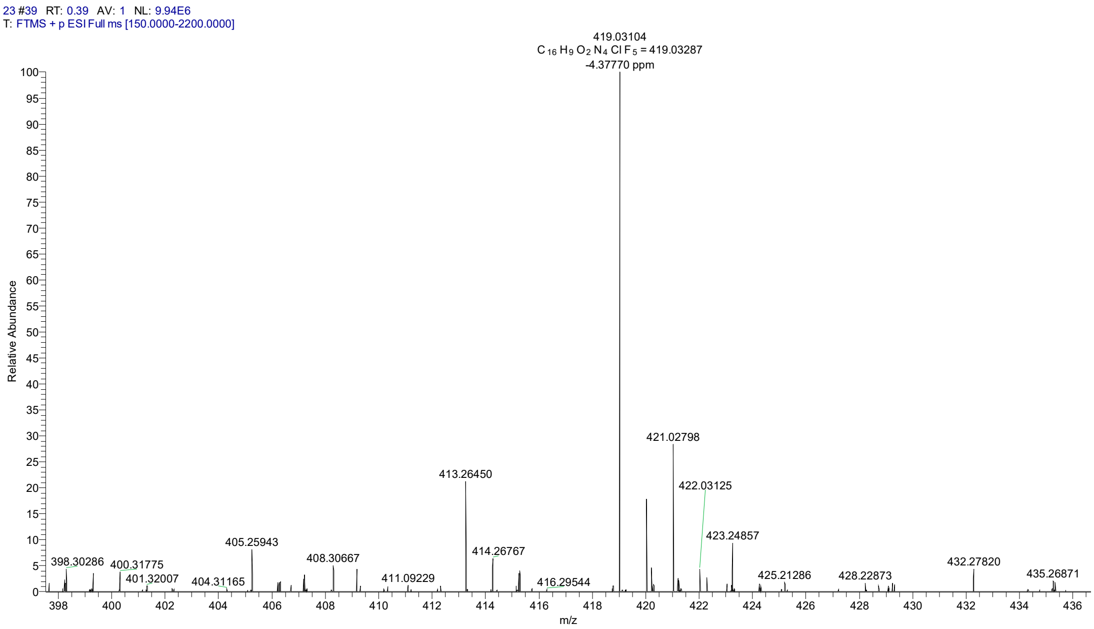

HRMS of compound **F23**

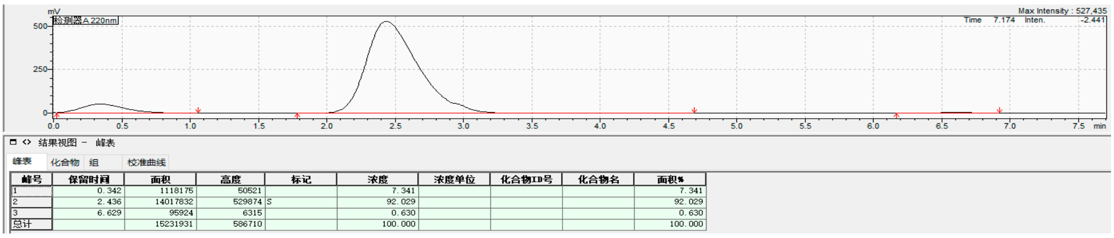

峰号	保留时间	面积	高度	标记	浓度	浓度单位	化合物ID号	化合物名	面积%
1	0. 342	1118175	50521		7. 341				7. 341
2	2. 436	14017832	529874	S	92. 029				92. 029
3	6. 629	95924	6315		0. 630				0. 630
总计		15231931	586710		100. 000				100. 000

HPLC of compound **F24**

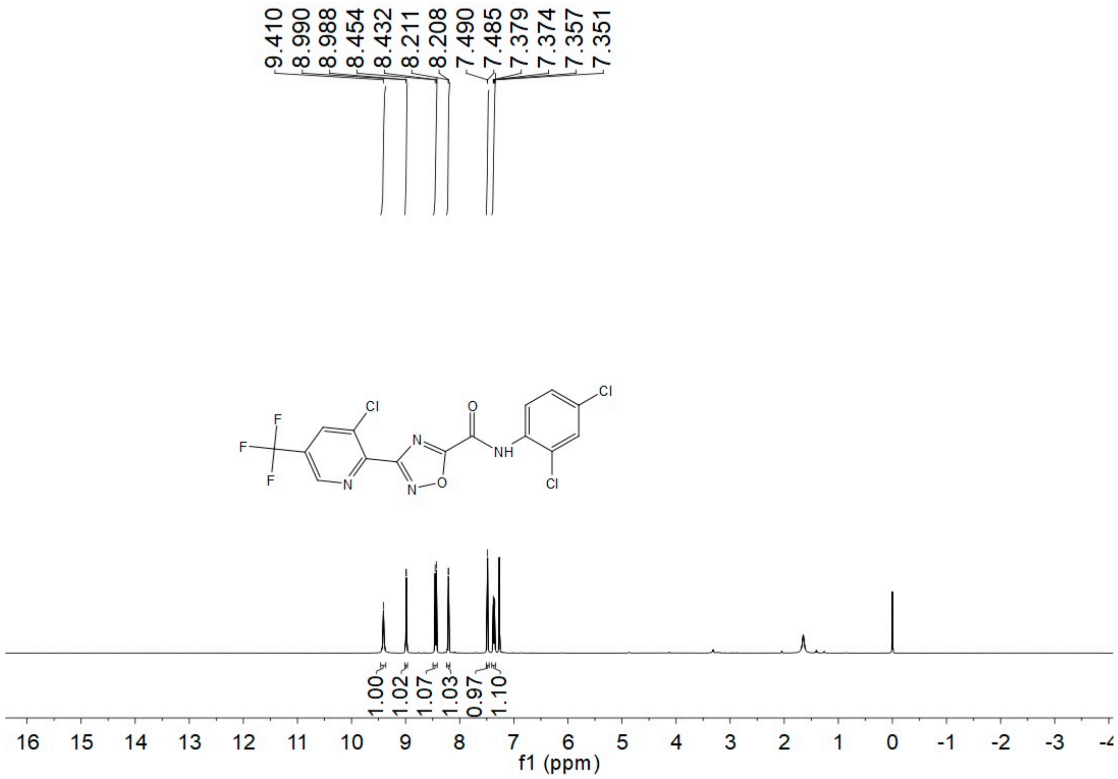

^{1}H NMR of compound **F24**

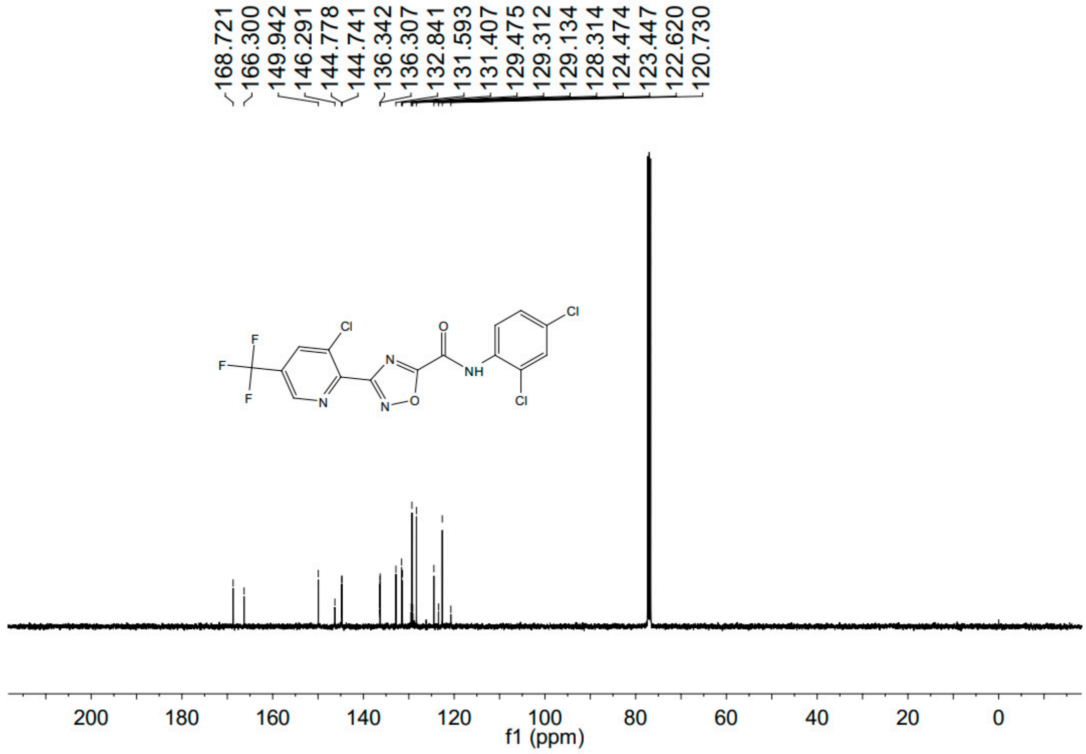

^{13}C NMR of compound F24

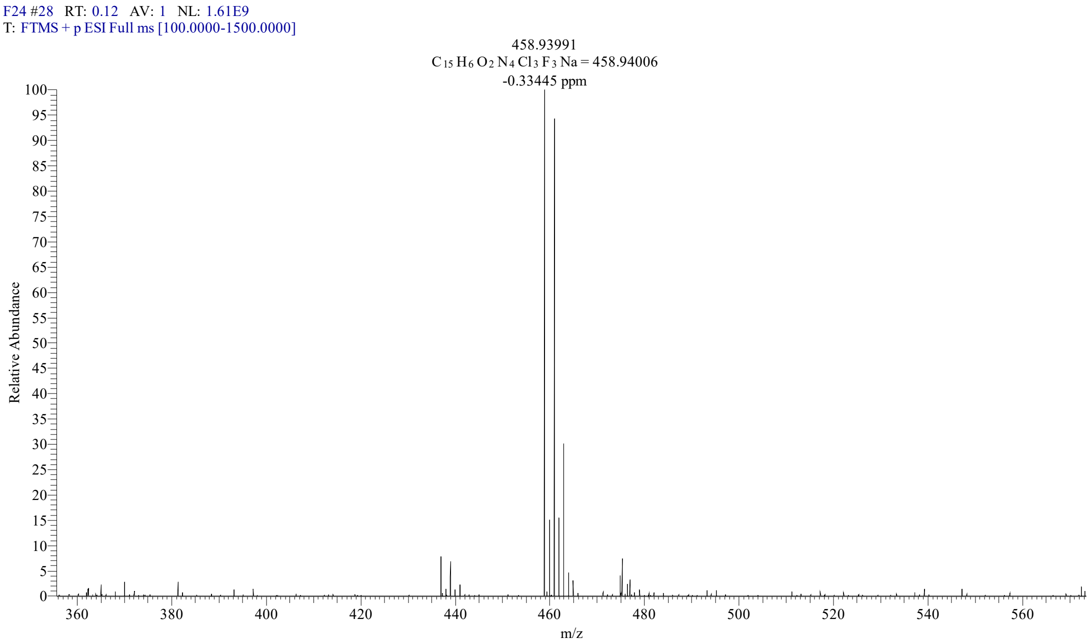

HRMS of compound **F24**
